# Interplay between demographic, clinical and polygenic risk factors for severe COVID-19

**DOI:** 10.1093/ije/dyac137

**Published:** 2022-06-30

**Authors:** Samantha S R Crossfield, Natalie J M Chaddock, Mark M Iles, Mar Pujades-Rodriguez, Ann W Morgan

**Affiliations:** School of Medicine and Leeds Institute for Data Analytics, University of Leeds, Leeds, UK; School of Medicine and Leeds Institute for Data Analytics, University of Leeds, Leeds, UK; School of Medicine and Leeds Institute for Data Analytics, University of Leeds, Leeds, UK; School of Medicine and Leeds Institute for Data Analytics, University of Leeds, Leeds, UK; School of Medicine and Leeds Institute for Data Analytics, University of Leeds, Leeds, UK; NIHR Leeds Biomedical Research Centre and NIHR Leeds Medtech and In vitro Diagnostics Co-Operative, Leeds Teaching Hospitals NHS Trust, Leeds, UK

**Keywords:** COVID-19, polygenic risk score, epidemiology, risk prediction

## Abstract

**Background:**

We aimed to identify clinical, socio-demographic and genetic risk factors for severe COVID-19 (hospitalization, critical care admission or death) in the general population.

**Methods:**

In this observational study, we identified 9560 UK Biobank participants diagnosed with COVID-19 during 2020. A polygenic risk score (PRS) for severe COVID-19 was derived and optimized using publicly available European and trans-ethnic COVID-19 genome-wide summary statistics. We estimated the risk of hospital or critical care admission within 28 days or death within 100 days following COVID-19 diagnosis, and assessed associations with socio-demographic factors, immunosuppressant use and morbidities reported at UK Biobank enrolment (2006–2010) and the PRS. To improve biological understanding, pathway analysis was performed using genetic variants comprising the PRS.

**Results:**

We included 9560 patients followed for a median of 61 (interquartile range = 34–88) days since COVID-19 diagnosis. The risk of severe COVID-19 increased with age and obesity, and was higher in men, current smokers, those living in socio-economically deprived areas, those with historic immunosuppressant use and individuals with morbidities and higher co-morbidity count. An optimized PRS, enriched for single-nucleotide polymorphisms in multiple immune-related pathways, including the ‘oligoadenylate synthetase antiviral response’ and ‘interleukin-10 signalling’ pathways, was associated with severe COVID-19 (adjusted odds ratio 1.32, 95% CI 1.11–1.58 for the highest compared with the lowest PRS quintile).

**Conclusion:**

This study conducted in the pre-SARS-CoV-2-vaccination era, emphasizes the novel insights to be gained from using genetic data alongside commonly considered clinical and socio-demographic factors to develop greater biological understanding of severe COVID-19 outcomes.

Key Messages

- We derived and optimized a polygenic risk score (PRS) that was found to be associated with severe COVID-19 and death in both White European and trans-ethnic populations, after adjusting for clinico-demographic factors reported at UK Biobank enrolment (2006–2010).
- We identified antiviral and immunoregulatory immune pathways that were associated with COVID-19 disease severity.
- The magnitude of risk for the highest PRS quintile was equivalent to that reported for well-known risk factors, such as living in the most deprived areas or having cardiovascular disease.
- In addition to known risk factors, risk of severe COVID-19 was increased in immunosuppressant users, patients with autoimmune diseases, those with a higher co-morbidity count, as well as a positive association with each additional 5-kg/m² increment of body mass index between 25 and 40 kg/m².
- Genetic variation in immune pathways offers the opportunity for guiding therapeutic strategies in severe COVID-19 and ultimately may improve risk stratification for patients requiring immunosuppressant therapy.

## Introduction

Coronavirus disease 2019 (COVID-19), caused by severe acute respiratory syndrome coronavirus 2 (SARS-CoV-2), was first reported in December 2019^1^ and rapidly became a threat to public health, medical systems and economies worldwide. In 2020, ∼13% of patients diagnosed with COVID-19 (suspected or confirmed) in England required hospitalization, with a mortality rate of ∼5%.^2^

Risk factors for severe COVID-19, involving hospitalization or death, include older age, male sex, Black and minority ethnicity, current and prior smoking, high socio-economic deprivation and obesity.^3–5^ Morbidities that have been associated with poor COVID-19 outcomes in both population and hospital studies, include: cardiovascular disease (CVD), hypertension, diabetes, chronic respiratory disease (CRD), chronic kidney disease (CKD), malignancies and neurological disease, including dementia.^3^^,^^4^^,^^6^^,^^7^

It remains unclear whether patients with autoimmune disease are at increased risk of contracting SARS-CoV-2 infection or developing severe COVID-19 outcomes, which, in light of the need for immunosuppressants, is crucial to inform the optimal management of these conditions.^4^ More granular data on the risks associated with individual immunosuppressants have come from the Global Rheumatology Alliance.^8^

As healthcare systems increasingly embrace genomic medicine, there are opportunities to incorporate polygenic risk scores (PRSs) that summarize an individual’s genetic propensity to adverse treatment outcomes and disease complications into clinical decision-making. To date, published PRSs have primarily been developed in populations of White European ethnicity, limiting their ability to predict genetic risk in other ethnicities. Differences in linkage disequilibrium (LD) structure and allele frequencies between ethnic groups may reduce the efficacy of such PRSs in non-European individuals and careful optimization and testing are required.^9^

Several genome-wide association studies (GWASs), performed on both White European and trans-ethnic cohorts, have identified genome-wide significant (*P-*value < 5 × 10^–8^) associations with 27 genetic loci (to date) for a range of COVID-19 phenotypes.^10–13^ Many of these loci are associated with autoimmune diseases and lung function, corroborating a potential role for these pathways in an individual’s response to SARS-CoV-2.

In this study, we examined risk of hospitalization, critical care admission or death of UK Biobank participants diagnosed with COVID-19. We identified associated clinical and socio-demographic risk factors, including co-morbidities and historic immunosuppressant use. We then constructed a number of PRS using the largest COVID-19 susceptibility GWAS data sets (Release 5), provided by the COVID19-hg consortium,^10^ and evaluated the performance of our optimized PRS in independent UK Biobank data in conjunction with the clinico-demographic risk factors.

## Methods

The study was reported following STROBE guidelines (Supplementary Methods and Supplementary Table S1, available as Supplementary data at *IJE* online).^14^

### Data source

This study used individual patient data from UK Biobank, linked to COVID-19 data sets from laboratories (test results), hospitals (inpatient and critical care admissions) and death certificates. UK Biobank is a population-based prospective study linking individual genetic, biomarker, survey and electronic health record data from >500 000 UK participants, aged 40–69 years at recruitment (2006–2010) when self-report questionnaires and biological measurements were undertaken.^15^

### Study population

The study population included UK Biobank participants who provided baseline assessment data, were alive at the start of the study period and had not withdrawn consent. Participants from assessment centres outside England were excluded, as COVID-19 linked data were unavailable. Study follow-up commenced at the study start date (1 January 2020) and ended at the earliest of the study end date (31 December 2020) or upon death.

COVID-19 diagnosis was defined by having a positive laboratory test result or an ICD-10 code U071 or U072 recorded in hospital or death certificate data. Cases of COVID-19 diagnosed <7 days prior to the end of the study period were excluded, to enable a minimal follow-up period for outcome recording.

The White European subpopulation was defined as those who self-reported ‘White’ ethnicity at baseline and fell within the European cluster based on principal components analysis of genotypic data (Supplementary Methods, available as Supplementary data at *IJE* online).

### Study outcomes

The primary outcome was severe COVID-19, a composite outcome defined as the earliest of hospital or critical care admission within 28 days of COVID-19 diagnosis or death within 100 days following COVID-19 diagnosis. Hospitalizations or critical care admissions reported 1–3 days prior to COVID-19 diagnosis were included, to allow for delays in laboratory testing (i.e. weekends). The secondary outcome was death within 100 days following COVID-19 diagnosis. In a ‘post-hoc’ analysis, a composite of non-fatal severe COVID-19 disease (i.e. the earliest of hospital or critical care admission within 28 days of COVID-19 diagnosis) was evaluated.

Risk factors studied included demographics, immunosuppressant use and co-morbidities, including autoimmune disease, co-morbidity count and PRS. Age was determined at the date of first COVID-19 diagnosis for the COVID-19 sub-cohort and at the study start for the other UK Biobank patients. Demographics were measured or self-reported by participants at their first UK Biobank assessment (sex: female or male; ethnicity: White, Black or other ethnic group; smoking status: never, former or current; Townsend deprivation index^16^ (derived by UK Biobank from self-reported postcode): quintiles; BMI: <18.5, 18.5 to <25, 25 to <30, 30 to <35, 35 to <40 or ≥40 kg/m^2^). Immunosuppressant use was self-reported and defined as oral glucocorticoid, disease-modifying anti-rheumatic drug or other immunosuppressant exposure at enrolment (Supplementary Table S2, available as Supplementary data at *IJE* online). Autoimmune disease and other co-morbidities [CVD, CRD, CKD, diabetes, hypertension, chronic liver disease (CLD) and neurological disease] were defined using self-reported assessment data (Supplementary Table S3, available as Supplementary data at *IJE* online). The count of other co-morbidities was reported as 0, 1 or ≥2.

Details of genotyping, imputation and quality control (QC), including marker-based and sample-based filters, are reported in Supplementary Methods (available as Supplementary data at *IJE* online). First, three PRSs (PRS_e1_, PRS_e2_ and PRS_e3_) were constructed using effect sizes from the European-only COVID-19 vs population susceptibility GWAS, conducted by COVID19-hg (Release 5, excluding UK Biobank samples).^10^ PRS_e1_ was built using the clumping and thresholding approach implemented by PRSice v2.3.3.^17^ PRS_e2_ was built using SNPs previously associated (*P *<* *1 × 10^–5^) with COVID-19 susceptibility/severity phenotypes (Supplementary Table S4, available as Supplementary data at *IJE* online). PRS_e3_ combined PRS_e1_ and PRS_e2_ in a single risk score, removing duplicate loci. PRSs were tested for association with the severe COVID-19 composite, compared with non-severe COVID-19 (positive RT-PCR test result, with no hospitalization or death) in both the White European and trans-ethnic UK Biobank cohorts (ensuring no overlap between the PRS optimization and testing cohorts), using logistic regression to determine which of PRS_e1,_ PRS_e2_ and PRS_e3_ was the best severity predictor (Supplementary Methods, available as Supplementary data at *IJE* online).

This procedure was repeated using effect sizes from the trans-ethnic COVID-19 vs population susceptibility GWAS (COVID19-hg, Release 5) to produce PRS_t1_, PRS_t2_ and PRS_t3_. These PRS were tested for association with severe COVID-19 in the White European and trans-ethnic UK Biobank cohorts to determine the best predictor of COVID-19 severity. We also constructed a previously reported PRS (PRS_d_)^18^ based on COVID19-hg Release 2, for comparative purposes. To investigate PRS association in the UK Biobank trans-ethnic cohort in more detail, a meta-analysis was performed, testing association of the selected PRS with severe COVID-19 in the individual ethnicities that comprised the full UK Biobank trans-ethnic cohort and assessing their contribution to the final result (Supplementary Methods, available as Supplementary data at *IJE* online).

The PRS most strongly associated with severe COVID-19 (from PRS_e1_, PRS_e2_, PRS_e3_, PRS_t1_, PRS_t2_, PRS_t3_ and PRS_d_), as shown by estimated variance, was constructed for all individuals in the UK Biobank cohort who passed genetic QC (*n *=* *450 449 [89.6%]; Supplementary Methods, available as Supplementary data at *IJE* online).

### Statistical analyses

We described characteristics of UK Biobank participants, the COVID-19 sub-cohort and sub-cohorts with study outcomes. The median duration of follow-up (days) for the COVID-19 sub-cohort was calculated from the date of COVID-19 diagnosis.

Time to event (primary and secondary outcomes) was defined as the number of days between the date of COVID-19 diagnosis and the date at which the event occurred. Where participants were hospitalized and/or admitted to critical care either for 1–3 days prior to, or on, the date of COVID-19 diagnosis, the time to event was set to <1 day. Survival analyses were (i) performed on the trans-ethnic UK Biobank COVID-19 cohort and (ii) restricted to the White European subpopulation. Cumulative probabilities of study outcomes up to 100 days were calculated using Kaplan–Meier survival methods, both overall and stratified by demographics, immunosuppressant use, autoimmune disease, co-morbidities, co-morbidity count and PRS quintile (except for co-morbidities reported by fewer than five patients in each category).

We used logistic regression to estimate the risk of the severe COVID-19 composite, and Cox regression to estimate the risk of death within 100 days post-diagnosis. In Cox regression, the proportional hazards assumption was assessed for each variable based on the Schoenfeld residuals.^19^^,^^20^ Age was modelled as a continuous variable in logistic regression and as cubic splines (three knots) in Cox regression (hazard ratios reported for ages 55, 60, 65, 70 and 75 years). The Akaike information criterion informed model selection and how continuous variables were modelled. Risk factors with overall likelihood ratio test *P*-value of <0.1 in age-adjusted models were included in models adjusted for (i) clinico-demographic factors (either using co-morbidities or co-morbidity count) and (ii) clinico-demographic factors (using co-morbidities) and PRS. In PRS-adjusted models, the PRS was modelled as a continuous variable. Hazard ratios and odds ratios are also reported for PRS quintiles, using PRSs constructed for UK Biobank COVID-19 cases in preceding genetic analyses. Patients with missing demographics or PRSs were excluded from the regression analyses.

All analyses were performed using R Version 4.0.4 and Microsoft SQL 2017.

### Pathway analysis

FUMA v1.3.6a software^21^ was used to provide functional annotations of the SNPs in PRS_e2,_ which was used in downstream analyses (Supplementary Methods, available as Supplementary data at *IJE* online).

## Results

**Figure 1:**
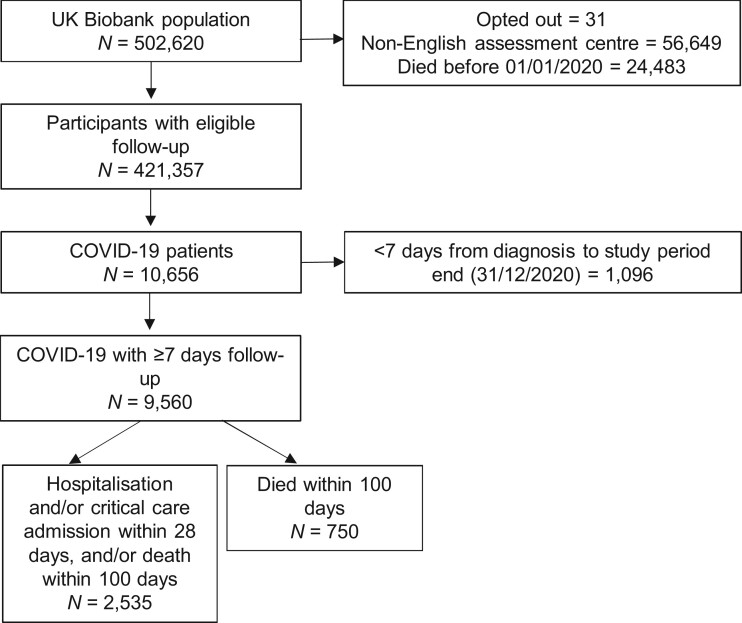
Study STROBE flow diagram of cohort selection. Of the COVID-19 cohort with ≥7 days of follow-up, 166 (1.7%) had missing demographic data. Five (0.5%) of the 1096 who were diagnosed with COVID-19 <7 days before the end of the study period (i.e. diagnosed after 24 December 2020) were reported to have died.

We identified a cohort of 9560 patients with COVID-19 (excluding 1096 patients with <7 days of follow-up), of whom 50.8% (*n *=* *4860) were women and 7274 (76.1%) were White European (Table 1 and Figure 1). The median age at diagnosis was 65 [interquartile range (IQR)  = 58–74] years. The median duration of follow-up was 61 (IQR = 34–88) days. There were 166 (1.7%) patients with missing demographic data and 1107 (13.1%) for whom PRSs could not be calculated. There were 2362 (24.7%) patients who were hospitalized and 750 (7.8%) patients who died within 100 days of their COVID-19 diagnosis date (Supplementary Descriptive Results, available as Supplementary data at *IJE* online).

**Table 1:** Baseline characteristics of eligible UK Biobank participants, those diagnosed with COVID-19 and those with severe COVID-19 or death following COVID-19 diagnosis

	All UK Biobank participants	COVID-19 cohort	Severe COVID-19	Death
	(*N = *502 489)	(*N = *9560)	(*N = *2535)	(*N = *750)
Median age (years) (Q1–Q3)	69 (61–74)	65 (58–74)	73 (66–77)	76 (72–78)
Females (%)	273 375 (54.4)	4860 (50.8)	998 (39.4)	251 (33.5)
Ethnicity (%)	499 712	9507	2508	742
White	472 679 (94.6)	8645 (90.9)	2268 (90.4)	685 (92.3)
Black	8061 (1.6)	253 (2.7)	91 (3.6)	28 (3.8)
Other	18 972 (3.8)	612 (6.4)	149 (5.9)	29 (3.9)
Smoking status (%)	499 540	9496	2500	738
Never	273 513 (54.8)	4903 (51.6)	1044 (41.8)	262 (35.5)
Former	173 050 (34.6)	3506 (36.9)	1094 (43.8)	357 (48.4)
Current	52 977 (10.6)	1087 (11.4)	362 (14.5)	119 (16.1)
Townsend deprivation quintile (%)	501 865	9552	2534	749
1 (least deprived)	100 356 (20.0)	1476 (15.5)	354 (14.0)	110 (14.7)
2	100 384 (20.0)	1691 (17.7)	395 (15.6)	117 (15.6)
3	100 377 (20.0)	1836 (19.2)	427 (16.9)	134 (17.9)
4	100 373 (20.0)	1996 (20.9)	522 (20.6)	147 (19.6)
5 (most deprived)	100 375 (20.0)	2553 (26.7)	836 (33.0)	241 (32.2)
Body mass index [kg/m^2^ (%)]	499 384	9485	2498	735
<18.5	2626 (0.5)	33 (0.04)	12 (0.5)	5 (0.7)
18.5 to <25	16 404 (32.5)	2472 (26.1)	459 (18.4)	139 (18.9)
25 to <30	21 211 (42.5)	3988 (42.1)	1009 (40.4)	292 (39.7)
30 to <35	87 548 (17.5)	2066 (21.8)	641 (25.7)	184 (25.0)
35 to <40	24 992 (5.0)	655 (6.9)	261 (10.4)	80 (10.9)
≥40	9703 (1.9)	271 (2.9)	116 (4.6)	35 (4.8)
Median follow-up duration (days) (Q1–Q3)	*NA*	61 (34–88)	64 (20–234)	11 (4–22)
Immunosuppressant use	12 545 (2.5)	285 (3)	136 (5.4)	43 (5.7)
Autoimmune disease	23 378 (4.7)	459 (4.8)	174 (6.9)	46 (6.1)
CVD (%)	54 860 (10.9)	1.224 (12.8)	569 (22.4)	199 (26.5)
CRD (%)	66 979 (13.3)	1479 (15.5)	471 (18.6)	125 (16.7)
CKD (%)	3583 (0.7)	74 (0.8)	29 (1.1)	8 (1.1)
Diabetes (%)	26 964 (5.4)	679 (7.1)	355 (14.0)	131 (17.5)
Hypertension (%)	138 475 (27.6)	2734 (28.6)	1105 (43.6)	383 (51.1)
CLD (%)	1369 (0.3)	28 (0.3)	16 (0.6)	6 (0.8)
Neurological disease (%)	10 497 (2.1)	222 (2.3)	106 (4.2)	34 (4.5)
Co-morbidity count^a^				
0	279 317 (55.6)	5038 (52.7)	866 (34.2)	207 (27.6)
1	158 420 (31.5)	3029 (31.7)	933 (36.8)	286 (38.1)
≥2	64 752 (12.9)	1493 (15.6)	736 (29.0)	257 (34.3)
PRS_e2_ quintile (%)	450 449	8453	2224	668
1	90 090 (20.0)	1349 (16.0)	325 (14.6)	100 (15.0)
2	90 090 (20.0)	1499 (17.7)	384 (17.3)	123 (18.4)
3	90 090 (20.0)	1634 (19.3)	425 (19.1)	120 (18.0)
4	90 090 (20.0)	1802 (21.3)	476 (21.4)	147 (22.0)
5	90 089 (20.0)	2169 (25.7)	614 (27.6)	178 (26.6)
PRS_e2_ quintile				
White European subpopulation (%)	404 534	7274	1908	590
1	82 490 (20.4)	1195 (16.4)	280 (14.7)	90 (15.3)
2	82 198 (20.3)	1320 (18.1)	335 (17.6)	108 (18.3)
	81 400 (20.1)	1433 (19.7)	373 (19.5)	111 (18.8)
4	80 623 (19.9)	1542 (21.2)	411 (21.5)	123 (20.8)
5	77 823 (19.2)	1784 (24.5)	509 (26.7)	158 (26.8)

Severe COVID-19: hospitalization or critical care admission within 28 days of COVID-19 diagnosis or death within 100 days of COVID-19 diagnosis.

a Count of the following co-morbidities: cardiovascular disease, chronic respiratory disease, chronic kidney disease, diabetes, hypertension, chronic liver disease, neurological disease.

Immunosuppressants are listed in Supplementary Table S2 (available as Supplementary data at *IJE* online).

PRS_e2_ was calculated as described in the Supplementary Methods (available as Supplementary data at *IJE* online), derived using European effect sizes and applied to both European and trans-ethnic UK Biobank populations.

Autoimmune diseases include: rheumatoid arthritis, giant cell arteritis, inflammatory bowel disease, psoriasis, spondyloarthritis, connective tissue disease and vasculitis.

CKD, chronic kidney disease; CLD, chronic liver disease; CRD, chronic respiratory disease; CVD, cardiovascular disease; NA, not applicable; PRS_e2_, White European polygenic risk score 2; Q1, first quartile; Q3, third quartile.

Of the seven PRSs investigated, PRS_e2_ (based on associated SNPs from previous studies and optimized to European data) was the most strongly associated with COVID-19 severity in UK Biobank and explained the greatest estimated variance in both the White European (*P *=* *6.23 × 10^–4^, *R*^2^ = 2.3 × 10^–3^) and trans-ethnic (*P *=* *9.81 × 10^–4^, *R*^2^ = 1.87 × 10^–3^) cohorts (Supplementary Results, Supplementary Table S5, available as Supplementary data at *IJE* online). We conducted a meta-analysis of the association between PRS_e2_ and COVID-19 severity across individual ethnic groups in UK Biobank. The total coefficient for the fixed effects model was 0.08 (*SE *=* *0.03), *P *=* *2.49 × 10^–3^ (Supplementary Results, Supplementary Figure S1 and Supplementary Table S6, available as Supplementary data at *IJE* online). PRS_e2_ was used in all subsequent analyses.

The cumulative probability of severe COVID-19 was 27.1% (95% CI 26.1–28.0%) and estimates for demographics, immunosuppressant use, PRS_e2_ quintile and co-morbidity count are shown in Supplementary Tables S7–S13 and Supplementary Figure S2 (available as Supplementary data at *IJE* online). Cumulative risk was also increased in patients with autoimmune disease and with individual co-morbidities. The results were similar for the cumulative probability of death within 100 days (overall = 9.3%, 95% CI 8.6–10.0%) (Supplementary Figure S3, available as Supplementary data at *IJE* online), although the associations with CRD and CKD had *P *>* *0.05. For the clinico-demographic factors, the trends in the cumulative probability of severe COVID-19 and death were also similar in the White European subpopulation diagnosed with COVID-19 (Supplementary Figures S4 and S5, available as Supplementary data at *IJE* online). In this White European subpopulation, evidence of association with the PRS_e2_ quintile was found for the cumulative probability of severe COVID-19, but not for the cumulative probability of death.

Age was strongly associated with severe COVID-19, even after adjustment for other demographics, immunosuppressant use, individual morbidities (Table 2) and co-morbidity count (Supplementary Table S14, available as Supplementary data at *IJE* online). In clinico-demographic and PRS_e2_-adjusted models, the risk was higher in men [adjusted odds ratio (AOR) 1.70, 95% CI 1.52–1.91 compared with women], in Black ethnicity (AOR 2.21, 95% CI 1.59–3.06 compared with White), in current smokers compared with never smokers (AOR 1.89, 95% CI 1.58–2.25) and among more socio-economically deprived (e.g. the most deprived quintile compared with the least: AOR 1.33, 95% CI 1.11–1.59). Compared with a BMI of 18.5–24.9, the risk was higher with increasing levels of obesity. Risk was also higher in immunosuppressant users (AOR 1.88, 95% CI 1.36–2.60). In clinico-demographic and PRS_e2_-adjusted models, the risk persisted for co-morbidities other than autoimmune disease and CKD, including: CVD (AOR 1.32, 95% CI 1.13–1.53), CRD (AOR 1.27, 95% CI 1.09–1.47), diabetes (AOR 1.62, 95% CI 1.33–1.97), hypertension (AOR 1.23, 95% CI 1.09–1.39), CLD (AOR 3.14, 95% CI 1.31–7.58) and neurological disease (AOR 1.94, 95% CI 1.38–2.71). There was also a positive association with increasing co-morbidity count (e.g. ≥2 compared with 0: AOR 2.03, 95% CI 1.74–2.37) and with increasing PRS_e2_ (e.g. the highest quintile compared with the lowest: AOR 1.32, 95% CI 1.11–1.58. *P *<* *0.001 for the continuous variable). Similar associations were found in the White European subpopulation (Supplementary Table S15, available as Supplementary data at *IJE* online). In both cohorts, the estimated effects of clinico-demographic risk factors showed little change when adjusted for PRS_e2_. In the ‘post-hoc’ analysis of non-fatal severe COVID-19, clinico-demographic and PRS-adjusted odds ratios of hospitalized patients were closely comparable, irrespective of their final outcome, with overlapping confidence intervals (Supplementary Table S16, available as Supplementary data at *IJE* online).

**Table 2:** Age-adjusted, clinico-demographic-adjusted and clinico-demographic and polygenic risk score (PRS)-adjusted odds ratios of severe COVID-19 in patients diagnosed with COVID-19 (*N = *8325)

Risk factor	Age-adjusted		Clinico-demographic-adjusted		Clinico-demographic and PRS_e2_-adjusted	
	OR (95% CI)	*P*-value	OR (95% CI)	*P*-value	OR (95% CI)	*P*-value
Age (continuous)	*NA*		1.11 (1.10–1.11)	<0.001	1.11 (1.10–1.11)	<0.001
Sex						
Female	1	<0.001	1	<0.001	1	
Male	1.75 (1.58–1.95)		1.70 (1.52–1.90)		1.70 (1.52–1.91)	<0.001
Ethnicity						
White	1	<0.001	1	<0.001	1	<0.001
Black	2.67 (1.95–3.62)		2.12 (1.53–2.93)		2.21 (1.59–3.06)	
Other	1.38 (1.10–1.73)		1.16 (0.91–1.47)		1.10 (0.86–1.39)	
Smoking status						
Never	1	<0.001	1	<0.001	1	<0.001
Former	1.25 (1.11–1.40)		1.12 (0.99–1.27)		1.12 (0.99–1.26)	
Current	2.04 (1.73–2.41)		1.90 (1.59–2.26)		1.89 (1.58–2.25)	
Townsend deprivation quintile						
1 (least deprived)	1		1		1	<0.001
2	0.92 (0.76–1.11)	<0.001	0.91 (0.75–1.11)	<0.001	0.91 (0.75–1.11)	
3	0.92 (0.76–1.11)		0.86 (0.71–1.05)		0.86 (0.71–1.05)	
4	1.23 (1.03–1.47)		1.10 (0.91–1.33)		1.10 (0.91–1.32)	
5 (most deprived)	1.69 (1.43–2.00)		1.33 (1.11–1.59)		1.33 (1.11–1.59)	
BMI (kg/m^2^)						
<18.5	2.88 (1.23–6.46)	<0.001	2.39 (0.98–5.56)	<0.001	2.33 (0.96–5.46)	<0.001
18.5 to <25	1		1		1	
25 to <30	1.29 (1.12–1.49)		1.13 (0.98–1.31)		1.13 (0.97–1.31)	
30 to <35	1.71 (1.46–2.00)		1.41 (1.19–1.66)		1.41 (1.19–1.66)	
35 to <40	2.69 (2.17–3.34)		2.10 (1.67–2.64)		2.11 (1.68–2.65)	
≥40	3.49 (2.57–4.73)		2.52 (1.81–3.48)		2.51 (1.81–3.48)	
Immunosuppressant use	2.16 (1.64–2.84)	<0.001	1.89 (1.37–2.61)	<0.001	1.88 (1.36–2.60)	<0.001
Autoimmune disease	1.54 (1.23–1.93)	<0.001	1.23 (0.94–1.60)	0.139	1.22 (0.93–1.59)	0.148
CVD	1.75 (1.52–2.02)	<0.001	1.32 (1.13–1.53)	<0.001	1.32 (1.13–1.53)	<0.001
CRD	1.36 (1.18–1.57)	<0.001	1.26 (1.09–1.46)	0.002	1.27 (1.09–1.47)	0.002
CKD	2.10 (1.22–3.58)	0.008	1.50 (0.84–2.65)	0.17	1.49 (0.83–2.64)	0.176
Diabetes	2.49 (2.07–2.99)	<0.001	1.61 (1.33–1.97)	<0.001	1.62 (1.33–1.97)	<0.001
Hypertension	1.62 (1.45–1.81)	<0.001	1.23 (1.09–1.39)	<0.001	1.23 (1.09–1.39)	<0.001
CLD	4.67 (1.58–8.59)	0.003	3.18 (1.34–7.69)	0.009	3.14 (1.31–7.58)	0.01
Neurological disease	1.91 (1.38–2.63)	<0.001	1.23 (0.94–1.6)	<0.001	1.94 (1.38–2.71)	<0.001
Co-morbidity count^a^						
0	1	<0.001	1	<0.001	1	<0.001
1	1.52 (1.34–1.72)		1.35 (1.19–1.53)		1.35 (1.19–1.53)	
≥2	2.73 (2.36–3.16)		2.03 (1.74–2.37)		2.03 (1.74–2.37)	
PRS_e2_ quintile						
1	1	0.006	1	0.011	*NA*	
2	1.14 (0.95–1.37)		1.12 (0.93–1.36)			
3	1.11 (0.93–1.33)		1.08 (0.89–1.30)			
4	1.24 (1.04–1.48)		1.25 (1.04–1.50)			
5	1.35 (1.14–1.60)		1.32 (1.11–1.58)			

Severe COVID-19: hospitalization or critical care admission within 28 days of COVID-19 diagnosis or death within 100 days of COVID-19 diagnosis.

Clinico-demographic-adjusted model included age (as continuous), sex, ethnicity, smoking status, Townsend deprivation quintile, body mass index, immunosuppressant use, autoimmune disease and co-morbidities. In this model, co-morbidity count is adjusted for these variables excepting co-morbidities.

Clinico-demographic and PRS_e2_-adjusted model included PRS_e2_ (as continuous) in addition to the variables included in the clinico-demographic-adjusted model (listed above). In this model, PRS_e2_ (as continuous) had a significant *P*-value (<0.001). In this model, co-morbidity count is adjusted for PRS_e2_ (as continuous) in addition to the variables included when modelling co-morbidity count in the clinico-demographic-adjusted model (listed above).

a Count of the following co-morbidities: cardiovascular disease, chronic respiratory disease, chronic kidney disease, diabetes, hypertension, chronic liver disease, neurological disease.

*P*-value from the overall likelihood ratio test for association.

BMI, body mass index; CKD, chronic kidney disease; CLD, chronic liver disease; CRD, chronic respiratory disease; CVD, cardiovascular disease; NA, not applicable; OR, odds ratio; PRS_e2_, White European polygenic risk score 2.

Age was associated with risk of death within 100 days following COVID-19, even in adjusted models (Table 3 and Supplementary Table S17, available as Supplementary data at *IJE* online). In clinico-demographic and PRS_e2_-adjusted models, risk was higher in men than in women [adjusted hazard ratio (AHR) 1.69, 95% CI 1.43–2.01], in Black than in White ethnicity (AHR 2.31, 95% CI 1.51–3.53), in former or current smokers (AHR 1.20, 95% CI 1.01–1.43; AHR 1.79 95% CI 1.41–2.28, respectively) than in never smokers. The clinico-demographic and PRS_e2_-adjusted risk increased with increasing levels of obesity compared with a BMI of 18.5–24.9 and was higher in immunosuppressant users (AHR 1.51, 95% CI 1.08–2.11). A positive association was found for CVD in the age-adjusted model (AHR 1.34, 95% CI 1.12–1.60) but not the clinic-demographic or clinic-demographic and PRS_e2_-adjusted models. The clinico-demographic and PRS_e2_-adjusted models showed associations for diabetes (AHR 1.40, 95% CI 1.12–1.75) and hypertension (AHR 1.30, 95% CI 1.10–1.53) but not for autoimmune disease or other co-morbidities. There was also a positive association with increasing co-morbidity count (e.g. ≥2 compared with 0: AHR 1.68, 95% CI 1.36–2.08). Similar associations were found in the White European subpopulation, except for socio-economic deprivation, BMI and immunosuppressant use (Supplementary Table S18, available as Supplementary data at *IJE* online). In both cohorts, the estimated effects of clinico-demographic risk factors showed little change when adjusted for PRS_e2_.

**Table 3:** Age-adjusted, clinico-demographic-adjusted and clinico-demographic and polygenic risk score (PRS)-adjusted hazard ratios of death in patients diagnosed with COVID-19 (*N = *8325)

Risk factor	Age-adjusted		Clinico-demographic-adjusted		Clinico-demographic and PRS_e2_-adjusted	
	HR (95% CI)	*P*-value	HR (95% CI)	*P*-value	HR (95% CI)	*P*-value
Age in years (cubic spline)						
55	*NA*		1	<0.001	1	<0.001
60			2.55 (1.96–3.31)		2.55 (1.96–3.32)	
65			5.93 (3.80–9.26)		5.95 (3.81–9.29)	
70			12.02 (7.25–19.93)		12.06 (7.27–20.02)	
75			22.01 (13.46–35.97)		22.13 (13.53–36.19)	
Sex						
Female	1	<0.001	1	<0.001	1	<0.001
Male	1.80 (1.53–2.12)		1.69 (1.42–2.01)		1.69 (1.43–2.01)	
Ethnicity						
White	1	<0.001	1	0.003	1	0.002
Black	2.81 (1.86–4.23)		2.21 (1.45–3.38)		2.31 (1.51–3.53)	
Other	1.02 (0.68–1.54)		0.90 (0.59–1.37)		0.87 (0.57–1.33)	
Smoking status						
Never	1	<0.001	1	<0.001	1	<0.001
Former	1.33 (1.12–1.57)		1.20 (1.01–1.43)		1.20 (1.01–1.43)	
Current	2.02 (1.60–2.55)		1.81 (1.42–2.30)		1.79 (1.41–2.28)	
Townsend deprivation quintile						
1 (least deprived)	1	0.001	1	0.097	1	0.093
2	0.79 (0.60–1.04)		0.77 (0.58–1.02)		0.77 (0.58–1.02)	
3	0.87 (0.66–1.13)		0.82 (0.62–1.07)		0.82 (0.62–1.07)	
4	1.02 (0.79–1.32)		0.95 (0.73–1.24)		0.95 (0.73–1.23)	
5 (most deprived)	1.25 (0.98–1.58)		1.03 (0.81–1.32)		1.03 (0.81–1.32)	
BMI (kg/m^2^)						
<18.5	2.66 (0.98–7.19)	<0.001	2.53 (0.93–6.87)	0.004	2.51 (0.92–6.82)	0.004
18.5 to <25	1		1		1	
25 to <30	1.01 (0.81–1.25)		0.89 (0.72–1.11)		0.89 (0.72–1.11)	
30 to <35	1.21 (0.96–1.53)		1.00 (0.79–1.27)		1.00 (0.79–1.28)	
35 to <40	1.75 (1.31–2.35)		1.41 (1.04–1.92)		1.42 (1.05–1.93)	
≥40	1.98 (1.33–2.93)		1.52 (1.01–2.29)		1.51 (1.00–2.27)	
Immunosuppressant use	1.43 (1.02–1.99)	0.046	1.51 (1.08–2.11)	0.023	1.51 (1.08–2.11)	0.023
Autoimmune disease	1.10 (0.80–1.51)	0.547	*NA*		*NA*	
CVD	1.34 (1.12–1.60)	0.002	1.10 (0.92–1.32)	0.309	1.10 (0.91–1.32)	0.325
CRD	1.03 (0.84–1.27)	0.749	*NA*		*NA*	
CKD	1.26 (0.60–2.65)	0.558	*NA*		*NA*	
Diabetes	1.87 (1.52–2.30)	<0.001	1.39 (1.12–1.74)	0.004	1.40 (1.12–1.75)	0.004
Hypertension	1.53 (1.31–1.79)	<0.001	1.30 (1.10–1.53)	0.002	1.30 (1.10–1.53)	0.002
CLD	2.50 (0.94–6.69)	0.112	*NA*		*NA*	
Neurological disease	1.38 (0.94–2.01)	0.115	*NA*		*NA*	
Co-morbidity count^a^						
0	1	<0.001	1	<0.001	1	<0.001
1	1.48 (1.22–1.81)		1.36 (1.11–1.65)		1.35 (1.11–1.65)	
≥2	2.06 (1.68–2.53)		1.68 (1.36–2.08)		1.68 (1.36–2.08)	
PRS_e2_ quintile						
1	1	0.398	1	0.354	*NA*	
2	1.16 (0.88–1.51)		1.15 (0.88–1.51)			
3	1.00 (0.76–1.31)		1.00 (0.76–1.31)			
4	1.19 (0.92–1.54)		1.21 (0.93–1.57)			
5	1.18 (0.92–1.52)		1.20 (0.93–1.54)			

Clinico-demographic-adjusted model included age (cubic spline with three knots), sex, ethnicity, smoking status, Townsend deprivation index quintile, body mass index, immunosuppressant use, CVD, diabetes and hypertension. In this model, co-morbidity count is adjusted for these variables excepting CVD, diabetes and hypertension.

Clinico-demographic and PRS_e2_-adjusted model included PRS_e2_ (as continuous) in addition to the variables included in the clinico-demographic-adjusted model (listed above). *P*-value for PRS_e2_ (as continuous) was 0.067. In this model, co-morbidity count is adjusted for PRS_e2_ (as continuous) in addition to the variables included when modelling co-morbidity count in the clinico-demographic-adjusted model (listed above).

a Count of the following co-morbidities: cardiovascular disease, chronic respiratory disease, chronic kidney disease, diabetes, hypertension, chronic liver disease, neurological disease.

*P*-value from the overall likelihood ratio test for association.

BMI, body mass index; CKD, chronic kidney disease; CLD, chronic liver disease; CRD, chronic respiratory disease; CVD, cardiovascular disease; HR, hazard ratio; NA, not applicable; PRS_e2_, White European polygenic risk score 2.

### Pathway analyses

Genetic markers of PRS_e2_ were physically mapped (within 10 kb) to 134 genes by Ensembl VEP and an additional 23 genes by FUMA, including the previously reported *OAS1*–*OAS3* gene cluster (Supplementary Table S19, available as Supplementary data at *IJE* online).^11^ Furthermore, the MAGMA gene-based test (executed in FUMA) found six genes that were enriched with SNPs from PRS_e2_ at the Bonferroni-corrected significance level (*P *<* *2.6 × 10^–6^; Supplementary Methods, available as Supplementary data at *IJE* online). These genes included *LZTFL1*, *OAS1*, *OAS3*, *FYCO1*, *XCR1* and *SLC6A20* (Supplementary Figure S6, available as Supplementary data at *IJE* online). Finally, several pathways in the Reactome database were enriched for SNPs in PRS_e2_ (Supplementary Table S20, available as Supplementary data at *IJE* online). These pathways included the ‘OAS antiviral response’ pathway (adjusted-*P *=* *5.84 × 10^–4^), ‘interferon alpha–beta signalling’ pathway (adjusted-*P *=* *2.84 × 10^–3^), ‘interleukin 10 signalling’ pathway (adjusted-*P *=* *2.87 × 10^–2^) and the ‘chemokine receptors bind chemokines’ gene set (adjusted-*P *=* *2.87 × 10^–2^).

## Discussion

This observational study was the first to investigate the association of clinico-demographic and genetic risk factors with severe COVID-19 and death in patients diagnosed with COVID-19. In this pre-COVID-19 cohort of 502 489 individuals we have, to our knowledge, derived and optimized the best-performing, publicly available PRS for the prediction of severe COVID-19 in both White European and trans-ethnic populations, which is independent of known clinico-demographic factors. PRS_e2_ consisted of 133 SNPs, was associated with severe COVID-19 in both populations and was enriched for SNPs in antiviral and immune-response pathways.

In this pre-SARS-CoV2 vaccination cohort, the risk of severe COVID-19 was associated with PRS_e2_ even after adjustment for clinico-demographic factors, suggesting it captures a different component of COVID-19 severity risk. Based on effect sizes, the magnitude of risk associated with the highest PRS_e2_ quintile for severe COVID-19 was equivalent to that afforded by well-known risk factors, such as living in the most deprived areas and having CVD.

The increased risk of severe COVID-19 with age, male sex, Black ethnicity, high BMI, CVD, CRD, diabetes, hypertension, CLD and neurological disease corroborates other studies.^3^^,^^22^ We also found increased risk of severe COVID-19 from being a former or current smoker, immunosuppressant use and higher co-morbidity count. We examined the association of BMI with severe COVID-19 and death with more granularity than previous studies,^4^^,^^23^ showing the increasing risk associated with each additional 5 kg/m² of BMI between 25 and 40 kg/m².

The increased risk of death with age, male sex, Black ethnicity, former or current smoking, high BMI or having CVD, diabetes or hypertension corroborates the findings of an earlier UK Biobank study.^24^ We also found increased risk of death with socio-economic deprivation, immunosuppressant use and higher co-morbidity count. The smaller sample size limited the power to detect associations with risk of death for less prevalent co-morbidities, but trends towards increased risk were broadly comparable to those observed with severe COVID-19. To date, the relative safety of different immunosuppressants in the setting of a global pandemic has not been considered in health economic analyses or treatment decisions.

PRS_e2_ performed well in both a European and trans-ethnic severe COVID-19 cohort, although its association with individual ethnicities demonstrated some variation in effect size, likely due to smaller sample sizes and differences in LD structure between ethnic populations. This work attests to the validity of combining GWA evidence from multiple studies and related traits (e.g. COVID-19 severity, susceptibility) to collectively predict genetic risk and illustrates the value of investigating SNP/trait associations using a more liberal threshold in summary statistics (in this case, *P *<* *1 × 10^–5^), highlighting the utility of PRSs in summarizing these variants.

Our bioinformatic analyses highlighted several biological pathways enriched for SNPs in PRS_e2_, including the Reactome ‘OAS antiviral response’ pathway responsible for the mediation of antiviral innate immunity through the regulation of ribonucleic acid (RNA) degradation.^25^ Furthermore, SNPs of the *OAS1–OAS3* gene cluster, *IFNAR2* and *AP000295*.*9* were also enriched in the ‘interferon alpha–beta signalling’ pathway, which involves the induction of type I interferons in virus-infected cells, representing another stage in the human antiviral response.^26^ This suggests host genetic variation in antiviral immune pathways may contribute to the disparity in disease severity amongst individuals. Given the positive results of recent clinical trials for antiviral drug molnupiravir,^27^ such immune pathways may be of interest for further investigation and therapeutic targeting in severe COVID-19. ‘Interleukin-10 signalling’ pathway variants in *IL10RB*, *CCR2* and *CCR5* were also enriched in PRS_e2_ and may contribute to the development of severe COVID-19. Interleukin-10 (IL-10) is an anti-inflammatory cytokine and signalling of IL-10 is known to be important in limiting host immune response to pathogens.^28^

### Strengths and limitations

UK Biobank recruited older participants, which enabled this study to specifically examine risk in older patients, who are most at risk of COVID-19 infection and severe outcomes. The study time frame covered the pre-COVID-19 vaccination era, to avoid potential bias from the prioritized vaccine rollout programme. It incorporated deaths up to the end of December 2020, thereby extending the findings of a previous UK Biobank study of risk of death up to the end of September, covering the second wave of the pandemic.^24^ We conducted multivariable regression analyses to investigate the relationship between covariates, highlighting the important contribution of PRSs, beyond that associated with clinico-demographic factors. The analyses were performed in a White European subpopulation to ensure that the European effect sizes used to defining PRS_e2_ did not introduce bias in how it performed between ethnicities: results from the subpopulation were consistent with the main analysis.

Study limitations included a modest number of cases of COVID-19 (*n* = 9560) when compared with national cohorts. Further, UK Biobank participants may not be representative of the UK population, though the investigated assessment of risk factors does not require a representative population.^29^ The clinico-demographic characteristics of the cohort were collected at the time of the baseline assessment (∼10 years earlier) and self-reported. Given the study period and sample size, we could not investigate temporal or seasonal patterns in the association between risk factors and the outcomes. The use of European effect sizes in the construction of PRS_e2_ may have biased the PRS to perform more effectively in Europeans than other subpopulations. Further improvements in trans-ethnic PRS performance can be expected as more data are generated from non-European populations, including ethnic-specific PRSs.

## Conclusion

Using a trans-ethnic cohort of >500 000 UK Biobank participants in the pre-COVID-19 vaccination era, we derived an optimized PRS that associated with risk of severe COVID-19 and death in COVID-19 patients, even after adjustment for well-known clinico-demographic risk factors. We highlighted the risk associated with co-morbidities, immunosuppressant use and obesity. This study emphasizes the novel contribution to be gained from using genomic data alongside phenotyping in developing understanding of COVID-19 and its management.

## Ethics approval

The study was approved by UK Biobank (project 24559). UK Biobank has ethical approval from the National Research Ethics Committee (REC reference 11/NW/0382) and obtained informed electronic consent from all participants. There was no patient–public involvement in the study, which used non-identifiable data.

## Data availability

UK Biobank data were provided under a licence that does not permit sharing. The code-lists used in definitions and the derived results are published in the manuscript and supporting material.

## Supplementary data

Supplementary data are available at *IJE* online.

## Author contributions

Study conception and design: A.W.M., M.P.R. and M.I.; analysis planning, data collection, verification and data analysis: S.S.R.C. and N.J.M.C.; all authors contributed to data interpretation, drafting and critical revision of the article and approved the final submitted version.

## Funding

This work was funded by a Medical Research Council Confidence in Concept award (grant number MC_PC_19042) and was additionally supported a National Institute for Health Research (NIHR) Senior Investigator award to A.W.M. and by the NIHR Leeds Biomedical Research Centre and Diagnostic Evaluation Co-operative. The views expressed are those of the authors and not necessarily those of the NIHR or the Department of Health and Social Care. The funders had no role in the study design; in the collection, analysis and interpretation of data; in the writing of the report; and in the decision to submit the paper for publication.

## Supplementary Material

dyac137_Supplementary_Data
