# Supplementary material for: Interplay between demographic, clinical and polygenic risk factors for severe COVID-19
Source: Int J Epidemiol. 2022 Jun 30;51(5):1384–95. doi: 10.1093/ije/dyac137 (PMC9278202; doi:10.1093/ije/dyac137)
Supplement: dyac137_Supplementary_Data [file dyac137_supplementary_data.docx]

Supplementary Material

**Table of Contents**

[1. Supplementary Methods 3](#_Toc104540411)

[Ethics and reporting 3](#_Toc104540412)

Supplementary [Table S1. STROBE Checklist for Cohort Studies, with page number reference to this study 3](#_Toc104540413)

[Data preparation 4](#_Toc104540414)

Supplementary [Table S2. Drug terms used to determine immunosuppressant medication 4](#_Toc104540415)

Supplementary [Table S3. Self-reported diseases^ used to define morbidities 4](#_Toc104540416)

[Polygenic Risk Score Construction and Optimisation 5](#_Toc104540417)

[UK Biobank genotyping and quality control 5](#_Toc104540418)

[Polygenic Risk Score Construction 5](#_Toc104540419)

[Polygenic Risk Scores using European Effect Sizes 6](#_Toc104540420)

Supplementary [Table S4. Summary of studies providing genetic associations with COVID-19 phenotypes 6](#_Toc104540421)

[Polygenic Risk Scores using Transethnic Effect Sizes 7](#_Toc104540422)

[Comparison to a Published Polygenic Risk Score 8](#_Toc104540423)

[Ethnicity Meta-Analysis 8](#_Toc104540424)

[Pathway Analyses 8](#_Toc104540425)

[2. Supplementary Results 8](#_Toc104540426)

[Polygenic Risk Score Results 8](#_Toc104540427)

Supplementary [Table S5. Results of PRS tested in UK Biobank COVID-19 severity cohorts, using effect sizes from either European (PRSe1, PRSe2, PRSe3) or transethnic (PRSt1, PRSt2, PRSt3) COVID-19 susceptibility GWAS data from the COVID19-hg consortium. 9](#_Toc104540428)

[Polygenic Risk Scores using European Effect Sizes 9](#_Toc104540429)

[Polygenic Risk Scores using Transethnic Effect Sizes 9](#_Toc104540430)

[Best-Fit Polygenic Risk Score 10](#_Toc104540431)

[Dite et al. (2021) Polygenic Risk Score 10](#_Toc104540432)

[Ethnicity meta-analysis 10](#_Toc104540433)

Supplementary [Figure S1. Forest plot summarizing results of the inverse-variance weighted, fixed effects meta-analyses of PRS_e2_, performed across multiple ethnic populations, including heterogeneity *X*^2^ and *I*^2^ statistics. 10](#_Toc104540434)

Supplementary [Table S6. Results of the meta-analysis examining the association between PRS_e2_ and the transethnic UK Biobank severe COVID-19 case-control cohort. 11](#_Toc104540435)

[Descriptive results 11](#_Toc104540436)

[Survival analysis results 11](#_Toc104540437)

Supplementary [Table S7. Kaplan Meier probability estimates of severe COVID-19 and death in patients diagnosed with COVID-19, by sex 11](#_Toc104540438)

Supplementary [Table S8. Kaplan Meier probability estimates of severe COVID-19 and death in patients diagnosed with COVID-19, per ethnicity (*N=*9,507) 12](#_Toc104540439)

Supplementary [Table S9. Kaplan Meier probability estimates of severe COVID-19 and death in patients diagnosed with COVID-19, per smoking status 12](#_Toc104540440)

Supplementary [Table S10. Kaplan Meier probability estimates of severe COVID-19 and death in patients diagnosed with COVID-19, per Townsend deprivation quintile 13](#_Toc104540441)

Supplementary [Table S11. Kaplan Meier probability estimates of severe COVID-19 and death in patients diagnosed with COVID-19, per BMI group 15](#_Toc104540442)

Supplementary [Table S12. Kaplan Meier probability estimates of severe COVID-19 (hospitalisation, critical care admission or death) and death in patients diagnosed with COVID-19, per reported immunosuppressant use status 16](#_Toc104540443)

Supplementary [Table S13. Kaplan Meier probability estimates of severe COVID-19 and death in patients diagnosed with COVID-19, per White European polygenic risk score 2 quintile 17](#_Toc104540444)

Supplementary [Table S14. Unadjusted and adjusted (demographics, immunosuppressant use, autoimmune disease and comorbidity count) odds ratios of severe COVID-19 (hospitalisation, critical care admission or death) in patients diagnosed with COVID-19 (*N=*8,325) 18](#_Toc104540445)

Supplementary [Table S15. Unadjusted, age-adjusted, clinico-demographic adjusted and clinico-demographic and PRSe_2_ adjusted odds ratios of severe COVID-19 (hospitalisation, critical care admission or death) in the White European subpopulation diagnosed with COVID-19 (*N=*7,206) 20](#_Toc104540446)

Supplementary [Table S17. Unadjusted and adjusted (demographics, immunosuppressant use, autoimmune disease and comorbidity count) hazard ratios of death in patients diagnosed with COVID-19 (*N=*8,325) 23](#_Toc104540447)

Supplementary [Table S18. Unadjusted, age-adjusted, clinico-demographic adjusted and clinico-demographic and PRS_e2_ adjusted hazard ratios of death in the White European subpopulation diagnosed with COVID-19 (*N=* 7,206) 24](#_Toc104540448)

Supplementary [Table S19: Information about SNPs in PRS_e2_. 27](#_Toc104540449)

Supplementary [Table S20: Results of gene-set enrichment analysis for genes encoded by SNPs of PRS_e2_. Conducted by FUMA v1.3.6a using Reactome database. 35](#_Toc104540450)

Supplementary [Figure S2. Kaplan Meier curves of severe COVID-19 (hospitalisation, critical admission or death) in patients diagnosed with COVID-19, per demographics, immunosuppressant use status, comorbidity count and White European polygenic risk score 2 quintile (*N=*9,560) 36](#_Toc104540451)

Supplementary [Figure S3. Kaplan Meier curves of death in patients diagnosed with COVID-19, per demographics, immunosuppressant use status, comorbidity count and White European polygenic risk score 2 quintile (*N=*9,560) 37](#_Toc104540452)

Supplementary [Figure S4. Kaplan Meier curve of hospitalisation, critical admission or death in the White European subpopulation diagnosed with COVID-19, per demographics, White European polygenic risk score 2 quintile and immunosuppressant use status (*N=*7,274) 38](#_Toc104540453)

Supplementary [Figure S5. Kaplan Meier curve of death in the White European subpopulation diagnosed with COVID-19, per demographic factor, immunosuppressant use status and White European polygenic risk score 2quintile (*N=*7,274) 39](#_Toc104540454)

Supplementary [Figure S6. Manhattan plot for the MAGMA (de Leeuw 2016) gene-based test. Red dotted line represents the –log_10_ genome-wide significance value for 0.05/19,254 = 2.60 x 10^-6^. Enriched genes which pass this level of significance are labelled. 40](#_Toc104540455)

[3. References 40](#_Toc104540456)

## Supplementary Methods

## Ethics and reporting

The study was approved by UK Biobank (project 24559). UK Biobank has ethical approval from the National Research Ethics Committee (REC reference 11/NW/0382), and obtained informed electronic consent from all participants. There was no patient-public involvement in the study, which used non-identifiable data.

Supplementary Table S1. STROBE Checklist for Cohort Studies, with page number reference to this study

|  | **Item No** | **Recommendation** | **Page No** |
| --- | --- | --- | --- |
| Title and abstract | 1 | (*a*) Indicate the study’s design with a commonly used term in the title or the abstract | 2 |
|  |  | (*b*) Provide in the abstract an informative and balanced summary of what was done and what was found | 2 |
| Introduction | | |  |
| Background/rationale | 2 | Explain the scientific background and rationale for the investigation being reported | 5 |
| Objectives | 3 | State specific objectives, including any prespecified hypotheses | 6 |
| Methods | | |  |
| Study design | 4 | Present key elements of study design early in the paper | 6 |
| Setting | 5 | Describe the setting, locations, and relevant dates, including periods of recruitment, exposure, follow-up, and data collection | 6, 7 |
| Participants | 6 | (*a*) Give the eligibility criteria, and the sources and methods of selection of participants. Describe methods of follow-up | 6, 7 |
|  |  | (*b*) For matched studies, give matching criteria and number of exposed and unexposed | NA |
| Variables | 7 | Clearly define all outcomes, exposures, predictors, potential confounders, and effect modifiers. Give diagnostic criteria, if applicable | 7, 8 |
| Data sources/ measurement | 8* | For each variable of interest, give sources of data and details of methods of assessment (measurement). Describe comparability of assessment methods if there is more than one group | 6-8 |
| Bias | 9 | Describe any efforts to address potential sources of bias | 7, 8 |
| Study size | 10 | Explain how the study size was arrived at | 6, 7 |
| Quantitative variables | 11 | Explain how quantitative variables were handled in the analyses. If applicable, describe which groupings were chosen and why | 7-9 |
| Statistical methods | 12 | (*a*) Describe all statistical methods, including those used to control for confounding | 9 |
|  |  | (*b*) Describe any methods used to examine subgroups and interactions | 9 |
|  |  | (*c*) Explain how missing data were addressed | 10 |
|  |  | (*d*) If applicable, explain how loss to follow-up was addressed | NA |
|  |  | (*e*) Describe any sensitivity analyses | NA |
| Results | | |  |
| Participants | 13* | (a) Report numbers of individuals at each stage of study—e.g. numbers potentially eligible, examined for eligibility, confirmed eligible, included in the study, completing follow-up, and analysed | 10, Figure 1 |
|  |  | (b) Give reasons for non-participation at each stage | Figure 1 |
|  |  | (c) Consider use of a flow diagram | Figure 1 |
| Descriptive data | 14* | (a) Give characteristics of study participants (e.g. demographic, clinical, social) and information on exposures and potential confounders | 10 |
|  |  | (b) Indicate number of participants with missing data for each variable of interest | 10 |
|  |  | (c) Summarise follow-up time (e.g., average and total amount) | 10 |
| Outcome data | 15* | Report numbers of outcome events or summary measures over time | 10, 11 |
| Main results | 16 | (*a*) Give unadjusted estimates and, if applicable, confounder-adjusted estimates and their precision (e.g., 95% confidence interval). Make clear which confounders were adjusted for and why they were included | 11, 12 |
|  |  | (*b*) Report category boundaries when continuous variables were categorized | 7 |
|  |  | (*c*) If relevant, consider translating estimates of relative risk into absolute risk for a meaningful time period | NA |
| Other analyses | 17 | Report other analyses done—e.g. analyses of subgroups and interactions, and sensitivity analyses | 10-13 |
| Discussion | | |  |
| Key results | 18 | Summarise key results with reference to study objectives | 13 |
| Limitations | 19 | Discuss limitations of the study, taking into account sources of potential bias or imprecision. Discuss both direction and magnitude of any potential bias | 15, 16 |
| Interpretation | 20 | Give a cautious overall interpretation of results considering objectives, limitations, multiplicity of analyses, results from similar studies, and other relevant evidence | 13, 14 |
| Generalisability | 21 | Discuss the generalisability (external validity) of the study results | 15 |
| Other information | | |  |
| Funding | 22 | Give the source of funding and the role of the funders for the present study and, if applicable, for the original study on which the present article is based | 18 |

## Data preparation

Supplementary Table S2. Drug terms used to determine immunosuppressant medication

| **UK Biobank self-reported medication** |
| --- |
| **Oral glucocorticoid**  (<5/<5) Betnelan; (<5/52) Betnesol; (<5/5) Deflazacort; (<5/137) Dexamethasone; (<5/214) Fludrocortisone; (36/1354) Hydrocortisone; (<5/9) Hydrocortone; (<5/23) Methylprednisolone; (<5/74) Novolizer budesonide; (<5/29) Prednesol; (75/3365) Prednisolone; (6/352) Prednisone |
| **Disease modifying anti-rheumatic drug**  (<5/59) Adalimumab; (<5/<5) Anakinra; (25/1118) Azathioprine; (<5/41) Cellcept; (<5/75) Ciclosporin; (<5/114) Cyclosporin; (<5/<5) Efalizumab; (<5/144) Humira; (<5/22) Imuran; (7/247) Leflunomide / Arava; (<5/<5) Maxtrex; (66/2908) Methotrexate; (<5/252) Mycophenolate; (<5/7) Mycophenolic acid; (<5/13) Myfortic; (/81) Neoral; (<5/25) Prograf; (<5/5) Rapamune; (<5/11) Sirolimus; (<5/229) Tacrolimus; (<5/18) Tacrolimus monohydrate |
| **Other immunosuppressant**  (15/926) Asacol; (<5/<5) Auranofin; (<5/142) Balsalazide disodium; (<5/40) Chloroquine; (<5/67) Colazide; (6/268) Colchicine; (<5/61) Dapsone; (22/849) Hydroxychloroquine; (13/753) Mesalazine; (<5/31) Mesren; (<5/20) Myocrisin; (<5/59) Olsalazine; (<5/22) Penicillamine; (<5/220) Plaquenil; (7/293) Salazopyrin; (<5/7) Salazosulfapyridine; (24/99) Sulfasalazine; (12/289) Sulphasalazine |

Note: Drug terms reported as they appear in UK Biobank, minus any reference to dosage.

For each drug term, the number of patients diagnosed with COVID-19 and number of participants in UK Biobank are reported in brackets.

Supplementary Table S3. Self-reported diseases^ used to define morbidities

| **Morbidity** | **UK Biobank self-reported illness** |
| --- | --- |
| Autoimmune disease | Ankylosing spondylitis (30/1428); antiphospholipid syndrome (<5/39); colitis / not Crohns or ulcerative colitis (32/1403); connective tissue disorder (<5/203); Crohns disease (26/1523); dermatomyositis (<5/39); dermatopolymyositis (<5/7); giant cell / temporal arteritis (<5/116); glomerulnephritis (<5/84); IgA nephropathy (<5/33); inflammatory bowel disease (<5/199); microscopic polyarteritis (<5/10); myositis / myopathy (6/202); polyartertis nodosa (<5/31); polymyalgia rheumatica (20/1236); polymyositis (<5/71); psoriasis (143/6116); psoriatic arthropathy (23/979); rheumatoid arthritis (103/5975); sarcoidosis (11/1064); scleroderma / systemic sclerosis (5/178); Sjogren's syndrome / sicca syndrome (10/514); systemic lupus erythematosus / SLE (13/665); ulcerative colitis (52/2718);vasculitis (6/214); Wegners granulmatosis (<5/100) |
| Cardiovascular disease | Angina (406/16751); aortic stenosis (<5/135); aortic valve disease (<5/68); arterial embolism (5/79); atrial fibrillation (85/4445); brain haemorrhage (5/206); cardiomyopathy (6/429); deep venous thrombosis (DVT) (249/10330); heart / cardiac problem (50/1806); heart arrhythmia (52/2820); heart attack / myocardial infarction (308/12078); heart failure / pulmonary odema (11/369); heart valve problem / heart murmur (65/3603); hypertrophic cardiomyopathy (HCM / HOCM) (<5/21); ischaemic stroke (<5/41); mitral regurgitation / incompetence (9/200); mitral valve disease (<5/73); peripheral vascular disease (20/936); pulmonary embolism +/- DVT (83/4292); stroke (182/7102); subarachnoid haemorrhage (16/471); SVT / supraventricular tachycardia (7/388); transient ischaemic attack (TIA) (47/2129); venous thromboembolic disease (<5/98); Wolff Parkinson White / WPW syndrome (<5/95) |
| Chronic respiratory disease | Asbestosis (6/202); asthma (1303/59527); bronchiectasis (31/1301); chronic obstructive airways disease / COPD (61/2032); emphysema (<5/217); emphysema / chronic bronchitis (174/7139); fibrosing alveolitis / unspecified alveolitis (<5/98); interstitial lung disease (<5/168); other respiratory problems (19/880); pulmonary fibrosis (<5/144) |
| Chronic kidney disease | Kidney nephropathy (<5/103); other renal / kidney problem (51/2246); polycystic kidney (5/371); renal / kidney failure (<5/268); renal failure not requiring dialysis (13/429); renal failure requiring dialysis (<5/273) |
| Diabetes | Diabetes (580/22873); diabetic eye disease (51/1366); diabetic nephropathy (<5/32); diabetic neuropathy / ulcers (<5/161); type 1 diabetes (10/526); type 2 diabetes (106/4429) |
| Hypertension | Essential hypertension (83/3606); hypertension (2664/135926) |
| Chronic liver disease | Alcoholic liver disease / alcoholic cirrhosis (<5/80); hepatitis B (<5/159); hepatitis C (6/153); liver failure / cirrhosis (7/374); non-infective hepatitis (7/539); primary biliary cirrhosis (<5/107) |
| Neurological disease | Cerebral palsy (<5/160); chronic / degenerative neurological problem (<5/178); dementia / Alzheimers / cognitive impairment (<5/152); epilepsy (88/4147); motor neurone disease (<5/65); multiple sclerosis (32/1831); myasthenia gravis (5/146); other demyelinating disease (not multiple sclerosis) (<5/80); other neurological problem (43/2043); paraplegia (<5/59); Parkinsons disease (31/948); polio / poliomyelitis (5/438); spinal cord disorder (9/505) |

^Self-reported diseases reported as they appear in UK Biobank.

Note: for each self-reported illness term, the affected number of patients diagnosed with COVID-19 and affected number of participants in UK Biobank are reported in brackets.

COPD, chronic obstructive pulmonary disease; IgA, immunoglobulin A; SLE, systemic lupus erythematosus; SVT, supraventricular tachycardia; WPW, Wolff Parkinson White.

Numbers are suppressed where there are <5 cases.

## Polygenic Risk Score Construction and Optimisation

### UK Biobank genotyping and quality control

Genotyping and imputation were each performed centrally by UK Biobank, as previously described.[1] Briefly, participants were genotyped using one of two arrays, the Affymetrix UK BiLEVE Axiom or Affymetrix UK Biobank Axiom array. Imputation of ~90 million genetic variants was then conducted using combined reference panels from the Haplotype Reference Consortium,[2] 1000 Genomes and UK10K projects.[3]

Samples were excluded from these analyses if they had high heterozygosity (PC-adjusted heterozygosity rates above the mean of 0.19), high missingness rates (> 5%), or if their self-reported sex did not match their genetic sex. Additionally, from each pair of closely related individuals (2^nd^ degree relatives or closer), the sample with the greater genotype missingness rate was excluded from further analyses.

Additional post-imputation quality control (QC) was then performed, including the removal of genetic variants with a minor allele frequency (MAF) < 0.001 and those with an imputation quality score below 0.8. A total of 10,152,250 genetic markers remained following QC.

Using a similar method to that utilized by UK Biobank,[1] following principal components analysis (PCA) of the linkage disequilibrium (LD)-thinned genotype data, ethnic outliers (non-Europeans) in PCs 1 and 2 were identified using the “aberrant” routine in R,[4] using a lambda parameter of 100. Those participants who both fell within the European PC cluster and self-reported as “White” in field 21000 (baseline data) were retained in the White European subgroup. This increased the sample size compared to UK Biobank’s “White British” definition, which excludes samples reported as “Irish” or “any other white background”. All samples, regardless of self-reported or genetically inferred ethnicity, were retained for transethnic analyses.

### Polygenic Risk Score Construction

Polygenic risk scores (PRS) for COVID-19 were constructed using summary statistics from a meta-GWAS conducted by the COVID19-hg consortium, a collaborative group formed to investigate host genetic contribution to COVID-19 susceptibility.[5]

Although our study outcomes of interest relate to COVID-19 severity, the COVID19-hg GWAS conducted several GWAS of different COVID-19 traits. The largest of these GWAS was the “COVID-19 vs. population" susceptibility European GWAS (32,494 cases; 1,316,207 controls, with the UK Biobank data removed), and transethnic GWAS (42,557 cases; 1,424,707 controls, with the UK Biobank data removed); the “hospitalized vs. non-hospitalized COVID-19" severity GWAS was substantially smaller in both the European cohort (8,316 hospitalized; 1,549,095 non-hospitalized, with the UK Biobank data removed) and the transethnic cohort (11,829 cases; 1,725,210 controls, with the UK Biobank data removed). A strong correlation has previously been observed between the results of GWAS of COVID-19 susceptibility and severity phenotypes[5], suggesting that it may be appropriate to use one phenotype as a proxy for the other in genetic analyses. There was a lack of power in the COVID19-hg[5] severity cohort to demonstrate the strong genetic correlation between the COVID-19 severity and COVID-19 susceptibility cohorts in Linkage Disequilibrium Score Regression (LDSC).[6, 7] However, a strong correlation was found between the European “hospitalized COVID-19 vs. population" and “COVID-19 vs. population" cohorts (*genetic correlation*=0.93, *SE*=0.14, *P*=1.04 x 10^-10^). We therefore opted to use the “COVID-19 vs. Population" susceptibility GWAS to maximize sample sizes and thus accuracy of effect estimates in preference to the smaller severity GWAS.

### Polygenic Risk Scores using European Effect Sizes

Three PRS were constructed using estimated effect sizes and *P*-values from the European susceptibility cohort (COVID19-hg 2021, excluding UK Biobank samples to ensure independence) in PRSice v2.3.3.[8] These PRS were then regressed on COVID-19 UK Biobank data with the first 10 genetic PCs included as covariates.

The first PRS, constructed using European effect sizes and *P*-values from the COVID19-hg susceptibility cohort (henceforth known as ‘PRS_e1_’) was generated using the clumping & thresholding method employed by PRSice. SNPs were thinned in 250kb blocks, based on an LD *r*^2^ threshold of 0.1, and prioritisation of SNPs at loci with the smallest GWAS *P*-values. All SNPs with *P*-values below a specified threshold (*P*^T^) in the phenotype summary statistics were used to form the PRS. COVID-19 susceptibility was then regressed on the PRS, using the UK Biobank European COVID-19 susceptibility dataset (cases *N=*7,274; controls *N=*36,370). This process was repeated for risk scores at many *P*^T^ (minimum *P*^T^=5 x 10^-8^; step size = 5 x 10^-5^; maximum *P*^T^=1). The best-fit risk score (PRS_e1_) was defined as the PRS with the strongest association with COVID-19 susceptibility in logistic regression. A permutation test was used to account for the testing of multiple risk scores, producing an empirical *P*-value for this association*.* The resulting optimised PRS_e1_ was then tested for association in the European UK Biobank COVID-19 severity cohort (hospitalized cases *N=*1,908; non-hospitalized controls *N*=5,366) using logistic regression. PRS_e1_ was also tested for association with COVID-19 severity in the transethnic UK Biobank samples (cases *N=*2,224; controls *N=*6,229) using logistic regression.

The second PRS was constructed using existing COVID-19 GWAS associations (**Supplementary Table S4**),[5, 9-11] defined as those genetic markers with a COVID-19 susceptibility or severity association in publicly available studies from all ethnicities(*P*<1 x 10^-5^, the “putative association” threshold as defined by the National Human Genome Research Institute).[12] Published COVID-19 GWAS associations were found through: (1) a systematic literature search using (1) Web of Science; (2) the ebi GWAS Catalog;[12] and (3) the ieu open GWAS project.[13] These SNPs were identified in the European COVID19-hg susceptibility summary statistics, and effect sizes from this GWAS were again used to construct a PRS (henceforth referred to as ‘PRS_e2_’). SNPs from PRS_e2_ were thinned (as above) to ensure that no markers in high LD were present in the score. PRS_e2_ was tested for association with COVID-19 severity in the European UK Biobank COVID-19 severity cohort using logistic regression, and PRS_e2_ was also tested for association in the transethnic UK Biobank COVID-19 severity cohort in a logistic regression.

Supplementary Table S4. Summary of studies providing genetic associations with COVID-19 phenotypes

| Study | Phenotype | | | Ethnicity | Cases (*N*)* | Controls (*N*)* |
| --- | --- | --- | --- | --- | --- | --- |
| 32558485[9] | Severe COVID-19 (hospitalization, respiratory failure & positive test) vs. population | | | European | 1980 | 2381 |
| 33307546[10] | Critically ill COVID-19 vs. population | | | European | 1069 | 1075 |
| 33536081[11]^ | COVID-19 mortality vs. Positive COVID-19 result | | | European | 445 | 1333 |
| 34237774[5] | COVID-19 vs. population (release 5; all versions) | | | European | 38,984 | 1,644,784 |
|  |  | | |  | (32,494) | (1,316,207) |
| 34237774[5] | COVID-19 vs. population (release 5; all versions) | | | Transethnic | 36,590 | 1,668,938 |
|  |  | | |  | (42,557) | (1,424,707) |
| 34237774[5] | Hospitalized COVID-19 vs. non- hospitalized COVID-19 (release 5; all versions) | | | European | 4,829 | 11,816 |
|  |  | | |  | (3,159) | (7,206) |
| 34237774[5] | Hospitalized COVID-19 vs. non- hospitalized COVID-19 (release 5; all versions) | | | Transethnic | 5,773 | 15,497 |
|  |  | | |  | (3,961) | (10,538) |
| 34237774[5] | Hospitalized COVID-19 vs. population (release 5; all versions) | | | European | 9,986 | 1,877,672 |
|  |  | | |  | (8,316) | (1,549,095) |
| 34237774[5] | Hospitalized COVID-19 vs. population (release 5; all versions) | | | Transethnic | 12,888 | 1,295,966 |
|  |  | | |  | (11,829) | (1,725,210) |
| 34237774[5] | Hospitalized COVID-19 vs. population (release 4; all versions) | | | Transethnic | 2,430 | 8,478 |
| 34237774[5] | Very severe respiratory confirmed COVID-19 vs. population (release 5; all versions) | | | European | 5,101 | 1,383,241 |
|  |  | | |  | (4,792) | (1,054,664) |
| 34237774[5] | Very severe respiratory confirmed COVID-19 vs. population (release 5; all versions) | | | Transethnic | 5,582 | 709,010 |
|  |  | | |  | (5,870) | (1,155,203) |
| 34237774[5] | Very severe respiratory confirmed COVID-19 vs. non-hospitalized COVID-19 (release 4) | | | Transethnic | 269 | 688 |
| Study | | Phenotype | Ethnicity | | Cases (*N*)* | Controls (*N*)* |
| 32558485[9] | | Severe COVID-19 (hospitalization, respiratory failure & positive test) vs. population | European | | 1980 | 2381 |
| 33307546[10] | | Critically ill COVID-19 vs. population | European | | 1069 | 1075 |
| 33536081[11]^ | | COVID-19 mortality vs. Positive COVID-19 result | European | | 445 | 1333 |
| 34237774[5] | | COVID-19 vs. population (release 5; all versions) | European | | 38,984 | 1,644,784 |
|  | |  |  | | (32,494) | (1,316,207) |
| 34237774[5] | | COVID-19 vs. population (release 5; all versions) | Transethnic | | 36,590 | 1,668,938 |
|  | |  |  | | (42,557) | (1,424,707) |
| 34237774[5] | | Hospitalized COVID-19 vs. non- hospitalized COVID-19 (release 5; all versions) | European | | 4,829 | 11,816 |
|  | |  |  | | (3,159) | (7,206) |
| 34237774[5] | | Hospitalized COVID-19 vs. non- hospitalized COVID-19 (release 5; all versions) | Transethnic | | 5,773 | 15,497 |
|  | |  |  | | (3,961) | (10,538) |
| 34237774[5] | | Hospitalized COVID-19 vs. population (release 5; all versions) | European | | 9,986 | 1,877,672 |
|  | |  |  | | (8,316) | (1,549,095) |
| 34237774[5] | | Hospitalized COVID-19 vs. population (release 5; all versions) | Transethnic | | 12,888 | 1,295,966 |
|  | |  |  | | (11,829) | (1,725,210) |
| 34237774[5] | | Hospitalized COVID-19 vs. population (release 4; all versions) | Transethnic | | 2,430 | 8,478 |
| 34237774[5] | | Very severe respiratory confirmed COVID-19 vs. population (release 5; all versions) | European | | 5,101 | 1,383,241 |
|  | |  |  | | (4,792) | (1,054,664) |
| 34237774[5] | | Very severe respiratory confirmed COVID-19 vs. population (release 5; all versions) | Transethnic | | 5,582 | 709,010 |
|  | |  |  | | (5,870) | (1,155,203) |
| 34237774[5] | | Very severe respiratory confirmed COVID-19 vs. non-hospitalized COVID-19 (release 4) | Transethnic | | 269 | 688 |

^described in publication as “super variants”.

*the number in brackets refers to the case/control numbers following removal of UK Biobank data.

The third PRS, again constructed using European effect sizes from the COVID19-hg susceptibility cohort, is referred to as PRS_e3_. This PRS was created by combining SNPs from PRS_e1_ with SNPs from PRS_e2_, and thinning SNPs to drop those in high LD between the two risk scores (as above). PRS_e3_ was tested for association with COVID-19 severity in the European UK Biobank cohort using logistic regression; PRS_e3_ was also tested association with the transethnic UK Biobank COVID-19 severity cohort in a logistic regression.

### Polygenic Risk Scores using Transethnic Effect Sizes

Three PRS were also constructed using effect sizes from the transethnic susceptibility cohort[5] in PRSice v2.3.3. The PRS were regressed on COVID-19 UK Biobank data with the first 10 genetic PCs included as covariates.

The first PRS (henceforth known as “PRS_t1_”) was constructed using the transethnic effect sizes and *P*-values from the COVID19-hg susceptibility cohort. Using the same methods as described for the construction of PRS_e1_, PRS_t1_ was generated with the clumping and thresholding technique implemented in PRSice v2.3.3.[8] SNPs were thinned using the parameters described above. All SNPs with *P*-values below a specified threshold (*P*^T^) in the phenotype summary statistics were used to form a PRS, which was then regressed on COVID-19 susceptibility in the UK Biobank European dataset. This process was repeated for risk scores at many *P*^T^ (as described above). The PRS with the strongest association with COVID-19 susceptibility in a logistic regression was selected as the best-fit risk score (PRS_t1_), and a permutation test was used to account for the testing of multiple risk scores. The best-fit PRS_t1_ was tested for association COVID-19 severity in the European UK Biobank COVID-19 severity cohort using logistic regression. A logistic regression was also performed to test for association between PRS_t1_ and COVID-19 severity in the transethnic UK Biobank COVID-19 severity cohort.

The second PRS (subsequently referred to as “PRS_t2_”) was created using transethnic effect sizes from the COVID19-hg susceptibility cohort. This PRS was constructed using the same SNPs as used in the production of PRS_e2_ , those previously associated with COVID-19 susceptibility or severity in public GWAS (**Supplementary Table S4**).[5, 9-11] To ensure that no markers in high LD were present in the score, SNPs from PRS_t2_ were thinned, and logistic regression was used to test for the association between PRS_t2_ and COVID-19 severity in the European UK Biobank cohort. PRS_t2_ was then also tested for association with COVID-19 severity in the transethnic UK Biobank cohort in a logistic regression.

The final PRS constructed using transethnic effect sizes from the COVID19-hg susceptibility cohort (subsequently referred to as “PRS_t3_”) was the combination of SNPs from PRS_t1_ and PRS_t2_. These SNPs were thinned using the parameters described above, and PRS_t3_ was tested for association with COVID-19 severity in the European UK Biobank cohort using logistic regression. Logistic regression was also used to test for association between PRS_t3_ and COVID-19 severity in the transethnic UK Biobank cohort.

### Comparison to a Published Polygenic Risk Score

A COVID-19 severity PRS was published whilst this manuscript was in preparation[14] and was constructed and tested in our UK Biobank cohort. This 64 SNP published PRS was originally constructed using a single inclusion threshold of *P*<0.0001, with effect sizes from a transethnic cohort consisting of 716 cases and 616 controls (COVID19-hg release 2), and using a hospitalized vs. non-hospitalized phenotype.[5] The PRS was originally tested for association in a UK Biobank COVID-19 severity cohort (hospitalized cases *N*=1,018; outpatient controls *N*=564) using logistic regression. This PRS (henceforth known as “PRS_d_”) was reconstructed in both of our COVID-19 severity cohorts to draw comparisons between this publicly-available PRS and our own. As with our own PRS, PRS_d_ was tested for association with COVID-19 severity in the European UK Biobank cohort using logistic regression, and again in the transethnic UK Biobank COVID-19 severity cohort. The strength of association was compared to ours using *P*-values and Nagelkerke's pseudo-*R^2^*.

### Ethnicity Meta-Analysis

To investigate the optimised PRS (PRS_e2_) association in the UK Biobank transethnic cohort in more detail, a meta-analysis was performed, testing the association of PRS_e2_ with severe COVID-19 in the individual ethnicities that comprised the full UK Biobank transethnic cohort, and assessing their contribution to the final result. PRS_e2_ was tested for association with COVID-19 severity using logistic regression in the following UK Biobank populations with a total sample *N*>50: (1) all ethnic groups combined (severe cases *N*=2,110, non-severe controls *N*=5,931); (2) “Mixed race” (severe cases *N*=14, non-severe controls *N*=40); (3) “Black” (severe cases *N*=79, non-severe controls *N*=148); (4) “Other” (severe cases *N*=25, non-severe controls *N*=98); (5) “Asian” (severe cases *N*=84, non-severe controls *N*=279); (6) European (severe cases *N*=1,908; non-severe controls *N*=5,366). PRS_e2_ was tested for association with severe COVID-19 in individual ethnic groups, and regression coefficient betas (log odds) for the ethnic groups were combined in an inverse-variance weighted fixed-effects meta-analysis to assess the overall efficacy of the score in the combined populations (both with and without the white subpopulation).

### Pathway Analyses

Following construction of PRS_e2_, annotation of SNPs in the PRS was performed by Ensembl Variant Effect Predictor (VEP),[15] and pathway analysis was performed using the Functional Mapping and Annotation of Genome-Wide Association Studies (FUMA) software v1.3.6a.[16] FUMA combines multiple *in silico* tools to provide biological interpretations of genetic markers. Amongst others, these tools include: ANNOVAR, which summarises information about the functional roles of each marker;[17] MAGMA, which performs gene-analysis using multiple regression, and identifies genes enriched with SNPs from PRS_e2_ (using a Bonferroni-adjusted significance level for the testing of 19,254 genes; 0.05/19,254 = 2.60 x 10^-6^);[18] and gene set enrichment analysis using multiple pathway databases (e.g. Reactome,[19] WikiPathways[20]). Regions analysed by FUMA were restricted to the genetic loci found in PRS_e2_, and the 1000 Genomes Phase 3 European panel[3] was used as a reference to perform LD thinning using the same parameters as PRSice (*r^2^*<0.1 in 250kb blocks).

## Supplementary Results

## Polygenic Risk Score Results

For each set of summary statistics from the COVID19-hg consortium (release 5: European and transethnic),[5] three PRS were constructed and tested for association with both the White European UK Biobank COVID-19 severity cohort and the transethnic UK Biobank COVID-19 severity cohort, using logistic regression (**Supplementary Table S5**).

Supplementary Table S5. Results of PRS tested in UK Biobank COVID-19 severity cohorts, using effect sizes from either European (PRSe1, PRSe2, PRSe3) or transethnic (PRSt1, PRSt2, PRSt3) COVID-19 susceptibility GWAS data from the COVID19-hg consortium.

| Effect Sizes Source | PRS |  |  | European UKB Severity Phenotype | | | Transethnic UKB Severity Phenotype | | |
| --- | --- | --- | --- | --- | --- | --- | --- | --- | --- |
|  |  | **Thres-hold** | ***N* SNPs** | **PRS *R*^2^** | **Coefficient [SE]** | ***P*** | **PRS *R*^2^** | **Coefficient [SE]** | ***P*** |
| European Effect Sizes | PRS_e1_ | 5 x 10^-8^ | 6 | 2.36 x 10^-4^ | 2.41 [2.22] | 0.28 | 3.35 x 10^-4^ | 2.89 [2.07] | 0.16 |
|  | PRS_e2_ | ·· | 133 | 2.35 x 10^-3^ | 82.03 [23.97] | 6.23 x 10^-4^ | 1.87 x 10^-3^ | 73.60 [22.33] | 9.81 x 10^-4^ |
|  | PRS_e3_ | ·· | 133 | 2.35 x 10^-3^ | 82.03 [23.97] | 6.23 x 10^-4^ | 1.87 x 10^-3^ | 73.60 [22.33] | 9.81 x 10^-4^ |
| Transethnic Effect Sizes | PRS_t1_ | 5 x 10^-8^ | 7 | 3.95 x 10^-4^ | 3.42 [2.43] | 0.16 | 5.68 x 10^-4^ | 4.13 [2.27] | 0.07 |
|  | PRS_t2_ | ·· | 129 | 1.37 x 10^-3^ | 65.34 [25] | 8.95 x 10^-3^ | 1.25 x 10^-3^ | 62.98 [23.4] | 7.11 x 10^-3^ |
|  | PRS_t3_ | ·· | 129 | 1.37 x 10^-3^ | 65.34 [25] | 8.95 x 10^-3^ | 1.25 x 10^-3^ | 62.98 [23.4] | 7.11 x 10^-3^ |
| Dite (2021) PRS | PRS_d_ | ·· | 64 | 1.80 x 10^-3^ | 3.54 [1.18] | 2.79 x 10^-3^ | 1.13 x 10^-3^ | 2.85 [1.11] | 9.7 x 10^-3^ |

Note: GWAS, genome-wide association study; SE, standard error; UKB, UK Biobank; PRS, polygenic risk score; R^2^, Nagelkerke’s R^2^; SNP, single nucleotide polymorphism

### Polygenic Risk Scores using European Effect Sizes

The construction of PRS_e1_ and logistic regression analysis of COVID-19 susceptibility in the UK Biobank cohort identified a 6 SNP risk score (*P^T^*=5 x 10^-8^) as having the best model fit (*R^2^*=1.88 x 10^-3^) for COVID-19 susceptibility. This PRS was then tested for association with the UK Biobank COVID-19 severity cohorts, along with PRS_e2_ and PRS_e3_. PRS_e1_ produced a weak association with COVID-19 severity in both the European (*P*=0.28) and transethnic (*P*=0.16) UK Biobank cohorts, whereas the 133 SNP PRS_e2_ demonstrated a strong association with COVID-19 severity in both UK Biobank cohorts (European *P*=6.23 x 10^-4^; transethnic *P*=9.81 x 10^-4^). Additionally, the estimated variance explained by the risk score was greater for PRS_e2_ than PRS_e1_ in both the European (PRS_e2_ *R*^2^=2.35 x 10^-3^, compared to PRS_e1_ *R*^2^=2.36 x 10^-4^) and transethnic UK Biobank COVID-19 severity cohorts (PRS_e2_ *R*^2^=1.87 x 10^-3^ compared to PRS_e1_ *R*^2^=3.35 x 10^-4^). PRS_e2_ had a positive coefficient in both UK Biobank cohorts (European *coefficient* [*SE*]=82.03 [23.97]; transethnic *coefficient* [*SE*]=73.60 [22.33]), suggesting that an increase in PRS_e2_ resulted in an increased risk of severe COVID-19 (**Supplementary Table S5**). The addition of PRS_e1_ to PRS_e2_ did not contribute any SNPs of predictive value to PRS_e2_, as the SNPs in PRS_e1_ were already present in PRS_e2_ from other studies. This meant that PRS_e2_ and PRS_e3_ were identical and that, of the three PRS tested using European effect sizes from COVID19-hg, PRS_e2_ was the optimal genetic predictor of severe COVID-19 in the European UK Biobank datasets.

### Polygenic Risk Scores using Transethnic Effect Sizes

As in the construction of PRS_e1_, severe COVID-19 was regressed on multiple PRS in the UK Biobank COVID-19 susceptibility cohort to determine PRS_t1_. This identified a PRS with 7 SNPs (*P*^T^=5 x 10^-8^) which had the best model fit (*R*^2^=1.43 x 10^-3^) for COVID-19 susceptibility and subsequently became PRS_t1_. PRS_t1_ was tested for association with the European and transethnic UK Biobank COVID-19 severity cohorts, along with PRS_t2_ and PRS_t3_. There was a weak association between PRS_t1_ and COVID-19 severity in both UK Biobank severity cohorts (European *P*=0.16; transethnic *P*=0.07), whilst a stronger association was found between PRS_t2_ and both the European (*P*=8.95 x 10^-3^) and transethnic (*P*=7.11 x 10^-3^) COVID-19 severity cohorts. The estimated variance explained by PRS_t2_ was also greater than PRS_t1_ in both the European (PRS_t2_ *R*^2^=1.37 x 10^-3^ compared to PRS_t1_ *R*^2^=3.95 x 10^-4^) and transethnic UK Biobank COVID-19 severity cohorts (PRS_t2_ *R*^2^=1.25 x 10^-3^, compared to PRS_t1_ *R*^2^=5.68 x 10^-4^). Furthermore, PRS_t2_ consisted of 129 SNPs, and had a positive coefficient in both UK Biobank cohorts (European *coefficient* [*SE*]=65.34 [*25*]; transethnic *coefficient* [*SE*]=62.98 [23.40]), indicating that an increase in PRS_t2_ was associated with an increased risk of severe COVID-19 (**Supplementary Table S5**). All SNPs from PRS_t1_ were already present in PRS_t2_, meaning that the addition of PRS_t1_ to PRS_t2_ did not contribute additional predictive value to PRS_t2_. Therefore, of the three PRS tested using transethnic effect sizes from COVID19-hg, PRS_t2_ was the best genetic predictor of severe COVID-19 in the UK Biobank datasets.

### Best-Fit Polygenic Risk Score

Of the two PRS (PRS_e2_ and PRS_t2_) that demonstrated the strongest association with both the European and transethnic UK Biobank COVID-19 severity cohorts, PRS_e2_ demonstrated the strongest association in both the European (PRS_e2_ *P*=6.23 x 10^-4^, compared to PRS_t2_ *P*=8.95 x 10^-3^) and the transethnic UK Biobank cohorts (PRS_e2_ *P*=9.81 x 10^-4^, compared to PRS_t2_ *P*=7.11 x 10^-3^). PRS_e2_ was also estimated to explain a greater level of variance in COVID-19 severity in both the European (PRS_e2_ *R*^2^=2.35 x 10^-3^, compared to PRS_t2_ *R*^2^=1.37 x 10^-3^) and transethnic UK Biobank cohorts (PRS_e2_ *R*^2^=1.87 x 10^-3^, compared to PRS_t2_ *R*^2^=1.25 x 10^-3^). PRS_e2_ was therefore selected as the best model for estimating genetic risk of COVID-19 severity, details of which may be found in **Supplementary Table S5**.

### Dite et al. (2021) Polygenic Risk Score

COVID-19 severity was regressed on the Dite et al.[14] risk score (PRS_d_) in the European and transethnic UK Biobank COVID-19 severity cohorts, and the strength of association, as well as the variance explained by PRS_d_ were compared with our own (PRS_e2_). The variance explained by PRS_e2_ was greater in the European UK Biobank cohort than that explained by PRS_d_ (*R*^2^=2.35 x 10^-3^, and 1.80 x 10^-3^ respectively; **Supplementary Table S5**), and the association between PRS_e2_ and severe COVID-19 was stronger than that of PRS_d_ (*P*=6.23 x 10^-4^ and *P*=2.79 x 10^-3^, respectively). The performance of PRS_d_ was also poor in the transethnic COVID-19 severity cohort when compared to PRS_e2_ (*R^2^*=1.13 x 10^-3^ and 1.87 x 10^-3^, respectively), and the strength of association between PRS_d_ and the transethnic cohort was again weaker than that of PRS_e2_ (*P*=9.70 x 10^-3^ and *P*=9.81 x 10^-4^ respectively).

### Ethnicity meta-analysis

A meta-analysis examining the association between PRS_e2_ and individual ethnic groups from the transethnic UK Biobank COVID-19 severity cohort was conducted, in order to assess the individual contribution of each ethnic group to the overall result. The coefficient for the fixed effects model (with Whites) was 0.08 (*SE*=0.03), *P*=2.49 x 10^-3^, and the I^2^ value was 0% suggesting little evidence of heterogeneity in the effect size between ethnicities (**Supplementary Figure S1; Supplementary Table S6**). This further suggests that there is a statistically significant association between PRS_e2_ and severe COVID-19 across populations. It is important to note that, although the betas of the White and Asian cohorts were in the positive direction, as expected (*betas* [*SE*]=0.09 [0.03] and 0.16 [0.13], respectively) and the betas of the “Other”, “Mixed” and Black cohorts were negative (“Other” *beta* [*SE*]=-0.31 [0.26]; “Mixed” *beta* [*SE*]=-0.27 [0.41]; Black *beta* [*SE*]=–0.08 [0.16]), however, the confidence intervals for these estimated effects were wide, most likely due to low sample size.


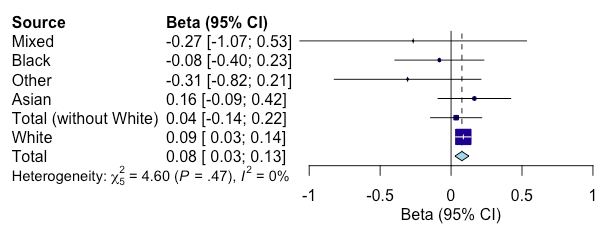


### Supplementary Figure S1. Forest plot summarizing results of the inverse-variance weighted, fixed effects meta-analyses of PRS_e2_, performed across multiple ethnic populations, including heterogeneity *X*^2^ and *I*^2^ statistics.

Supplementary Table S6. Results of the meta-analysis examining the association between PRS_e2_ and the transethnic UK Biobank severe COVID-19 case-control cohort.

| Ethnicity | Beta | Standard Error | *P*-value | Case (*N*) | Control (*N*) |
| --- | --- | --- | --- | --- | --- |
| Mixed | -0.27 | 0.41 | 0.51 | 14 | 40 |
| Black | -0.08 | 0.16 | 0.60 | 79 | 148 |
| Other | -0.31 | 0.26 | 0.25 | 25 | 98 |
| Asian | 0.16 | 0.13 | 0.21 | 84 | 279 |
| Combined (without Whites) | 0.04 | 0.09 | 0.97 | 202 | 565 |
| White | 0.09 | 0.03 | 1.60 x 10^-3^ | 1,908 | 5,366 |
| Combined (with Whites) | 0.08 | 0.03 | 2.49 x 10^-3^ | 2,110 | 5,931 |

Note: PRS_e2_, White European polygenic risk score 2.

## Descriptive results

There were 2362 (24.7%) patients who were hospitalised (26 in the 1-3 days prior, 1,648 on, and 688 within 28 days following their COVID-19 diagnosis date). Of those patients with an overnight stay (*n*=2137, 90.5%), 2089 were discharged during the study period and the median duration of hospitalisation was 8 days (IQR=4-17). There were 317 (3.3%) patients admitted into critical care within 1-3 days prior or 28 days following their COVID-19 diagnosis date. Of these, 144 received advanced respiratory support and 52 advanced cardiovascular support. For those who had an overnight critical care stay and were discharged from hospital during the study (*n*=166), the median duration of hospitalisation was 12 days (IQR=8-22). There were 750 (7.8%) patients who died within 100 days of their COVID-19 diagnosis date (median time to death: 11 days, IQR=4-22), of whom 577 (76.9%) were hospitalised prior to, or at the time of, death.

## Survival analysis results

Supplementary Table S7. Kaplan Meier probability estimates of severe COVID-19 and death in patients diagnosed with COVID-19, by sex

| **Outcome** | **Sex** | **Time (days)** | **All COVID-19 patients (*N=*9,560)** | | | | **White European subpopulation with COVID-19 (*N=*7,274)** | | |
| --- | --- | --- | --- | --- | --- | --- | --- | --- | --- |
|  |  |  | | ***N* risk** | **Cumulative event** | **Probability of event occurring within time window (95% CI)** | ***N* risk** | **Cumulative event** | **Probability of event occurring within time window (95% CI)** |
| **Severe COVID-19** | | | | | | | | | |
|  | Women | < 1 | | 4860 | 678 | 0.14 (0.13-0.15) | 3620 | 476 | 0.13 (0.12-0.14) |
|  |  | 1 | | 4182 | 728 | 0.15 (0.14-0.16) | 3144 | 513 | 0.14 (0.13-0.15) |
|  |  | 7 | | 4010 | 866 | 0.18 (0.17-0.19) | 3010 | 620 | 0.17 (0.16-0.18) |
|  |  | 28 | | 3123 | 993 | 0.21 (0.20-0.22) | 2358 | 721 | 0.20 (0.19-0.22) |
|  |  | 60 | | 1955 | 996 | 0.21 (0.20-0.22) | 1468 | 723 | 0.20 (0.19-0.22) |
|  |  | 100 | | 624 | 998 | 0.21 (0.20-0.22) | 457 | 724 | 0.20 (0.19-0.22) |
|  | Men | < 1 | | 4700 | 2071 | 0.23 (0.22-0.24) | 3654 | 1564 | 0.23 (0.22-0.24) |
|  |  | 1 | | 3627 | 2163 | 0.25 (0.24-0.26) | 2814 | 1635 | 0.25 (0.24-0.26) |
|  |  | 7 | | 3368 | 2353 | 0.29 (0.28-0.30) | 2628 | 1767 | 0.29 (0.27-0.30) |
|  |  | 28 | | 2585 | 2528 | 0.33 (0.32-0.34) | 2056 | 1902 | 0.33 (0.31-0.34) |
|  |  | 60 | | 1652 | 2530 | 0.33 (0.32-0.34) | 1315 | 1904 | 0.33 (0.31-0.34) |
|  |  | 100 | | 462 | 2535 | 0.33 (0.32-0.35) | 350 | 1908 | 0.33 (0.31-0.35) |
| **Death** | | | | | | | | | |
|  | Women | < 1 | | 4860 | 29 | 0.01 (0.004-0.01) | 3620 | 23 | 0.01 (0.00-0.01) |
|  |  | 1 | | 4831 | 35 | 0.01 (0.01-0.01) | 3597 | 29 | 0.01 (0.01-0.01) |
|  |  | 7 | | 4773 | 93 | 0.02 (0.02-0.02) | 3556 | 69 | 0.02 (0.02-0.02) |
|  |  | 28 | | 3845 | 202 | 0.04 (0.04-0.05) | 2886 | 147 | 0.04 (0.04-0.05) |
|  |  | 60 | | 2513 | 234 | 0.05 (0.05-0.06) | 1868 | 169 | 0.05 (0.04-0.06) |
|  |  | 100 | | 991 | 251 | 0.06 (0.06-0.07) | 712 | 182 | 0.06 (0.05-0.07) |
|  | Men | < 1 | | 4700 | 307 | 0.01 (0.01-0.02) | 3654 | 227 | 0.01 (0.01-0.02) |
|  |  | 1 | | 4644 | 317 | 0.01 (0.01-0.02) | 3609 | 236 | 0.02 (0.01-0.02) |
|  |  | 7 | | 4700 | 307 | 0.04 (0.03-0.05) | 3516 | 327 | 0.04 (0.03-0.05) |
|  |  | 28 | | 3600 | 670 | 0.09 (0.09-0.10) | 2833 | 520 | 0.10 (0.09-0.11) |
|  |  | 60 | | 2423 | 723 | 0.11 (0.10-0.12) | 1895 | 566 | 0.11 (0.10-0.12) |
|  |  | 100 | | 970 | 750 | 0.12 (0.11-0.13) | 728 | 590 | 0.13 (0.12-0.14) |

Note: severe COVID-19 is defined as the earliest of a recorded hospitalisation or critical care admission within 28 days of COVID-19 diagnosis, or death within 100 days of COVID-19 diagnosis.

Supplementary Table S8. Kaplan Meier probability estimates of severe COVID-19 and death in patients diagnosed with COVID-19, per ethnicity (*N=*9,507)

| **Group** | **Time (days)** | **Severe COVID-19** | | | **Death** | | |
| --- | --- | --- | --- | --- | --- | --- | --- |
|  |  | ***N* risk** | **Cumulative event** | **Probability of event occurring within time window (95% CI)** | ***N* risk** | **Cumulative event** | **Probability of event occurring within time window (95% CI)** |
| White | < 1 | 8645 | 1564 | 0.18 (0.17-0.19) | 8645 | 76 | 0.01 (0.01-0.01) |
|  | 1 | 7081 | 1685 | 0.20 (0.19-0.20) | 8569 | 92 | 0.01 (0.01-0.01) |
|  | 7 | 6706 | 1976 | 0.23 (0.22-0.24) | 8412 | 248 | 0.03 (0.03-0.03) |
|  | 28 | 5237 | 2256 | 0.26 (0.25-0.27) | 6795 | 561 | 0.07 (0.06-0.07) |
|  | 60 | 3306 | 2261 | 0.27 (0.26-0.27) | 4480 | 644 | 0.08 (0.08-0.09) |
|  | 100 | 946 | 2268 | 0.27 (0.26-0.28) | 1693 | 685 | 0.10 (0.09-0.10) |
| Black | < 1 | 253 | 2336 | 0.27 (0.21-0.32) | 253 | 687 | 0.01 (0.00-0.02) |
|  | 1 | 185 | 2345 | 0.30 (0.25-0.36) | 251 | 687 | 0.01 (0.00-0.02) |
|  | 7 | 169 | 2353 | 0.34 (0.28-0.39) | 241 | 700 | 0.06 (0.03-0.09) |
|  | 28 | 111 | 2359 | 0.36 (0.30-0.42) | 163 | 711 | 0.11 (0.07-0.15) |
|  | 60 | 72 | 2359 | 0.36 (0.30-0.42) | 120 | 712 | 0.11 (0.07-0.15) |
|  | 100 | 43 | 2359 | 0.36 (0.30-0.42) | 85 | 713 | 0.12 (0.08-0.17) |
| Other | < 1 | 609 | 2458 | 0.16 (0.13-0.19) | 609 | 718 | 0.01 (0.00-0.02) |
|  | 1 | 510 | 2466 | 0.18 (0.15-0.21) | 604 | 718 | 0.01 (0.00-0.02) |
|  | 7 | 476 | 2493 | 0.22 (0.19-0.25) | 598 | 724 | 0.02 (0.01-0.03) |
|  | 28 | 340 | 2508 | 0.25 (0.21-0.28) | 450 | 739 | 0.05 (0.03-0.06) |
|  | 60 | 217 | 2508 | 0.25 (0.21-0.28) | 310 | 740 | 0.05 (0.03-0.07) |
|  | 100 | 94 | 2508 | 0.25 (0.21-0.28) | 171 | 742 | 0.05 (0.03-0.07) |

Note: severe COVID-19 is defined as the earliest of a recorded hospitalisation or critical care admission within 28 days of COVID-19 diagnosis, or death within 100 days of COVID-19 diagnosis.

Supplementary Table S9. Kaplan Meier probability estimates of severe COVID-19 and death in patients diagnosed with COVID-19, per smoking status

| **Outcome** | **Smoking Status** | **Time (days)** | **All COVID-19 patients (*N=*9,496)** | | | **White European subpopulation with COVID-19 (*N=*7,244)** | | | | |  |
| --- | --- | --- | --- | --- | --- | --- | --- | --- | --- | --- | --- |
|  |  |  | ***N* risk** | **Cumulative event** | **Probability of event occurring within time window (95% CI)** | ***N* risk** | | **Cumulative event** | **Probability of event occurring within time window (95% CI)** | | |
| **Severe COVID-19** | | | | | | | | | | |  |
|  | Never | < 1 | 4903 | 712 | 0.15 (0.14-0.16) | 3611 | 507 | | | 0.14 (0.13-0.15) |  |
|  |  | 1 | 4191 | 777 | 0.16 (0.15-0.17) | 3104 | 555 | | | 0.15 (0.14-0.17) |  |
|  |  | 7 | 4015 | 904 | 0.18 (0.17-0.20) | 2977 | 644 | | | 0.18 (0.17-0.19) |  |
|  |  | 28 | 3133 | 1040 | 0.22 (0.20-0.23) | 2355 | 745 | | | 0.21 (0.20-0.22) |  |
|  |  | 60 | 1982 | 1042 | 0.22 (0.20-0.23) | 1487 | 747 | | | 0.21 (0.20-0.22) |  |
|  |  | 100 | 604 | 1044 | 0.22 (0.21-0.23) | 424 | 748 | | | 0.21 (0.20-0.22) |  |
|  | Former | < 1 | 3506 | 1790 | 0.21 (0.20-0.23) | 2807 | 1340 | | | 0.21 (0.20-0.23) |  |
|  |  | 1 | 2760 | 1848 | 0.23 (0.22-0.24) | 2215 | 1387 | | | 0.23 (0.21-0.24) |  |
|  |  | 7 | 2554 | 2014 | 0.28 (0.26-0.29) | 2055 | 1513 | | | 0.27 (0.26-0.29) |  |
|  |  | 28 | 1964 | 2131 | 0.31 (0.30-0.33) | 1598 | 1608 | | | 0.31 (0.29-0.33) |  |
|  |  | 60 | 1253 | 2133 | 0.31 (0.30-0.33) | 1023 | 1610 | | | 0.31 (0.29-0.33) |  |
|  |  | 100 | 370 | 2138 | 0.32 (0.30-0.34) | 298 | 1614 | | | 0.32 (0.30-0.33) |  |
|  | Current | < 1 | 1087 | 2405 | 0.25 (0.22-0.27) | 826 | 1820 | | | 0.25 (0.22-0.28) |  |
|  |  | 1 | 820 | 2423 | 0.26 (0.24-0.29) | 620 | 1833 | | | 0.27 (0.23-0.30) |  |
|  |  | 7 | 777 | 2452 | 0.29 (0.26-0.32) | 590 | 1853 | | | 0.29 (0.26-0.32) |  |
|  |  | 28 | 586 | 2499 | 0.34 (0.31-0.36) | 448 | 1891 | | | 0.34 (0.31-0.37) |  |
|  |  | 60 | 353 | 2500 | 0.34 (0.31-0.37) | 261 | 1891 | | | 0.34 (0.31-0.37) |  |
|  |  | 100 | 107 | 2500 | 0.34 (0.31-0.37) | 81 | 1891 | | | 0.34 (0.31-0.37) |  |
| **Death** | | | | | | | | | |  |  |
|  | Never | < 1 | 4903 | 31 | 0.01 (0.00-0.01) | 3611 | 24 | | | 0.01 (0.00-0.01) |  |
|  |  | 1 | 4872 | 39 | 0.01 (0.01-0.01) | 3587 | 32 | | | 0.01 (0.01-0.01) |  |
|  |  | 7 | 4811 | 94 | 0.02 (0.02-0.02) | 3542 | 70 | | | 0.02 (0.02-0.02) |  |
|  |  | 28 | 3874 | 213 | 0.05 (0.04-0.05) | 2893 | 156 | | | 0.05 (0.04-0.05) |  |
|  |  | 60 | 2560 | 245 | 0.06 (0.05-0.06) | 1894 | 181 | | | 0.06 (0.05-0.06) |  |
|  |  | 100 | 1002 | 262 | 0.07 (0.06-0.07) | 702 | 194 | | | 0.07 (0.06-0.08) |  |
|  | Former | < 1 | 3506 | 299 | 0.01 (0.01-0.01) | 2807 | 227 | | | 0.01 (0.01-0.02) |  |
|  |  | 1 | 3469 | 306 | 0.01 (0.01-0.02) | 2774 | 233 | | | 0.01 (0.01-0.02) |  |
|  |  | 7 | 3376 | 406 | 0.04 (0.03-0.05) | 2701 | 310 | | | 0.04 (0.03-0.05) |  |
|  |  | 28 | 2683 | 559 | 0.09 (0.08-0.10) | 2164 | 436 | | | 0.09 (0.08-0.10) |  |
|  |  | 60 | 1799 | 596 | 0.10 (0.09-0.11) | 1449 | 467 | | | 0.10 (0.09-0.12) |  |
|  |  | 100 | 709 | 619 | 0.12 (0.11-0.13) | 561 | 487 | | | 0.12 (0.11-0.14) |  |
|  | Current | < 1 | 1087 | 634 | 0.01 (0.01-0.02) | 826 | 498 | | | 0.01 (0.01-0.02) |  |
|  |  | 1 | 1072 | 635 | 0.02 (0.01-0.02) | 815 | 499 | | | 0.02 (0.01-0.02) |  |
|  |  | 7 | 1053 | 655 | 0.03 (0.02-0.04) | 800 | 514 | | | 0.03 (0.02-0.05) |  |
|  |  | 28 | 839 | 719 | 0.10 (0.08-0.11) | 638 | 568 | | | 0.10 (0.08-0.12) |  |
|  |  | 60 | 538 | 735 | 0.12 (0.10-0.14) | 399 | 580 | | | 0.12 (0.10-0.15) |  |
|  |  | 100 | 231 | 738 | 0.13 (0.10-0.15) | 168 | 583 | | | 0.14 (0.11-0.16) |  |

Note: severe COVID-19 is defined as the earliest of a recorded hospitalisation or critical care admission within 28 days of COVID-19 diagnosis, or death within 100 days of COVID-19 diagnosis.

Supplementary Table S10. Kaplan Meier probability estimates of severe COVID-19 and death in patients diagnosed with COVID-19, per Townsend deprivation quintile

| **Outcome** | **Quintile** | **Time (days)** | **All COVID-19 patients (*N=*9,552)** | | | **White European subpopulation with COVID-19 (*N=*7,268)** | | |
| --- | --- | --- | --- | --- | --- | --- | --- | --- |
|  |  |  | ***N* risk** | **Cumulative event** | **Probability of event occurring within time window (95% CI)** | ***N* risk** | **Cumulative event** | **Probability of event occurring within time window (95% CI)** |
| **Severe COVID-19** | | | | | | | | |
|  | 1 (Least deprived) | < 1 | 1476 | 229 | 0.16 (0.14-0.17) | 1231 | 196 | 0.16 (0.14-0.18) |
|  |  | 1 | 1247 | 244 | 0.17 (0.15-0.18) | 1035 | 211 | 0.17 (0.15-0.19) |
|  |  | 7 | 1183 | 304 | 0.21 (0.19-0.23) | 982 | 257 | 0.21 (0.19-0.23) |
|  |  | 28 | 926 | 353 | 0.24 (0.22-0.26) | 778 | 297 | 0.24 (0.22-0.27) |
|  |  | 60 | 563 | 353 | 0.24 (0.22-0.26) | 463 | 297 | 0.24 (0.22-0.27) |
|  |  | 100 | 167 | 354 | 0.25 (0.22-0.27) | 138 | 298 | 0.25 (0.22-0.27) |
|  | 2 | < 1 | 1691 | 625 | 0.16 (0.14-0.18) | 1406 | 525 | 0.16 (0.14-0.18) |
|  |  | 1 | 1420 | 650 | 0.18 (0.16-0.19) | 1179 | 544 | 0.18 (0.16-0.20) |
|  |  | 7 | 1357 | 698 | 0.20 (0.18-0.22) | 1129 | 583 | 0.20 (0.18-0.22) |
|  |  | 28 | 1043 | 746 | 0.24 (0.21-0.26) | 871 | 623 | 0.23 (0.21-0.26) |
|  |  | 60 | 667 | 747 | 0.24 (0.22-0.26) | 555 | 624 | 0.24 (0.21-0.26) |
|  |  | 100 | 192 | 749 | 0.24 (0.22-0.26) | 161 | 625 | 0.24 (0.21-0.26) |
|  | 3 | < 1 | 1836 | 1049 | 0.16 (0.15-0.18) | 1459 | 864 | 0.16 (0.15-0.18) |
|  |  | 1 | 1536 | 1068 | 0.17 (0.16-0.19) | 1220 | 883 | 0.18 (0.16-0.20) |
|  |  | 7 | 1470 | 1121 | 0.20 (0.18-0.22) | 1166 | 920 | 0.20 (0.18-0.22) |
|  |  | 28 | 1174 | 1175 | 0.23 (0.21-0.25) | 939 | 959 | 0.23 (0.21-0.25) |
|  |  | 60 | 734 | 1175 | 0.23 (0.21-0.25) | 583 | 959 | 0.23 (0.21-0.25) |
|  |  | 100 | 209 | 1176 | 0.24 (0.22-0.26) | 159 | 960 | 0.23 (0.21-0.25) |
|  | 4 | < 1 | 1996 | 1544 | 0.18 (0.17-0.20) | 1497 | 1237 | 0.19 (0.17-0.20) |
|  |  | 1 | 1628 | 1573 | 0.20 (0.18-0.22) | 1220 | 1260 | 0.20 (0.18-0.22) |
|  |  | 7 | 1538 | 1636 | 0.23 (0.21-0.25) | 1152 | 1307 | 0.23 (0.21-0.25) |
|  |  | 28 | 1198 | 1696 | 0.26 (0.24-0.28) | 912 | 1358 | 0.27 (0.25-0.29) |
|  |  | 60 | 759 | 1697 | 0.26 (0.24-0.28) | 583 | 1359 | 0.27 (0.25-0.29) |
|  |  | 100 | 235 | 1698 | 0.27 (0.25-0.28) | 173 | 1360 | 0.27 (0.25-0.29) |
|  | 5 (Most deprived) | < 1 | 2553 | 2281 | 0.23 (0.21-0.24) | 1675 | 1737 | 0.23 (0.21-0.25) |
|  |  | 1 | 1970 | 2335 | 0.25 (0.23-0.27) | 1298 | 1769 | 0.24 (0.22-0.26) |
|  |  | 7 | 1822 | 2439 | 0.29 (0.27-0.31) | 1203 | 1839 | 0.29 (0.26-0.31) |
|  |  | 28 | 1362 | 2529 | 0.33 (0.31-0.35) | 910 | 1904 | 0.33 (0.31-0.35) |
|  |  | 60 | 883 | 2532 | 0.33 (0.31-0.35) | 598 | 1906 | 0.33 (0.31-0.35) |
|  |  | 100 | 283 | 2534 | 0.34 (0.32-0.35) | 176 | 1907 | 0.33 (0.31-0.36) |
| **Death** | | | | | | | | |
|  | 1 (Least deprived) | < 1 | 1476 | 16 | 0.01 (0.01-0.02) | 1231 | 13 | 0.01 (0.01-0.02) |
|  |  | 1 | 1460 | 21 | 0.01 (0.01-0.02) | 1218 | 18 | 0.02 (0.01-0.02) |
|  |  | 7 | 1431 | 46 | 0.03 (0.02-0.04) | 1191 | 41 | 0.03 (0.02-0.04) |
|  |  | 28 | 1165 | 89 | 0.06 (0.05-0.08) | 978 | 78 | 0.07 (0.05-0.08) |
|  |  | 60 | 745 | 100 | 0.07 (0.06-0.09) | 616 | 89 | 0.08 (0.06-0.09) |
|  |  | 100 | 276 | 110 | 0.09 (0.07-0.11) | 231 | 98 | 0.10 (0.08-0.12) |
|  | 2 | < 1 | 1691 | 122 | 0.01 (0.00-0.01) | 1406 | 108 | 0.01 (0.00-0.01) |
|  |  | 1 | 1679 | 124 | 0.01 (0.00-0.01) | 1396 | 110 | 0.01 (0.00-0.01) |
|  |  | 7 | 1654 | 151 | 0.02 (0.02-0.03) | 1375 | 131 | 0.02 (0.02-0.03) |
|  |  | 28 | 1312 | 205 | 0.06 (0.05-0.07) | 1096 | 176 | 0.06 (0.05-0.07) |
|  |  | 60 | 875 | 219 | 0.07 (0.06-0.08) | 727 | 187 | 0.07 (0.06-0.08) |
|  |  | 100 | 326 | 227 | 0.09 (0.07-0.1.) | 273 | 193 | 0.08 (0.06-0.10) |
|  | 3 | < 1 | 1836 | 245 | 0.01 (0.01-0.01) | 1459 | 209 | 0.01 (0.01-0.02) |
|  |  | 1 | 1818 | 247 | 0.01 (0.01-0.02) | 1443 | 211 | 0.01 (0.01-0.02) |
|  |  | 7 | 1790 | 275 | 0.03 (0.02-0.03) | 1422 | 231 | 0.03 (0.02-0.03) |
|  |  | 28 | 1474 | 336 | 0.06 (0.05-0.07) | 1171 | 280 | 0.06 (0.05-0.08) |
|  |  | 60 | 955 | 354 | 0.08 (0.06-0.09) | 750 | 293 | 0.07 (0.06-0.09) |
|  |  | 100 | 364 | 361 | 0.09 (0.07-0.1.) | 278 | 299 | 0.09 (0.07-0.10) |
|  | 4 | < 1 | 1996 | 377 | 0.01 (0.00-0.01) | 1497 | 312 | 0.01 (0.00-0.01) |
|  |  | 1 | 1980 | 379 | 0.01 (0.01-0.01) | 1484 | 314 | 0.01 (0.01-0.02) |
|  |  | 7 | 1945 | 415 | 0.03 (0.02-0.03) | 1458 | 340 | 0.03 (0.02-0.04) |
|  |  | 28 | 1568 | 483 | 0.06 (0.05-0.08) | 1195 | 397 | 0.07 (0.06-0.08) |
|  |  | 60 | 1035 | 501 | 0.08 (0.06-0.09) | 796 | 410 | 0.08 (0.07-0.09) |
|  |  | 100 | 423 | 508 | 0.09 (0.07-0.10) | 315 | 417 | 0.09 (0.08-0.11) |
|  | 5 (Most deprived) | < 1 | 2553 | 531 | 0.01 (0.01-0.01) | 1675 | 433 | 0.01 (0.01-0.01) |
|  |  | 1 | 2530 | 536 | 0.01 (0.01-0.02) | 1659 | 437 | 0.01 (0.01-0.02) |
|  |  | 7 | 2472 | 597 | 0.04 (0.03-0.04) | 1620 | 478 | 0.04 (0.03-0.05) |
|  |  | 28 | 1921 | 713 | 0.09 (0.07-0.10) | 1275 | 560 | 0.09 (0.08-0.10) |
|  |  | 60 | 1325 | 737 | 0.10 (0.09-0.11) | 873 | 580 | 0.11 (0.09-0.12) |
|  |  | 100 | 572 | 749 | 0.11 (0.10-0.12) | 343 | 589 | 0.12 (0.10-0.14) |

Note: severe COVID-19 is defined as the earliest of a recorded hospitalisation or critical care admission within 28 days of COVID-19 diagnosis, or death within 100 days of COVID-19 diagnosis.

Supplementary Table S11. Kaplan Meier probability estimates of severe COVID-19 and death in patients diagnosed with COVID-19, per BMI group

| **Outcome** | **BMI (kg/m^2^)** | **Time (days)** | **All COVID-19 patients (*N=*9,485)** | | | **White European subpopulation with COVID-19 (*N=*7,241)** | | |
| --- | --- | --- | --- | --- | --- | --- | --- | --- |
|  |  |  | ***N* risk** | **Cumulative event** | **Probability of event occurring within time window (95% CI)** | ***N* risk** | **Cumulative event** | **Probability of event occurring within time window (95% CI)** |
| **Severe COVID-19** | | | | | | | |  |
|  | <18.5 | < 1 | 33 | 10 | 0.30 (0.13-0.44) | 23 | 9 | 0.39 (0.16-0.56) |
|  |  | 1 | 23 | 10 | 0.30 (0.13-0.44) | 14 | 9 | 0.39 (0.16-0.56) |
|  |  | 7 | 22 | 11 | 0.33 (0.15-0.48) | 14 | 9 | 0.39 (0.16-0.56) |
|  |  | 28 | 15 | 12 | 0.36 (0.18-0.51) | 11 | 9 | 0.39 (0.16-0.56) |
|  |  | 60 | 6 | 12 | 0.36 (0.18-0.51) | <5 | 9 | 0.39 (0.16-0.56) |
|  |  | 100 | <5 | 12 | 0.36 (0.18-0.51) | <5 | 9 | 0.39 (0.16-0.56) |
|  | 18.5 to <25 | < 1 | 2472 | 338 | 0.13 (0.12-0.15) | 1878 | 265 | 0.14 (0.12-0.15) |
|  |  | 1 | 2146 | 362 | 0.14 (0.13-0.16) | 1622 | 283 | 0.15 (0.13-0.16) |
|  |  | 7 | 2074 | 419 | 0.17 (0.15-0.18) | 1573 | 319 | 0.17 (0.15-0.18) |
|  |  | 28 | 1600 | 467 | 0.19 (0.17-0.20) | 1229 | 356 | 0.19 (0.17-0.20) |
|  |  | 60 | 1005 | 469 | 0.19 (0.17-0.20) | 766 | 357 | 0.19 (0.17-0.21) |
|  |  | 100 | 304 | 471 | 0.19 (0.18-0.21) | 235 | 358 | 0.19 (0.17-0.21) |
|  | 25 to <30 | < 1 | 3988 | 1176 | 0.18 (0.17-0.19) | 3061 | 889 | 0.17 (0.16-0.19) |
|  |  | 1 | 3283 | 1226 | 0.19 (0.18-0.20) | 2530 | 931 | 0.19 (0.17-0.20) |
|  |  | 7 | 3126 | 1350 | 0.22 (0.21-0.23) | 2411 | 1019 | 0.22 (0.20-0.23) |
|  |  | 28 | 2450 | 1476 | 0.26 (0.24-0.27) | 1903 | 1119 | 0.25 (0.24-0.27) |
|  |  | 60 | 1584 | 1478 | 0.26 (0.24-0.27) | 1219 | 1121 | 0.25 (0.24-0.27) |
|  |  | 100 | 469 | 1480 | 0.26 (0.24-0.27) | 343 | 1122 | 0.25 (0.24-0.27) |
|  | 30 to <35 | < 1 | 2066 | 1903 | 0.21 (0.19-0.22) | 1598 | 1446 | 0.20 (0.18-0.22) |
|  |  | 1 | 1643 | 1943 | 0.22 (0.21-0.24) | 1274 | 1478 | 0.22 (0.20-0.24) |
|  |  | 7 | 1515 | 2040 | 0.27 (0.25-0.29) | 1174 | 1554 | 0.27 (0.25-0.29) |
|  |  | 28 | 1165 | 2120 | 0.31 (0.29-0.33) | 923 | 1619 | 0.31 (0.29-0.34) |
|  |  | 60 | 713 | 2120 | 0.31 (0.29-0.33) | 573 | 1619 | 0.31 (0.29-0.34) |
|  |  | 100 | 207 | 2121 | 0.32 (0.29-0.33) | 154 | 1620 | 0.32 (0.29-0.34) |
|  | 35 to <40 | < 1 | 655 | 2301 | 0.28 (0.24-0.31) | 477 | 1749 | 0.27 (0.23-0.31) |
|  |  | 1 | 475 | 2318 | 0.30 (0.27-0.34) | 348 | 1759 | 0.29 (0.25-0.33) |
|  |  | 7 | 426 | 2352 | 0.35 (0.32-0.39) | 311 | 1788 | 0.35 (0.31-0.39) |
|  |  | 28 | 321 | 2379 | 0.40 (0.36-0.43) | 238 | 1806 | 0.39 (0.35-0.44) |
|  |  | 60 | 197 | 2380 | 0.40 (0.36-0.44) | 143 | 1807 | 0.40 (0.35-0.44) |
|  |  | 100 | 65 | 2382 | 0.41 (0.37-0.45) | 44 | 1809 | 0.41 (0.36-0.45) |
|  | ≥40 | < 1 | 271 | 2463 | 0.30 (0.24-0.35) | 204 | 1865 | 0.28 (0.21-0.33) |
|  |  | 1 | 190 | 2470 | 0.33 (0.27-0.38) | 148 | 1869 | 0.29 (0.23-0.35) |
|  |  | 7 | 172 | 2482 | 0.37 (0.31-0.42) | 135 | 1879 | 0.34 (0.28-0.41) |
|  |  | 28 | 123 | 2498 | 0.44 (0.37-0.49) | 95 | 1893 | 0.42 (0.35-0.49) |
|  |  | 60 | 81 | 2498 | 0.44 (0.37-0.49) | 65 | 1893 | 0.42 (0.35-0.49) |
|  |  | 100 | 30 | 2498 | 0.44 (0.37-0.49) | 25 | 1893 | 0.42 (0.35-0.49) |
| **Death** | | | | | | | |  |
|  | <18.5 | < 1 | 33 | 21 | 0.01 (0.01-0.01) | 23 | <5 | ·· |
|  |  | 1 | 32 | 24 | 0.01 (0.01-0.01) | 23 | <5 | ·· |
|  |  | 7 | 31 | 48 | 0.02 (0.01-0.03) | 22 | <5 | ·· |
|  |  | 28 | 22 | 107 | 0.05 (0.04-0.05) | 17 | <5 | ·· |
|  |  | 60 | 11 | 127 | 0.06 (0.05-0.07) | 9 | <5 | ·· |
|  |  | 100 | 5 | 139 | 0.07 (0.06-0.09) | <5 | <5 | ·· |
|  | 18.5 to <25 | < 1 | 2472 | 140 | 0.03 (0.00-0.09) | 1878 | 22 | 0.01 (0.01-0.02) |
|  |  | 1 | 2451 | 140 | 0.03 (0.00-0.09) | 1859 | 25 | 0.01 (0.01-0.02) |
|  |  | 7 | 2425 | 141 | 0.06 (0.00-0.14) | 1838 | 43 | 0.02 (0.02-0.03) |
|  |  | 28 | 1920 | 144 | 0.16 (0.02-0.28) | 1466 | 94 | 0.05 (0.04-0.06) |
|  |  | 60 | 1252 | 144 | 0.16 (0.02-0.28) | 943 | 109 | 0.06 (0.05-0.07) |
|  |  | 100 | 468 | 144 | 0.16 (0.02-0.28) | 354 | 120 | 0.08 (0.07-0.10) |
|  | 25 to <30 | < 1 | 3988 | 178 | 0.01 (0.01-0.01) | 3061 | 148 | 0.01 (0.01-0.01) |
|  |  | 1 | 3954 | 184 | 0.01 (0.01-0.01) | 3033 | 154 | 0.01 (0.01-0.02) |
|  |  | 7 | 3892 | 248 | 0.03 (0.02-0.03) | 2986 | 201 | 0.03 (0.02-0.03) |
|  |  | 28 | 3146 | 391 | 0.07 (0.06-0.07) | 2429 | 319 | 0.07 (0.06-0.08) |
|  |  | 60 | 2133 | 418 | 0.07 (0.07-0.08) | 1629 | 339 | 0.08 (0.07-0.09) |
|  |  | 100 | 843 | 436 | 0.09 (0.08-0.10) | 617 | 352 | 0.09 (0.08-0.10) |
|  | 30 to <35 | < 1 | 2066 | 451 | 0.01 (0.00-0.01) | 1598 | 363 | 0.01 (0.00-0.01) |
|  |  | 1 | 2051 | 458 | 0.01 (0.01-0.02) | 1587 | 369 | 0.01 (0.01-0.02) |
|  |  | 7 | 1998 | 509 | 0.04 (0.03-0.04) | 1542 | 411 | 0.04 (0.03-0.05) |
|  |  | 28 | 1599 | 593 | 0.08 (0.07-0.09) | 1258 | 478 | 0.08 (0.07-0.10) |
|  |  | 60 | 1024 | 613 | 0.09 (0.08-0.11) | 807 | 497 | 0.10 (0.08-0.11) |
|  |  | 100 | 410 | 620 | 0.10 (0.09-0.12) | 302 | 503 | 0.11 (0.09-0.12) |
|  | 35 to <40 | < 1 | 655 | 628 | 0.01 (0.00-0.02) | 477 | 509 | 0.01 (0.00-0.02) |
|  |  | 1 | 647 | 628 | 0.01 (0.00-0.02) | 471 | 509 | 0.01 (0.00-0.02) |
|  |  | 7 | 629 | 648 | 0.04 (0.03-0.06) | 460 | 521 | 0.04 (0.02-0.06) |
|  |  | 28 | 498 | 684 | 0.10 (0.08-0.13) | 370 | 547 | 0.10 (0.07-0.12) |
|  |  | 60 | 338 | 695 | 0.12 (0.10-0.15) | 249 | 556 | 0.12 (0.09-0.15) |
|  |  | 100 | 154 | 700 | 0.14 (0.11-0.17) | 106 | 561 | 0.15 (0.11-0.19) |
|  | ≥40 | < 1 | 271 | 704 | 0.02 (0.00-0.03) | 204 | 564 | 0.02 (0.00-0.03) |
|  |  | 1 | 267 | 704 | 0.02 (0.00-0.03) | 201 | 564 | 0.02 (0.00-0.03) |
|  |  | 7 | 259 | 714 | 0.05 (0.03-0.08) | 196 | 571 | 0.05 (0.02-0.08) |
|  |  | 28 | 202 | 728 | 0.11 (0.07-0.15) | 154 | 578 | 0.09 (0.05-0.13) |
|  |  | 60 | 141 | 733 | 0.13 (0.09-0.17) | 108 | 581 | 0.11 (0.06-0.15) |
|  |  | 100 | 66 | 735 | 0.15 (0.10-0.19) | 50 | 583 | 0.13 (0.07-0.18) |

Note: severe COVID-19 is defined as the earliest of a recorded hospitalisation or critical care admission within 28 days of COVID-19 diagnosis, or death within 100 days of COVID-19 diagnosis.

Cases with <5 events are not displayed.

Supplementary Table S12. Kaplan Meier probability estimates of severe COVID-19 (hospitalisation, critical care admission or death) and death in patients diagnosed with COVID-19, per reported immunosuppressant use status

| **Outcome** | **Status** | **Time (days)** | **All COVID-19 patients (*N=*9,560)** | | | | **White European subpopulation with COVID-19 (*N=*7,274)** | | |
| --- | --- | --- | --- | --- | --- | --- | --- | --- | --- |
|  |  |  | ***N* risk** | **Cumulative event** | **Probability of event occurring within time window (95% CI)** | ***N* risk** | | **Cumulative event** | **Probability of event occurring within time window (95% CI)** |
| **Severe COVID-19** | | | | | | | | |  |
|  | No immunosuppressant use | < 1 | 9275 | 1663 | 0.18 (0.17-0.19) | 7051 | | 1244 | 0.18 (0.17-0.19) |
|  |  | 1 | 7612 | 1798 | 0.19 (0.19-0.20) | 5807 | | 1345 | 0.19 (0.18-0.20) |
|  |  | 7 | 7206 | 2106 | 0.23 (0.22-0.24) | 5504 | | 1573 | 0.22 (0.21-0.23) |
|  |  | 28 | 5587 | 2388 | 0.26 (0.25-0.27) | 4318 | | 1792 | 0.26 (0.25-0.27) |
|  |  | 60 | 3529 | 2392 | 0.26 (0.25-0.27) | 2718 | | 1795 | 0.26 (0.25-0.27) |
|  |  | 100 | 1068 | 2399 | 0.26 (0.26-0.27) | 793 | | 1800 | 0.26 (0.25-0.27) |
|  | Immunosuppressant use | < 1 | 285 | 2487 | 0.31 (0.25-0.36) | 223 | | 1872 | 0.32 (0.26-0.38) |
|  |  | 1 | 197 | 2494 | 0.33 (0.28-0.39) | 151 | | 1879 | 0.35 (0.29-0.41) |
|  |  | 7 | 172 | 2514 | 0.40 (0.34-0.46) | 134 | | 1890 | 0.40 (0.34-0.47) |
|  |  | 28 | 121 | 2534 | 0.48 (0.42-0.53) | 96 | | 1907 | 0.49 (0.41-0.55) |
|  |  | 60 | 78 | 2535 | 0.49 (0.42-0.54) | 65 | | 1908 | 0.49 (0.42-0.55) |
|  |  | 100 | 18 | 2535 | 0.49 (0.42-0.54) | 14 | | 1908 | 0.49 (0.42-0.55) |
| **Death** | | | | | | | | |  |
|  | No immunosuppressant use | < 1 | 9275 | 80 | 0.01 (0.01-0.01) | 7051 | | 64 | 0.01 (0.01-0.01) |
|  |  | 1 | 9195 | 94 | 0.01 (0.01-0.01) | 6987 | | 77 | 0.01 (0.01-0.01) |
|  |  | 7 | 9027 | 264 | 0.03 (0.03-0.03) | 6859 | | 204 | 0.03 (0.03-0.03) |
|  |  | 28 | 7235 | 583 | 0.07 (0.06-0.07) | 5551 | | 455 | 0.07 (0.06-0.07) |
|  |  | 60 | 4794 | 665 | 0.08 (0.07-0.08) | 3645 | | 521 | 0.08 (0.07-0.09) |
|  |  | 100 | 1900 | 707 | 0.09 (0.08-0.10) | 1388 | | 557 | 0.10 (0.09-0.10) |
|  | Immunosuppressant use | < 1 | 285 | 712 | 0.02 (0.00-0.03) | 223 | | 561 | 0.02 (0.00-0.04) |
|  |  | 1 | 280 | 714 | 0.03 (0.01-0.04) | 219 | | 563 | 0.03 (0.01-0.05) |
|  |  | 7 | 273 | 721 | 0.05 (0.02-0.07) | 213 | | 567 | 0.05 (0.02-0.07) |
|  |  | 28 | 210 | 745 | 0.14 (0.10-0.18) | 168 | | 587 | 0.14 (0.09-0.18) |
|  |  | 60 | 142 | 748 | 0.15 (0.11-0.19) | 118 | | 589 | 0.15 (0.10-0.20) |
|  |  | 100 | 61 | 750 | 0.17 (0.12-0.21) | 52 | | 590 | 0.16 (0.11-0.21) |

Supplementary Table S13. Kaplan Meier probability estimates of severe COVID-19 and death in patients diagnosed with COVID-19, per White European polygenic risk score 2 quintile

| **Outcome** | **Quintile** | **Time (days)** | **All COVID-19 patients (*N=*8,453)** | | | **White European subpopulation with COVID-19 (*N=*7,274)** | | |
| --- | --- | --- | --- | --- | --- | --- | --- | --- |
|  |  |  | ***N* risk** | **Cumulative event** | **Probability of event occurring within time window (95% CI)** | ***N* risk** | **Cumulative event** | **Probability of event occurring within time window (95% CI)** |
| **Severe COVID-19** | | | | | | | |  |
|  | 1 | < 1 | 1349 | 231 | 0.17 (0.15-0.19) | 1195 | 198 | 0.17 (0.14-0.19) |
|  |  | 1 | 1118 | 250 | 0.19 (0.16-0.21) | 997 | 214 | 0.18 (0.16-0.20) |
|  |  | 7 | 1066 | 287 | 0.21 (0.19-0.23) | 954 | 245 | 0.21 (0.18-0.23) |
|  |  | 28 | 809 | 323 | 0.24 (0.22-0.27) | 732 | 278 | 0.24 (0.21-0.26) |
|  |  | 60 | 521 | 325 | 0.25 (0.22-0.27) | 469 | 280 | 0.24 (0.21-0.26) |
|  |  | 100 | 160 | 325 | 0.25 (0.22-0.27) | 132 | 280 | 0.24 (0.21-0.26) |
|  | 2 | < 1 | 1499 | 598 | 0.18 (0.16-0.20) | 1320 | 514 | 0.18 (0.16-0.20) |
|  |  | 1 | 1226 | 619 | 0.20 (0.18-0.22) | 1086 | 533 | 0.19 (0.17-0.21) |
|  |  | 7 | 1167 | 660 | 0.22 (0.20-0.24) | 1032 | 571 | 0.22 (0.20-0.24) |
|  |  | 28 | 869 | 707 | 0.26 (0.24-0.28) | 786 | 613 | 0.26 (0.23-0.28) |
|  |  | 60 | 537 | 709 | 0.26 (0.24-0.28) | 487 | 615 | 0.26 (0.23-0.28) |
|  |  | 100 | 158 | 709 | 0.26 (0.24-0.28) | 137 | 615 | 0.26 (0.23-0.28) |
|  | 3 | < 1 | 1634 | 1009 | 0.18 (0.17-0.20) | 1433 | 880 | 0.19 (0.17-0.21) |
|  |  | 1 | 1334 | 1029 | 0.20 (0.18-0.22) | 1168 | 897 | 0.20 (0.18-0.22) |
|  |  | 7 | 1262 | 1090 | 0.23 (0.21-0.25) | 1109 | 946 | 0.23 (0.21-0.25) |
|  |  | 28 | 995 | 1133 | 0.26 (0.24-0.28) | 882 | 987 | 0.26 (0.24-0.28) |
|  |  | 60 | 627 | 1133 | 0.26 (0.24-0.28) | 556 | 987 | 0.26 (0.24-0.28) |
|  |  | 100 | 187 | 1134 | 0.26 (0.24-0.29) | 156 | 988 | 0.26 (0.24-0.29) |
|  | 4 | < 1 | 1802 | 1457 | 0.18 (0.16-0.20) | 1542 | 1273 | 0.19 (0.17-0.20) |
|  |  | 1 | 1479 | 1490 | 0.20 (0.18-0.22) | 1257 | 1301 | 0.20 (0.18-0.22) |
|  |  | 7 | 1398 | 1547 | 0.23 (0.21-0.25) | 1192 | 1345 | 0.23 (0.21-0.25) |
|  |  | 28 | 1074 | 1608 | 0.27 (0.25-0.29) | 931 | 1397 | 0.27 (0.25-0.29) |
|  |  | 60 | 679 | 1608 | 0.27 (0.25-0.29) | 581 | 1397 | 0.27 (0.25-0.29) |
|  |  | 100 | 203 | 1610 | 0.27 (0.25-0.29) | 171 | 1399 | 0.27 (0.25-0.29) |
|  | 5 | < 1 | 2169 | 2015 | 0.19 (0.17-0.20) | 1784 | 1733 | 0.19 (0.17-0.21) |
|  |  | 1 | 1764 | 2050 | 0.20 (0.19-0.22) | 1450 | 1761 | 0.20 (0.18-0.22) |
|  |  | 7 | 1642 | 2145 | 0.25 (0.23-0.27) | 1351 | 1838 | 0.25 (0.23-0.27) |
|  |  | 28 | 1294 | 2222 | 0.29 (0.27-0.30) | 1083 | 1906 | 0.29 (0.27-0.31) |
|  |  | 60 | 826 | 2222 | 0.29 (0.27-0.30) | 690 | 1906 | 0.29 (0.27-0.31) |
|  |  | 100 | 265 | 2224 | 0.29 (0.27-0.31) | 211 | 1908 | 0.29 (0.27-0.31) |
| **Death** | | | | | | | |  |
|  | 1 | < 1 | 1349 | 14 | 0.01 (0.01-0.02) | 1195 | 11 | 0.01 (0.00-0.02) |
|  |  | 1 | 1335 | 14 | 0.01 (0.01-0.02) | 1184 | 11 | 0.01 (0.00-0.02) |
|  |  | 7 | 1320 | 31 | 0.02 (0.02-0.03) | 1171 | 25 | 0.02 (0.01-0.03) |
|  |  | 28 | 1024 | 83 | 0.07 (0.05-0.08) | 914 | 75 | 0.07 (0.05-0.08) |
|  |  | 60 | 681 | 96 | 0.08 (0.06-0.09) | 604 | 86 | 0.08 (0.06-0.10) |
|  |  | 100 | 265 | 100 | 0.09 (0.07-0.10) | 219 | 90 | 0.09 (0.07-0.11) |
|  | 2 | < 1 | 1499 | 114 | 0.01 (0.00-0.01) | 1320 | 102 | 0.01 (0.00-0.01) |
|  |  | 1 | 1485 | 119 | 0.01 (0.01-0.02) | 1308 | 107 | 0.01 (0.01-0.02) |
|  |  | 7 | 1449 | 151 | 0.03 (0.03-0.04) | 1279 | 132 | 0.03 (0.02-0.04) |
|  |  | 28 | 1115 | 205 | 0.07 (0.06-0.09) | 1001 | 184 | 0.08 (0.06-0.09) |
|  |  | 60 | 721 | 217 | 0.09 (0.07-0.10) | 645 | 194 | 0.09 (0.07-0.10) |
|  |  | 100 | 277 | 223 | 0.10 (0.08-0.11) | 239 | 198 | 0.09 (0.08-0.11) |
|  | 3 | < 1 | 1634 | 241 | 0.01 (0.01-0.02) | 1433 | 214 | 0.01 (0.01-0.02) |
|  |  | 1 | 1616 | 243 | 0.01 (0.01-0.02) | 1417 | 216 | 0.01 (0.01-0.02) |
|  |  | 7 | 1587 | 272 | 0.03 (0.02-0.04) | 1390 | 242 | 0.03 (0.02-0.04) |
|  |  | 28 | 1291 | 321 | 0.06 (0.05-0.08) | 1142 | 288 | 0.07 (0.05-0.08) |
|  |  | 60 | 863 | 333 | 0.07 (0.06-0.09) | 761 | 299 | 0.08 (0.06-0.09) |
|  |  | 100 | 334 | 343 | 0.09 (0.07-0.11) | 278 | 309 | 0.10 (0.08-0.12) |
|  | 4 | < 1 | 1802 | 359 | 0.01 (0.01-0.01) | 1542 | 323 | 0.01 (0.00-0.01) |
|  |  | 1 | 1786 | 362 | 0.01 (0.01-0.02) | 1528 | 326 | 0.01 (0.01-0.02) |
|  |  | 7 | 1754 | 397 | 0.03 (0.02-0.04) | 1503 | 353 | 0.03 (0.02-0.04) |
|  |  | 28 | 1398 | 463 | 0.07 (0.06-0.08) | 1224 | 405 | 0.07 (0.05-0.08) |
|  |  | 60 | 930 | 480 | 0.08 (0.07-0.10) | 807 | 422 | 0.08 (0.07-0.09) |
|  |  | 100 | 373 | 490 | 0.10 (0.08-0.12) | 321 | 432 | 0.10 (0.08-0.12) |
|  | 5 | < 1 | 2169 | 508 | 0.01 (0.00-0.01) | 1784 | 447 | 0.01 (0.00-0.01) |
|  |  | 1 | 2151 | 513 | 0.01 (0.01-0.02) | 1769 | 452 | 0.01 (0.01-0.02) |
|  |  | 7 | 2107 | 556 | 0.03 (0.02-0.04) | 1729 | 491 | 0.03 (0.03-0.04) |
|  |  | 28 | 1730 | 637 | 0.07 (0.06-0.08) | 1438 | 562 | 0.08 (0.06-0.09) |
|  |  | 60 | 1151 | 658 | 0.08 (0.07-0.10) | 946 | 581 | 0.09 (0.08-0.10) |
|  |  | 100 | 490 | 668 | 0.09 (0.08-0.11) | 383 | 590 | 0.10 (0.09-0.12) |

Note: severe COVID-19 is defined as the earliest of a recorded hospitalisation or critical care admission within 28 days of COVID-19 diagnosis, or death within 100 days of COVID-19 diagnosis.

Supplementary Table S14. Unadjusted and adjusted (demographics, immunosuppressant use, autoimmune disease and comorbidity count) odds ratios of severe COVID-19 (hospitalisation, critical care admission or death) in patients diagnosed with COVID-19 (*N=*8,325)

|  | **Unadjusted** | | **Adjusted (demographics, immunosuppressant use, autoimmune disease and comorbidity count)** | |
| --- | --- | --- | --- | --- |
| **Risk factor** | **OR (95% CI)** | ***P*-value** | **OR (95% CI)** | ***P*-value** |
| Age (continuous) | 1.11 (1.10-1.12) | <0.001 | 1.11 (1.10-1.11) | <0.001 |
| Sex |  |  |  |  |
| Female | 1 | <0.001 | 1 | <0.001 |
| Male | 1.88 (1.70-2.08) |  | 1.71 (1.53-1.91) |  |
| Ethnicity |  |  |  |  |
| White | 1 | 0.01 | 1 | <0.001 |
| Black | 1.50 (1.14-1.99) |  | 2.07 (1.49-2.86) |  |
| Other | 0.85 (0.69-1.05) |  | 1.18 (0.93-1.49) |  |
| Smoking status |  |  |  |  |
| Never | 1 | <0.001 | 1 | <0.001 |
| Former | 1.65 (1.49-1.84) |  | 1.12 (1.00-1.27) |  |
| Current | 1.84 (1.58-2.14) |  | 1.91 (1.60-2.27) |  |
| Townsend deprivation quintile |  |  |  |  |
| 1 (Least deprived) | 1 | <0.001 | 1 | <0.001 |
| 2 | 0.97 (0.81-1.15) |  | 0.91 (0.75-1.11) |  |
| 3 | 0.94 (0.79-1.11) |  | 0.87 (0.72-1.05) |  |
| 4 | 1.12 (0.95-1.32) |  | 1.10 (0.91-1.33) |  |
| 5 (Most deprived) | 1.51 (1.29-1.76) |  | 1.33 (1.12-1.60) |  |
|  |  |  |  |  |
| BMI, kg/m^2^ |  |  |  |  |
| <18.5 | 2.71 (1.27-5.77) | <0.001 | 2.44 (1.02-5.64) | <0.001 |
| 18.5 to <25 | 1 |  | 1 |  |
| 25 to <30 | 1.47 (1.28-1.68) |  | 1.13 (0.97-1.31) |  |
| 30 to <35 | 1.96 (1.69-2.27) |  | 1.39 (1.18-1.65) |  |
| 35 to <40 | 2.81 (2.30-3.43) |  | 2.1 (1.68-2.64) |  |
| ≥40 | 3.30 (2.50-4.38) |  | 2.6 (1.89-3.59) |  |
| Immunosuppressant use | 2.77 (2.15-3.57) | <0.001 | 1.93 (1.40-2.67) | <0.001 |
| Auto-immune disease | 1.83 (1.48-2.26) | <0.001 | 1.21 (0.93-1.58) | 0.16 |
| CVD | 2.84 (2.49-3.24) | <0.001 | NA | ·· |
| CRD | 1.36 (1.19-1.55) | <0.001 | NA | ·· |
| CKD | 2.09 (1.27-3.44) | 0.01 | NA | ·· |
| Diabetes | 3.31 (2.79-3.92) | <0.001 | NA | ·· |
| Hypertension | 2.54 (2.29-2.82) | <0.001 | NA | ·· |
| CLD | 3.10 (1.41-6.80) | 0.01 | NA | ·· |
| Neurological disease | 2.44 (1.82-3.28) | <0.001 | NA | ·· |
| Comorbidity count^ |  |  |  |  |
| 0 | 1 | <0.001 | 1 | <0.001 |
| 1 | 2.08 (1.86-2.34) |  | 1.35 (1.19-1.53) |  |
| ≥2 | 4.64 (4.05-5.30) |  | 2.03 (1.74-2.37) |  |
| PRS_e2_ quintile* |  |  |  |  |
| 1 | 1 | 0.11 | NA | ·· |
| 2 | 1.08 (0.91-1.29) |  |  |  |
| 3 | 1.10 (0.93-1.30) |  |  |  |
| 4 | 1.13 (0.96-1.34) |  |  |  |
| 5 | 1.23 (1.05-1.44) |  |  |  |

Note: for each patient, the earliest of recorded hospitalisations or critical care admissions within 28 days of COVID-19 diagnosis, or death within 100 days of COVID-19 diagnosis is considered.

Adjusted model included age (as continuous), sex, ethnicity, smoking status, Townsend deprivation quintile, body mass index, immunosuppressant use, auto-immune disease and comorbidity count.

^Comorbidity count is based on the presence of: cardiovascular disease, chronic respiratory disease, chronic kidney disease, diabetes, hypertension, chronic liver disease, neurological disease.

*PRS_e2_ (as continuous) was also modelled in the unadjusted analysis and had a significant *P* value (0.004).

*P* value from the likelihood ratio test for association

BMI, body mass index; CKD, chronic kidney disease; CLD, chronic liver disease; CRD, chronic respiratory disease; CVD, cardiovascular disease; NA, not applicable; PRS_e2_, White European polygenic risk score 2; OR, odds ratio.

Supplementary Table S15. Unadjusted, age-adjusted, clinico-demographic adjusted and clinico-demographic and PRSe_2_ adjusted odds ratios of severe COVID-19 (hospitalisation, critical care admission or death) in the White European subpopulation diagnosed with COVID-19 (*N=*7,206)

| Risk factor | Unadjusted | | Age-adjusted | | Clinico-demographic adjusted | | Clinico-demographic and PRSe_2_ adjusted | |
| --- | --- | --- | --- | --- | --- | --- | --- | --- |
|  | **OR (95% CI)** | ***P*-value** | **OR (95% CI)** | ***P*-value** | **OR (95% CI)** | ***P*-value** | **OR (95% CI)** | ***P*-value** |
| Age (continuous) | 1.12 (1.11-1.13) | <0.001 | NA |  | 1.11 (1.10-1.12) | <0.001 | 1.11 (1.10-1.12) | <0.001 |
| Sex |  |  |  |  |  |  |  |  |
| Female | 1 | <0.001 | 1 | <0.001 | 1 | <0.001 | 1 | <0.001 |
| Male | 1.92 (1.72-2.14) |  | 1.80 (1.60-2.02) |  | 1.72 (1.52-1.95) |  | 1.73 (1.53-1.95) |  |
| Smoking status |  |  |  |  |  |  |  |  |
| Never | 1 | <0.001 | 1 | <0.001 | 1 | <0.001 | 1 | <0.001 |
| Former | 1.73 (1.53-1.92) |  | 1.29 (1.14-1.46) |  | 1.11 (0.97-1.26) |  | 1.10 (0.97-1.25) |  |
| Current | 1.93 (1.63-2.27) |  | 2.17 (1.81-2.60) |  | 1.99 (1.65-2.41) |  | 1.97 (1.63-2.38) |  |
| Townsend deprivation quintile |  |  |  |  |  |  |  |  |
| 1 (Least deprived) | 1 | <0.001 | 1 | <0.001 | 1 | <0.001 | 1 | <0.001 |
| 2 | 0.94 (0.78-1.12) |  | 0.89 (0.73-1.08) |  | 0.89 (0.73-1.09) |  | 0.89 (0.73-1.09) |  |
| 3 | 0.94 (0.79-1.12) |  | 0.91 (0.75-1.10) |  | 0.86 (0.71-1.05) |  | 0.86 (0.71-1.05) |  |
| 4 | 1.14 (0.95-1.35) |  | 1.22 (1.01-1.48) |  | 1.13 (0.93-1.38) |  | 1.13 (0.93-1.37) |  |
| 5 (Most deprived) | 1.51 (1.28-1.78) |  | 1.54 (1.28-1.84) |  | 1.28 (1.06-1.54) |  | 1.28 (1.06-1.54) |  |
|  |  |  |  |  |  |  |  |  |
| BMI, kg/m^2^ |  |  |  |  |  |  |  |  |
| <18.5 | 2.86 (1.23-6.67) | <0.001 | 2.72 (1.06-6.67) | <0.001 | 2.14 (0.79-5.57) | <0.001 | 2.09 (0.77-5.46) | <0.001 |
| 18.5 to <25 | 1 |  | 1 |  | 1 |  | 1 |  |
| 25 to <30 | 1.48 (1.28-1.71) |  | 1.29 (1.11-1.50) |  | 1.13 (0.97-1.33) |  | 1.14 (0.97-1.33) |  |
| 30 to <35 | 2.01 (1.71-2.35) |  | 1.73 (1.46-2.06) |  | 1.44 (1.20-1.72) |  | 1.43 (1.20-1.72) |  |
| 35 to <40 | 2.89 (2.32-3.60) |  | 2.79 (2.20-3.53) |  | 2.23 (1.74-2.87) |  | 2.24 (1.74-2.88) |  |
| ≥40 | 3.12 (2.30-4.22) |  | 3.25 (2.33-4.52) |  | 2.31 (1.62-3.30) |  | 2.33 (1.63-3.32) |  |
| Immunosuppressant use | 2.76 (2.11-3.62) | <0.001 | 2.16 (1.62-2.90) | <0.001 | 1.96 (1.39-2.77) | <0.001 | 1.95 (1.38-2.77) | <0.001 |
| Auto-immune disease | 1.78 (1.43-2.22) | <0.001 | 1.54 (1.21-1.95) | <0.001 | 1.17 (0.88-1.55) | 0.29 | 1.16 (0.87-1.54) | 0.3 |
| CVD | 3.03 (2.63-3.48) | <0.001 | 1.86 (1.6-2.16) | <0.001 | 1.41 (1.20-1.65) | <0.001 | 1.40 (1.19-1.64) | <0.001 |
| CRD | 1.41 (1.23-1.62) | <0.001 | 1.43 (1.23-1.66) | <0.001 | 1.31 (1.12-1.53) | <0.001 | 1.31 (1.12-1.54) | <0.001 |
| CKD | 1.94 (1.11-3.38) | 0.02 | 1.88 (1.01-3.42) | 0.04 | 1.37 (0.71-2.61) | 0.35 | 1.36 (0.70-2.59) | 0.36 |
| Diabetes | 3.54 (2.92-4.30) | <0.001 | 2.49 (2.02-3.08) | <0.001 | 1.68 (1.34-2.10) | <0.001 | 1.70 (1.35-2.12) | <0.001 |
| Hypertension | 2.48 (2.22-2.77) | <0.001 | 1.54 (1.36-1.73) | <0.001 | 1.19 (1.04-1.35) | 0.011 | 1.18 (1.04-1.35) | 0.012 |
| CLD | 3.42 (1.48-7.94) | 0.01 | 3.97 (1.62-9.95) | 0.01 | 3.48 (1.39-8.93) | 0.008 | 3.43 (1.37-8.81) | 0.009 |
| Neurological disease | 2.45 (1.81-3.32) | <0.001 | 2.00 (1.44-2.78) | <0.001 | 1.95 (1.38-2.74) | <0.001 | 1.93 (1.36-2.72) | <0.001 |
| Comorbidity count^ |  |  |  |  |  |  |  |  |
| 0 | 1 | <0.001 | 1 | <0.001 | 1 | <0.001 | 1 | <0.001 |
| 1 | 2.07 (1.83-2.34) |  | 1.48 (1.30-1.69) |  | 1.34 (1.17-1.53) |  | 1.33 (1.16-1.53) |  |
| ≥2 | 4.76 (4.12-5.50) |  | 2.72 (2.33-3.18) |  | 2.09 (1.77-2.46) |  | 2.09 (1.77-2.47) |  |
| PRS_e2_ quintile |  |  |  |  |  |  |  |  |
| 1 | 1 | 0.03 | 1 | 0.01 | 1 | 0.01 | NA | ·· |
| 2 | 1.11 (0.92-1.33) |  | 1.18 (0.97-1.44) |  | 1.15 (0.93-1.41) |  |  |  |
| 3 | 1.15 (0.96-1.37) |  | 1.15 (0.95-1.40) |  | 1.09 (0.89-1.34) |  |  |  |
| 4 | 1.19 (1.00-1.42) |  | 1.30 (1.08-1.58) |  | 1.29 (1.06-1.58) |  |  |  |
| 5 | 1.31 (1.11-1.55) |  | 1.42 (1.18-1.70) |  | 1.37 (1.14-1.66) |  |  |  |

Note: for each patient, the earliest of recorded hospitalisations or critical care admissions within 28 days of COVID-19 diagnosis, or death within 100 days of COVID-19 diagnosis is considered.

Clinico-demographic adjusted model included age (as continuous), sex, smoking status, Townsend deprivation quintile, body mass index, immunosuppressant use, autoimmune disease and comorbidities. In this model, comorbidity count is adjusted for these variables excepting comorbidities.

Clinico-demographic and PRS_e2_ adjusted model included PRS_e2_ (as continuous) in addition to the variables included in the clinico-demographic adjusted model (listed above). In this model, PRS_e2_ (as continuous) had a significant *P* value (<0.001). In this model, comorbidity count is adjusted for PRS_e2_ (as continuous) in addition to the variables included when modelling comorbidity count in the clinico-demographic adjusted model (listed above).

^Comorbidity count is based on the presence of: cardiovascular disease, chronic respiratory disease, chronic kidney disease, diabetes, hypertension, chronic liver disease, neurological disease.

*P* value from the likelihood ratio test for association

BMI, body mass index; CKD, chronic kidney disease; CLD, chronic liver disease; CRD, chronic respiratory disease; CVD, cardiovascular disease; NA, not applicable; OR, odds ratio; PRS_e2_, White European polygenic risk score 2.

Supplementary Table S16: Clinico-demographic and PRS adjusted odds ratios of hospitalized patients diagnosed with COVID-19, with (N=8,325) and without (*N=*7,635) a fatal outcome.

| Risk factor | **Severe COVID-19: hospitalization, critical care admission or death** | | **Severe COVID-19: hospitalization or critical care admission who survived** | |
| --- | --- | --- | --- | --- |
|  | **OR (95% CI)** | ***P* value** | **OR (95% CI)** | ***P* value** |
| Age (continuous) | 1.11 (1.10-1.11) | <0.001 | 1.08 (1.07-1.09) | <0.001 |
| Sex |  |  |  |  |
| Female | 1 | <0.001 | 1 | <0.001 |
| Male | 1.70 (1.52-1.91) |  | 1.56 (1.38-1.77) |  |
| Ethnicity |  |  |  |  |
| White | 1 | <0.001 | 1 | <0.001 |
| Black | 2.21 (1.59-3.06) |  | 2.02 (1.41-2.86) |  |
| Other | 1.10 (0.86-1.39) |  | 1.16 (0.90-1.49) |  |
| Smoking status |  |  |  |  |
| Never | 1 | <0.001 | 1 | <0.001 |
| Former | 1.12 (0.99-1.26) |  | 1.08 (0.94-1.24) |  |
| Current | 1.89 (1.58-2.25) |  | 1.68 (1.38-2.03) |  |
| Townsend deprivation quintile |  |  |  |  |
| 1 (Least deprived) | 1 | <0.001 | 1 | <0.001 |
| 2 | 0.91 (0.75-1.11) |  | 0.96 (0.77-1.19) |  |
| 3 | 0.86 (0.71-1.05) |  | 0.87 (0.70-1.08) |  |
| 4 | 1.10 (0.91-1.32) |  | 1.14 (0.93-1.40) |  |
| 5 (Most deprived) | 1.33 (1.11-1.59) |  | 1.38 (1.13-1.69) |  |
| BMI, kg/m^2^ |  |  |  |  |
| <18.5 | 2.33 (0.96-5.46) | <0.001 | 2.25 (0.81-5.62) | <0.001 |
| 18.5 to <25 | 1 |  | 1 |  |
| 25 to <30 | 1.13 (0.97-1.31) |  | 1.20 (1.02-1.43) |  |
| 30 to <35 | 1.41 (1.19-1.66) |  | 1.52 (1.26-1.83) |  |
| 35 to <40 | 2.11 (1.68-2.65) |  | 2.10 (1.63-2.70) |  |
| ≥40 | 2.51 (1.81-3.48) |  | 2.50 (1.74-3.56) |  |
| Immunosuppressant use | 1.88 (1.36-2.60) | <0.001 | 1.79 (1.25-2.54) | 0.001 |
| Autoimmune disease | 1.22 (0.93-1.59) | 0.148 | 1.31 (0.98-1.75 | 0.07 |
| CVD | 1.32 (1.13-1.53) | <0.001 | 1.32 (1.11-1.56) | 0.002 |
| CRD | 1.27 (1.09-1.47) | 0.002 | 1.31 (1.12-1.54) | 0.001 |
| CKD | 1.49 (0.83-2.64) | 0.176 | 1.49 (0.79-2.71) | 0.21 |
| Diabetes | 1.62 (1.33-1.97) | <0.001 | 1.49 (1.19-1.86) | <0.001 |
| Hypertension | 1.23 (1.09-1.39) | <0.001 | 1.18 (1.03-1.36) | 0.02 |
| CLD | 3.14 (1.31-7.58) | 0.01 | 2.61 (0.93-6.90) | 0.07 |
| Neurological disease | 1.94 (1.38-2.71) | <0.001 | 1.80 (1.23-2.61) | 0.003 |
| Comorbidity count^a^ |  |  |  |  |
| 0 | 1 | <0.001 | 1 | <0.001 |
| 1 | 1.35 (1.19-1.53) |  | 1.30 (1.13-1.49) |  |
| ≥2 | 2.03 (1.74-2.37) |  | 1.92 (1.62-2.27) |  |
| PRS_e2_ quintile |  |  |  |  |
| 1 | 1 | 0.011 | 1 | 0.06 |
| 2 | 1.12 (0.93-1.36) |  | 1.08 (0.87-1.33) |  |
| 3 | 1.08 (0.89-1.30) |  | 1.09 (0.89-1.34) |  |
| 4 | 1.25 (1.04-1.50) |  | 1.20 (0.98-1.47) |  |
| 5 | 1.32 (1.11-1.58) |  | 1.30 (1.07-1.58) |  |

Note: Severe COVID-19: hospitalisation or critical care admission within 28 days of COVID-19 diagnosis, or death within 100 days of COVID-19 diagnosis.

Clinico-demographic adjusted model included age (as continuous), sex, ethnicity, smoking status, Townsend deprivation quintile, body mass index, immunosuppressant use, autoimmune disease and comorbidities. In this model, comorbidity count is adjusted for these variables excepting comorbidities.

Clinico-demographic and PRS_e2_-adjusted model included PRS_e2_ (as continuous) in addition to the variables included in the clinico-demographic adjusted model (listed above). In this model, PRS_e2_ (as continuous) had a significant *P* value (<0.001). In this model, comorbidity count is adjusted for PRS_e2_ (as continuous) in addition to the variables included when modelling comorbidity count in the clinico-demographic adjusted model (listed above).

^a^Count of the following comorbidities: cardiovascular disease, chronic respiratory disease, chronic kidney disease, diabetes, hypertension, chronic liver disease, neurological disease.

*P* value from the overall likelihood ratio test for association.

BMI, body mass index; CKD, chronic kidney disease; CLD, chronic liver disease; CRD, chronic respiratory disease; CVD, cardiovascular disease; NA, not applicable; OR, odds ratio; PRS_e2_, White European polygenic risk score 2.

Supplementary Table S17. Unadjusted and adjusted (demographics, immunosuppressant use, autoimmune disease and comorbidity count) hazard ratios of death in patients diagnosed with COVID-19 (*N=*8,325)

| **Risk factor** | **Unadjusted** | | **Adjusted (demographics, immunosuppressant use, autoimmune disease and comorbidity count)** | |
| --- | --- | --- | --- | --- |
|  | **HR (95% CI)** | ***P*-value** | **HR (95% CI)** | ***P*-value** |
| Age (cubic spline) |  | <0.001 |  |  |
| 55 | 1 |  | 1 | <0.001 |
| 60 | 2.71 (2.09-3.53) |  | 2.55 (1.96-3.31) |  |
| 65 | 6.68 (4.29-10.41) |  | 5.93 (3.80-9.25) |  |
| 70 | 14.09 (8.52-23.33) |  | 11.97 (7.22-19.84) |  |
| 75 | 26.66 (16.39-43.34) |  | 21.82 (13.36-35.65) |  |
| Sex |  |  |  |  |
| Female | 1 | <0.001 | 1 | <0.001 |
| Male | 2.11 (1.79-2.48) |  | 1.72 (1.45-2.04) |  |
| Ethnicity |  |  |  |  |
| White | 1 | 0.01 | 1 | 0.01 |
| Black | 1.46 (0.97-2.19) |  | 2.3 (1.51-3.51) |  |
| Other | 0.57 (0.38-0.85) |  | 0.94 (0.62-1.43) |  |
| Smoking status |  |  |  |  |
| Never | 1 | <0.001 | 1 | <0.001 |
| Former | 1.92 (1.62-2.27) |  | 1.19 (1.00-1.41) |  |
| Current | 1.97 (1.56-2.49) |  | 1.81 (1.42-2.30) |  |
| Townsend deprivation quintile |  |  |  |  |
| 1 (Least deprived) | 1 | 0.01 | 1 | 0.14 |
| 2 | 0.85 (0.64-1.12) |  | 0.78 (0.59-1.03) |  |
| 3 | 0.90 (0.69-1.18) |  | 0.83 (0.63-1.08) |  |
| 4 | 0.96 (0.74-1.25) |  | 0.95 (0.73-1.24) |  |
| 5 (Most deprived) | 1.23 (0.97-1.56) |  | 1.02 (0.80-1.31) |  |
| BMI, kg/m^2^ |  |  |  |  |
| <18.5 | 2.59 (0.96-7.01)1 | <0.001 | 2.35 (0.86-6.39) | 0.01 |
| 18.5 to <25 | 1.23 (1.00-1.53) |  | 0.90 (0.72-1.12) |  |
| 25 to <30 | 1.54 (1.22-1.94) |  | 1.02 (0.80-1.30) |  |
| 30 to <35 | 2.12 (1.59-2.85) |  | 1.47 (1.09-1.99) |  |
| 35 to <40 | 2.34 (1.58-3.47) |  | 1.59 (1.06-2.37) |  |
| ≥40 |  |  |  |  |
| Immunosuppressant use | 2.05 (1.47-2.86) | <0.001 | 1.46 (0.99-2.13) | 0.06 |
| Auto-immune disease | 1.38 (1.01-1.90) | 0.06 | 0.97 (0.67-1.39) | 0.86 |
| CVD | 2.49 (2.09-2.97) | <0.001 | NA | ·· |
| CRD | 1.09 (0.88-1.34) | 0.45 | NA | ·· |
| CKD | 1.37 (0.65-2.88) | 0.44 | NA | ·· |
| Diabetes | 2.86 (2.33-3.51) | <0.001 | NA | ·· |
| Hypertension | 2.73 (2.34-3.19) | <0.001 | NA | ·· |
| CLD | 2.1 (0.79-5.61) | 0.19 | NA | ·· |
| Neurological disease | 1.38 (1.01-1.90) | <0.001 | NA | ·· |
| Comorbidity count^ |  |  |  |  |
| 0 | 1 | <0.001 | 1 | <0.001 |
| 1 | 2.37 (1.95-2.87) |  | 1.36 (1.11-1.65) |  |
| ≥2 | 4.40 (3.61-5.37) |  | 1.68 (1.36-2.08) |  |
| PRS_e2_ quintile* |  |  |  | ·· |
| 1 | 1 | 0.77 | NA |  |
| 2 | 1.13 (0.86-1.48) |  |  |  |
| 3 | 0.99 (0.76-1.30) |  |  |  |
| 4 | 1.11 (0.85-1.43) |  |  |  |
| 5 | 1.10 (0.86-1.42) |  |  |  |

Adjusted model included age (cubic spline with 3 knots), sex, ethnicity, smoking status, Townsend deprivation quintile, body mass index, immunosuppressant use, autoimmune disease and comorbidity count.

^Comorbidity count is based on the presence of: cardiovascular disease, chronic respiratory disease, chronic kidney disease, diabetes, hypertension, chronic liver disease, neurological disease.

*PRS_e2_ (as continuous) was also modelled in the unadjusted analysis and had a non-significant *P* value (0.399).

*P* value from the likelihood ratio test for association.

BMI, body mass index; CKD, chronic kidney disease; CLD, chronic liver disease; CRD, chronic respiratory disease; CVD, cardiovascular disease; HR, hazard ratio; NA, not applicable; PRS_e2_, White European polygenic risk score 2.

Supplementary Table S18. Unadjusted, age-adjusted, clinico-demographic adjusted and clinico-demographic and PRS_e2_ adjusted hazard ratios of death in the White European subpopulation diagnosed with COVID-19 (*N=* 7,206)

| Risk factor | Unadjusted | | Age-adjusted | | Clinico-demographic adjusted | | Clinico-demographic and PRS_e2_ adjusted | |
| --- | --- | --- | --- | --- | --- | --- | --- | --- |
|  | **HR (95% CI)** | ***P*-value** | **HR (95% CI)** | ***P*-value** | **HR (95% CI)** | ***P*-value** | **HR (95% CI)** | ***P*-value** |
| Age (cubic spline) |  |  |  |  |  |  |  |  |
| 55 | 1 | <0.001 | *NA* |  | 1 | <0.001 | 1 | <0.001 |
| 60 | 2.64 (2.00-3.48) |  |  |  | 2.45 (1.86-3.22) |  | 2.45 (1.86-3.23) |  |
| 65 | 6.48 (4.01-10.48) |  |  |  | 5.62 (3.49-9.08) |  | 5.64 (3.49-9.11) |  |
| 70 | 13.94 (8.00-24.28) |  |  |  | 11.49 (6.60-20.00) |  | 11.53 (6.62-20.08) |  |
| 75 | 26.94 (15.73-46.16) |  |  |  | 21.37 (12.46-36.67) |  | 21.46 (12.50-36.84) |  |
| Sex |  |  |  |  |  |  |  |  |
| Female | 1 | <0.001 | 1 | <0.001 | 1 | <0.001 | 1 | <0.001 |
| Male | 2.31 (1.93-2.76) |  | 1.99 (1.67-2.38) |  | 1.83 (1.52-2.19) |  | 1.83 (1.52-2.20) |  |
| Smoking status |  |  |  |  |  |  |  |  |
| Never | 1 | <0.001 | 1 | <0.001 | 1 | <0.001 | 1 | <0.001 |
| Former | 2.01 (1.67-2.41) |  | 1.41 (1.17-1.70) |  | 1.25 (1.04-1.50) |  | 1.24 (1.03-1.50) |  |
| Current | 2.20 (1.72-2.82) |  | 2.26 (1.77-2.90) |  | 1.98 (1.54-2.56) |  | 1.96 (1.52-2.52) |  |
| Townsend deprivation quintile |  |  |  |  |  |  |  |  |
| 1 (Least deprived) | 1 | 0.01 | 1 | 0.01 | 1 | 0.14 | 1 | 0.13 |
| 2 | 0.83 (0.62-1.11) |  | 0.77 (0.58-1.03) |  | 0.77 (0.58-1.03) |  | 0.77 (0.58-1.03) |  |
| 3 | 0.92 (0.70-1.22) |  | 0.88 (0.67-1.16) |  | 0.84 (0.64-1.11) |  | 0.84 (0.63-1.11) |  |
| 4 | 0.96 (0.73-1.27) |  | 1.00 (0.76-1.31) |  | 0.96 (0.73-1.26) |  | 0.96 (0.73-1.26) |  |
| 5 (Most deprived) | 1.31 (1.02-1.68) |  | 1.21 (0.94-1.55) |  | 1.04 (0.81-1.34) |  | 1.04 (0.81-1.34) |  |
|  |  |  |  |  |  |  |  |  |
| BMI, kg/m^2^ |  |  |  |  |  |  |  |  |
| <18.5 | 2.28 (0.72-7.17) | <0.001 | 2.10 (0.67-6.60) | 0.01 | 1.95 (0.62-6.18) | 0.11 | 1.93 (0.61-6.11) | 0.11 |
| 18.5 to <25 | 1 |  | 1 |  | 1 |  | 1 |  |
| 25 to <30 | 1.24 (0.99-1.55) |  | 1.01 (0.81-1.27) |  | 0.87 (0.69-1.10) |  | 0.87 (0.70-1.10) |  |
| 30 to <35 | 1.55 (1.21-1.98) |  | 1.22 (0.95-1.55) |  | 0.98 (0.76-1.27) |  | 0.98 (0.76-1.27) |  |
| 35 to <40 | 2.02 (1.47-2.78) |  | 1.66 (1.21-2.28) |  | 1.33 (0.95-1.85) |  | 1.33 (0.96-1.86) |  |
| ≥40 | 1.80 (1.14-2.84) |  | 1.51 (0.96-2.39) |  | 1.12 (0.70-1.80) |  | 1.11 (0.69-1.79) |  |
| Immunosuppressant use | 1.92 (1.35-2.75) | 0.01 | 1.36 (0.95-1.94) | 0.11 | *NA* | ·· | *NA* | ·· |
| Auto-immune disease | 1.34 (0.96-1.86) | 0.1 | 1.10 (0.79-1.54) | 0.58 | *NA* | ·· | NA | ·· |
| CVD | 2.56 (2.13-3.07) | <0.001 | 1.39 (1.16-1.68) | <0.001 | 1.15 (0.95-1.40) | 0.15 | 1.15 (0.95-1.39) | 0.16 |
| CRD | 1.14 (0.92-1.42) | 0.24 | 1.10 (0.88-1.36) | 0.42 | *NA* | ·· | NA | ·· |
| CKD | 0.90 (0.34-2.42) | 0.84 | 0.81 (0.30-2.17) | 0.67 | *NA* | ·· | NA | ·· |
| Diabetes | 2.93 (2.34-3.68) | <0.001 | 1.84 (1.47-2.31) | <0.001 | 1.44 (1.13-1.83) | 0.01 | 1.44 (1.13-1.84) | 0.01 |
| Hypertension | 2.59 (2.20-3.05) | <0.001 | 1.45 (1.22-1.71) | <0.001 | 1.26 (1.05-1.50) | 0.01 | 1.26 (1.05-1.50) | 0.01 |
| CLD | 2.4 (0.9-6.42) | 0.13 | 2.71 (1.01-7.27) | 0.09 | 2.62 (0.97-7.04) | 0.1 | 2.55 (0.95-6.86) | 0.11 |
| Neurological disease | 1.96 (1.32-2.91) | 0.01 | 1.35 (0.91-2.00) | 0.15 | *NA* | ·· | NA | ·· |
| Comorbidity count^ |  |  |  |  |  |  |  |  |
| 0 | 1 | <0.001 | 1 | <0.001 | 1 | <0.001 | 1 | <0.001 |
| 1 | 2.27 (1.86-2.78) |  | 1.42 (1.16-1.75) |  | 1.34 (1.09-1.65) |  | 1.33 (1.08-2.14) |  |
| ≥2 | 4.25 (3.45-5.23) |  | 1.98 (1.60-2.46) |  | 1.71 (1.37-2.14) |  | 1.71 (1.37-2.14) |  |
| PRS_e2_ quintile |  |  |  |  |  |  |  |  |
| 1 | 1 | 0.64 | 1 | 0.4 | 1 | 0.56 | NA | ·· |
| 2 | 1.11 (0.84-1.48) |  | 1.14 (0.86-1.52) |  | 1.11 (0.83-1.47) |  |  |  |
| 3 | 1.03 (0.77-1.36) |  | 1.01 (0.76-1.34) |  | 0.97 (0.73-1.29) |  |  |  |
| 4 | 1.07 (0.81-1.41) |  | 1.13 (0.86-1.49) |  | 1.10 (0.84-1.46) |  |  |  |
| 5 | 1.20 (0.92-1.56) |  | 1.24 (0.96-1.62) |  | 1.18 (0.90-1.53) |  |  |  |

Note: comorbidity count in clinico-demographic adjusted model is adjusted for age (as cubic spline), sex, ethnicity, smoking status, Townsend deprivation quintile, body mass index.

Clinico-demographic adjusted model included age (as cubic spline), sex, smoking status, Townsend deprivation quintile, body mass index, CVD, diabetes and hypertension. In this model, comorbidity count is adjusted for these variables excepting for CVD, diabetes and hypertension.

Clinico-demographic and PRS adjusted model included PRS (as continuous) in addition to the variables included in the clinico-demographic adjusted model (listed above). In this model, PRS_e2_ (as continuous) had a significant *P* value (0.076). In this model, comorbidity count is adjusted for for PRS_e2_ (as continuous) in addition to the variables included when modelling comorbidity count in the clinico-demographic adjusted model (listed above).

^Comorbidity count is based on the presence of: cardiovascular disease, chronic respiratory disease, chronic kidney disease, diabetes, hypertension, chronic liver disease, neurological disease.

*P* value from the likelihood ratio test for association.

BMI, body mass index; CKD, chronic kidney disease; CLD, chronic liver disease; CRD, chronic respiratory disease; CVD, cardiovascular disease; HR, hazard ratio; NA, not applicable; PRS_e2_, White European polygenic risk score 2.

### Supplementary Table S19: Information about SNPs in PRS_e2_.

| **SNP** | **CHR** | **BP** | ***P*-value** | ***OR*** | **Gene** | **OS** | **Phenotype** | **Cases (*N*)** | **Controls (*N*)** | **OS *P*-value** |
| --- | --- | --- | --- | --- | --- | --- | --- | --- | --- | --- |
| rs78933805 | 1 | 3744126 | 1.36 x 10^-3^ | -0.13 | CEP104 | 32404885 | COVID-19 vs. Population (transethnic - release 5) | 36,590 | 1,668,938 | 3.54 x 10^-6^ |
| rs9287218 | 1 | 237277098 | 1.30 x 10^-3^ | -0.09 | RYR2 | 32404885 | Very severe respiratory confirmed COVID-19 vs. Population (transethnic - release 5) | 5,870 | 1,155,203 | 3.70 x 10^-6^ |
| rs340842 | 1 | 214123368 | 1.55 x 10^-3^ | -0.08 | PROX1-AS1 | 32404885 | Very severe respiratory confirmed COVID-19 vs. Population (transethnic - release 5) | 5,870 | 1,155,203 | 9.57 x 10^-7^ |
| rs6675468 | 1 | 155040654 | 1.91 x 10^-4^ | -0.08 | EFNA4; EFNA3 | 32404885 | Hospitalized COVID-19 vs. Population (European - release 5) | 9,986 | 1,877,672 | 1.84 x 10^-7^ |
| rs4971066 | 1 | 155105882 | 1.37 x 10^-4^ | -0.06 | SLC50A1; EFNA1 | 32404885 | COVID-19 vs. Population (transethnic - release 5) | 36,590 | 1,668,938 | 1.45 x 10^-6^ |
| rs74135457 | 1 | 188497907 | 0.49 | -0.04 | RP11-669M2.1 | 32404885 | Very severe respiratory confirmed COVID-19 vs. Population (transethnic - release 5) | 5,870 | 1,155,203 | 6.36 x 10^-7^ |
| rs357225 | 1 | 71965065 | 0.01 | -0.03 | NEGR1 | 32404885 | COVID-19 vs. Population (transethnic - release 5) | 36,590 | 1,668,938 | 1.15 x 10^-6^ |
| rs2224986 | 1 | 152684866 | 0.80 | -0.01 | LCE4A | 32404885 | Hospitalized COVID-19 vs. Non-Hospitalized COVID-19 (transethnic - release 4) | 2,430 | 8,478 | 8.07 x 10^-6^ |
| rs701232 | 1 | 233791469 | 0.65 | 0.01 | KCNK1 | 32404885 | Hospitalized COVID-19 vs. Non-Hospitalized COVID-19 (transethnic - release 5) | 3,961 | 10,538 | 5.66 x 10^-6^ |
| rs1151676 | 1 | 247803638 | 0.52 | 0.01 | RP11-978I15.10; RP11-634B7.4 | 32404885 | Hospitalized COVID-19 vs. Non-Hospitalized COVID-19 (transethnic - release 5) | 3,961 | 10,538 | 6.93 x 10^-6^ |
| rs10399665 | 1 | 8407287 | 0.46 | 0.01 | SLC45A1 | 32404885 | Hospitalized COVID-19 vs. Non-Hospitalized COVID-19 (transethnic - release 5) | 3,961 | 10,538 | 1.79 x 10^-6^ |
| rs10926975 | 1 | 243397782 | 0.20 | 0.02 | CEP170; AC092782.1 | 32404885 | Hospitalized COVID-19 vs. Population (European - release 5) | 9,986 | 1,877,672 | 8.66 x 10^-6^ |
| rs56229346 | 1 | 186849313 | 0.52 | 0.04 | PLA2G4A | 32404885 | Hospitalized COVID-19 vs. Non-Hospitalized COVID-19 (transethnic - release 4) | 2,430 | 8,478 | 4.01 x 10^-6^ |
| rs4076440 | 1 | 9690476 | 0.01 | 0.06 | - | 32404885 | Very severe respiratory confirmed COVID-19 vs. Population (transethnic - release 5) | 5,870 | 1,155,203 | 1.47 x 10^-6^ |
| rs72859229 | 1 | 7214655 | 0.01 | 0.13 | CAMTA1 | 32404885 | Hospitalized COVID-19 vs. Population (European - release 5) | 9,986 | 1,877,672 | 4.41 x 10^-7^ |
| rs4670751 | 2 | 37889700 | 3.75 x 10^-3^ | -0.05 | CDC42EP3 | 32404885 | Hospitalized COVID-19 vs. Population (European - release 5) | 9,986 | 1,877,672 | 9.65 x 10^-6^ |
| rs7575938 | 2 | 166910677 | 1.04 x 10^-4^ | -0.05 | SCN1A; AC010127.3 | 32404885 | Hospitalized COVID-19 vs. Population (European - release 5) | 9,986 | 1,877,672 | 5.87 x 10^-6^ |
| rs7572273 | 2 | 144174295 | 0.05 | -0.04 | ARHGAP15; AC096558.1;  RP11-570L15.2; | 32404885 | Very severe respiratory confirmed COVID-19 vs. Population (transethnic - release 5) | 5,870 | 1,155,203 | 2.07 x 10^-7^ |
| rs45622439 | 2 | 60741520 | 1.67 x 10^-3^ | -0.04 | BCL11A | 32404885 | Hospitalized COVID-19 vs. Population (European - release 5) | 9,986 | 1,877,672 | 5.20 x 10^-6^ |
| rs79550812 | 2 | 182698659 | 0.66 | -0.02 | - | 32404885 | Hospitalized COVID-19 vs. Non-Hospitalized COVID-19 (transethnic - release 4) | 2,430 | 8,478 | 4.85 x 10^-6^ |
| rs6543668 | 2 | 32923972 | 0.30 | -0.01 | TTC27 | 32404885 | Hospitalized COVID-19 vs. Non-Hospitalized COVID-19 (transethnic - release 5) | 3,961 | 10,538 | 1.95 x 10^-6^ |
| rs77695931 | 2 | 207133409 | 0.74 | -0.01 | - | 32404885 | Hospitalized COVID-19 vs. Non-Hospitalized COVID-19 (transethnic - release 4) | 2,430 | 8,478 | 8.84 x 10^-6^ |
| rs55917944 | 2 | 66815140 | 0.74 | -0.01 | AC007392.3 | 32404885 | Very severe respiratory confirmed COVID-19 vs. Population (transethnic - release 5) | 5,870 | 1,155,203 | 7.06 x 10^-6^ |
| rs73980984 | 2 | 188814713 | 0.03 | 0.03 | LINC01090 | 32404885 | Very severe respiratory confirmed COVID-19 vs. Population (transethnic - release 5) | 5,870 | 1,155,203 | 7.31 x 10^-6^ |
| rs113488799 | 2 | 229773795 | 0.01 | 0.04 | PID1 | 32404885 | Very severe respiratory confirmed COVID-19 vs. Population (transethnic - release 5) | 5,870 | 1,155,203 | 2.31 x 10^-6^ |
| rs7578040 | 2 | 240685455 | 0.08 | 0.06 | AC093802.1 | 32404885 | COVID-19 vs. Population (transethnic - release 5) | 36,590 | 1,668,938 | 1.71 x 10^-6^ |
| rs6433794 | 2 | 180394563 | 0.01 | 0.07 | ZNF385B | 32404885 | COVID-19 vs. Population (transethnic - release 5) | 36,590 | 1,668,938 | 5.71 x 10^-6^ |
| rs12618270 | 2 | 240632655 | 0.05 | 0.08 | - | 32404885 | Hospitalized COVID-19 vs. Population (European - release 5) | 9,986 | 1,877,672 | 1.60 x 10^-6^ |
| rs140228296 | 2 | 208722351 | 0.02 | 0.11 | PLEKHM3 | 32404885 | Hospitalized COVID-19 vs. Population (European - release 5) | 9,986 | 1,877,672 | 3.71 x 10^-6^ |
| rs150800548 | 2 | 233545637 | 1.57 x 10^-4^ | 0.16 | EFHD1; snoU13 | 32404885 | COVID-19 vs. Population (transethnic - release 5) | 36,590 | 1,668,938 | 8.28 x 10^-6^ |
| rs76896797 | 3 | 45838178 | 1.01 x 10^-4^ | -0.08 | SLC6A20 | 32404885 | COVID-19 vs. Population (transethnic - release 5) | 36,590 | 1,668,938 | 7.05 x 10^-8^ |
| rs73863360 | 3 | 101316837 | 1.44 x 10^-7^ | -0.07 | PCNP; ZBTB11; RPL24; SENP7; NFKBIZ; NXPE3; TRMT10C; CEP97; RP11-454H13. | 32404885 | COVID-19 vs. Population (transethnic - release 5) | 36,590 | 1,668,938 | 5.89 x 10^-10^ |
| rs73212324 | 3 | 197107352 | 8.17 x 10^-4^ | -0.07 | - | 32404885 | Very severe respiratory confirmed COVID-19 vs. Population (transethnic - release 5) | 5,870 | 1,155,203 | 8.53 x 10^-7^ |
| rs112515687 | 3 | 46364186 | 6.94 x 10^-6^ | -0.05 | CCR5; CCR2; CCR3 | 32404885 | Hospitalized COVID-19 vs. Population (European - release 5) | 9,986 | 1,877,672 | 2.65 x 10^-7^ |
| rs2173640 | 3 | 46100496 | 3.98 x 10^-3^ | -0.03 | - | 32404885 | Hospitalized COVID-19 vs. Population (European - release 5) | 9,986 | 1,877,672 | 1.22 x 10^-6^ |
| rs76488148 | 3 | 148718087 | 0.55 | -0.02 | GYG1 | 32404885 | Hospitalized COVID-19 vs. Non-Hospitalized COVID-19 (transethnic - release 4) | 2,430 | 8,478 | 6.82 x 10^-6^ |
| rs1948879 | 3 | 51935719 | 0.89 | 1.79 x 10^-3^ | IQCF1 | 32404885 | Very severe respiratory confirmed COVID-19 vs. Population (transethnic - release 5) | 5,870 | 1,155,203 | 1.40 x 10^-6^ |
| rs13062942 | 3 | 62936766 | 0.80 | 3.23 x 10^-3^ | LINC00698 | 32404885 | Hospitalized COVID-19 vs. Non-Hospitalized COVID-19 (transethnic - release 4) | 2,430 | 8,478 | 4.14 x 10^-6^ |
| rs4234659 | 3 | 117474384 | 0.22 | 0.01 | RP11-384F7.2; LSAMP | 32404885 | Very severe respiratory confirmed COVID-19 vs. Population (transethnic - release 5) | 5,870 | 1,155,203 | 9.43 x 10^-6^ |
| rs79835427 | 3 | 137926476 | 0.44 | 0.03 | ARMC8 | 32404885 | Hospitalized COVID-19 vs. Non-Hospitalized COVID-19 (transethnic - release 4) | 2,430 | 8,478 | 8.11 x 10^-6^ |
| rs9311380 | 3 | 45996047 | 4.45 x 10^-4^ | 0.04 | FYCO1 | 32404885 | Hospitalized COVID-19 vs. Population (European - release 5) | 9,986 | 1,877,672 | 2.55 x 10^-8^ |
| rs7629838 | 3 | 146406981 | 5.46 x 10^-4^ | 0.04 | - | 32404885 | COVID-19 vs. Population (transethnic - release 5) | 36,590 | 1,668,938 | 6.82 x 10^-6^ |
| rs2271616 | 3 | 45838013 | 2.55 x 10^-15^ | 0.14 | SLC6A20 | 32404885 | COVID-19 vs. Population (transethnic - release 5) | 36,590 | 1,668,938 | 9.63 x 10^-24^ |
| rs35508621 | 3 | 45880481 | 1.92 x 10^-22^ | 0.19 | LZTFL1; SLC6A20 | 32404885 | Hospitalized COVID-19 vs. Non-Hospitalized COVID-19 (transethnic - release 4) | 2,430 | 8,478 | 6.64 x 10^-8^ |
| rs141045534 | 3 | 45637109 | 1.33 x 10^-7^ | 0.26 | LIMD1; SLC6A20; SACM1L; AC099539.1 | 32404885 | COVID-19 vs. Population (transethnic - release 5) | 36,590 | 1,668,938 | 1.36 x 10^-9^ |
| rs72682662 | 4 | 122926252 | 1.22 x 10^-3^ | 0.09 | - | 32404885 | COVID-19 vs. Population (transethnic - release 5) | 36,590 | 1,668,938 | 1.33 x 10^-6^ |
| rs173090 | 5 | 13954951 | 8.24 x 10^-5^ | -0.05 | - | 32404885 | COVID-19 vs. Population (transethnic - release 5) | 36,590 | 1,668,938 | 5.79 x 10^-7^ |
| rs11740360 | 5 | 94285653 | 6.50 x 10^-4^ | -0.04 | MCTP1 | 32404885 | Hospitalized COVID-19 vs. Population (European - release 5) | 9,986 | 1,877,672 | 3.95 x 10^-6^ |
| rs1834967 | 5 | 38366133 | 0.01 | -0.04 | EGFLAM | 32404885 | Hospitalized COVID-19 vs. Population (European - release 5) | 9,986 | 1,877,672 | 4.27 x 10^-6^ |
| rs13168774 | 5 | 131740656 | 0.05 | -0.03 | - | 32404885 | Very severe respiratory confirmed COVID-19 vs. Population (transethnic - release 5) | 5,870 | 1,155,203 | 7.83 x 10^-6^ |
| rs6867867 | 5 | 54092505 | 0.44 | -0.02 | - | 32404885 | Very severe respiratory confirmed COVID-19 vs. Population (transethnic - release 5) | 5,870 | 1,155,203 | 5.00 x 10^-6^ |
| rs12519258 | 5 | 125762737 | 0.21 | -0.02 | GRAMD3 | 32404885 | Hospitalized COVID-19 vs. Population (European - release 5) | 9,986 | 1,877,672 | 3.48 x 10^-6^ |
| rs114776680 | 5 | 124682392 | 0.96 | -1.80 x 10^-3^ | RN7SKP117 | 32404885 | Hospitalized COVID-19 vs. Non-Hospitalized COVID-19 (transethnic - release 4) | 2,430 | 8,478 | 8.63 x 10^-6^ |
| rs4478338 | 5 | 169590905 | 0.93 | 1.71 x 10^-3^ | - | 32404885 | Hospitalized COVID-19 vs. Non-Hospitalized COVID-19 (transethnic - release 4) | 2,430 | 8,478 | 3.00 x 10^-6^ |
| rs62366701 | 5 | 91803050 | 0.05 | 0.03 | RP11-348J24.2; RP11-133F8.2 | 32404885 | COVID-19 vs. Population (transethnic - release 5) | 36,590 | 1,668,938 | 1.45 x 10^-6^ |
| rs141623490 | 5 | 91686872 | 0.17 | 0.07 | RP11-348J24.2 | 32404885 | Hospitalized COVID-19 vs. Population (European - release 5) | 9,986 | 1,877,672 | 5.52 x 10^-6^ |
| rs75644220 | 5 | 172453639 | 8.21 x 10^-5^ | 0.13 | ATP6V0E1 | 32404885 | COVID-19 vs. Population (transethnic - release 5) | 36,590 | 1,668,938 | 3.76 x 10^-6^ |
| rs2046253 | 5 | 17191271 | 5.99 x 10^-5^ | 0.15 | AC091878.1; BASP1 | 32404885 | COVID-19 vs. Population (transethnic - release 5) | 36,590 | 1,668,938 | 7.15 x 10^-6^ |
| rs28746965 | 6 | 32653136 | 0.15 | -0.02 | - | 32404885 | Very severe respiratory confirmed COVID-19 vs. Population (transethnic - release 5) | 5,870 | 1,155,203 | 4.65 x 10^-6^ |
| rs1264701 | 6 | 30066358 | 0.03 | 0.03 | TRIM31; AL671859.1; CR753815.1 | 32404885 | Very severe respiratory confirmed COVID-19 vs. Population (transethnic - release 5) | 5,870 | 1,155,203 | 4.54 x 10^-7^ |
| rs111837807 | 6 | 31121232 | 1.65 x 10^-5^ | 0.08 | CCHCR1; AL662833.3; CR753819.1; CR847794.2; CR759815.1; AL773544.3 | 32404885 | COVID-19 vs. Population (transethnic - release 5) | 36,590 | 1,668,938 | 1.60 x 10^-7^ |
| rs1886814 | 6 | 41502683 | 0.01 | 0.08 | RP11-328M4.2 | 32404885 | COVID-19 vs. Population (transethnic - release 5) | 36,590 | 1,668,938 | 1.44 x 10^-6^ |
| rs76828289 | 6 | 93866536 | 0.02 | 0.09 | - | 32404885 | Hospitalized COVID-19 vs. Population (European - release 5) | 9,986 | 1,877,672 | 5.63 x 10^-6^ |
| rs140434804 | 6 | 123663895 | 1.30 x 10^-5^ | 0.12 | TRDN | 32404885 | COVID-19 vs. Population (transethnic - release 5) | 36,590 | 1,668,938 | 2.60 x 10^-6^ |
| rs6967210 | 7 | 152960930 | 0.20 | -0.07 | - | 32404885 | Hospitalized COVID-19 vs. Non-Hospitalized COVID-19 (transethnic - release 4) | 2,430 | 8,478 | 6.63 x 10^-6^ |
| rs17181356 | 7 | 139666054 | 0.13 | -0.03 | TBXAS1 | 32404885 | Very severe respiratory confirmed COVID-19 vs. Population (transethnic - release 5) | 5,870 | 1,155,203 | 9.60 x 10^-6^ |
| rs622568 | 7 | 54647894 | 0.15 | 0.03 | GS1-18A18.2 | 32404885 | Hospitalized COVID-19 vs. Population (European - release 5) | 9,986 | 1,877,672 | 3.64 x 10^-9^ |
| rs138955473 | 7 | 24892842 | 0.51 | 0.03 | OSBPL3 | 32404885 | Hospitalized COVID-19 vs. Non-Hospitalized COVID-19 (transethnic - release 4) | 2,430 | 8,478 | 2.17 x 10^-6^ |
| rs2237698 | 7 | 107607902 | 0.03 | 0.04 | LAMB1 | 32404885 | Very severe respiratory confirmed COVID-19 vs. Population (transethnic - release 5) | 5,870 | 1,155,203 | 5.72 x 10^-7^ |
| rs2897075 | 7 | 99630342 | 4.25 x 10^-4^ | 0.04 | ZKSCAN1 | 32404885 | Hospitalized COVID-19 vs. Population (European - release 5) | 9,986 | 1,877,672 | 5.84 x 10^-6^ |
| rs7786241 | 7 | 136792018 | 9.83 x 10^-4^ | 0.06 | hsa-mir-490 | 32404885 | COVID-19 vs. Population (transethnic - release 5) | 36,590 | 1,668,938 | 1.82 x 10^-6^ |
| rs117012139 | 7 | 24220029 | 0.21 | 0.07 | - | 32404885 | Hospitalized COVID-19 vs. Non-Hospitalized COVID-19 (transethnic - release 4) | 2,430 | 8,478 | 9.42 x 10^-7^ |
| rs139907321 | 7 | 46396840 | 0.08 | 0.10 | - | 32404885 | Hospitalized COVID-19 vs. Population (European - release 5) | 9,986 | 1,877,672 | 3.51 x 10^-6^ |
| rs59071057 | 8 | 22057059 | 0.10 | -0.06 | BMP1 | 32404885 | Very severe respiratory confirmed COVID-19 vs. Population (transethnic - release 5) | 5,870 | 1,155,203 | 6.43 x 10^-6^ |
| rs28681155 | 8 | 59917143 | 0.11 | -0.03 | TOX | 32404885 | Very severe respiratory confirmed COVID-19 vs. Population (transethnic - release 5) | 5,870 | 1,155,203 | 5.24 x 10^-6^ |
| rs71521337 | 8 | 29318703 | 0.77 | 0.01 | - | 32404885 | Hospitalized COVID-19 vs. Population (European - release 5) | 9,986 | 1,877,672 | 3.76 x 10^-6^ |
| rs11777988 | 8 | 101370866 | 0.13 | 0.02 | - | 32404885 | Very severe respiratory confirmed COVID-19 vs. Population (transethnic - release 5) | 5,870 | 1,155,203 | 3.84 x 10^-6^ |
| rs75444665 | 8 | 26820407 | 4.04 x 10^-3^ | 0.08 | - | 32404885 | Hospitalized COVID-19 vs. Population (European - release 5) | 9,986 | 1,877,672 | 2.87 x 10^-6^ |
| rs4366152 | 9 | 117564875 | 0.04 | 0.03 | TNFSF15 | 32404885 | Very severe respiratory confirmed COVID-19 vs. Population (transethnic - release 5) | 5,870 | 1,155,203 | 6.99 x 10^-7^ |
| rs8176719 | 9 | 136132908 | 1.58 x 10^-15^ | 0.10 | ABO; RP11-430N14.4 | 32404885 | COVID-19 vs. Population (transethnic - release 5) | 36,590 | 1,668,938 | 1.36 x 10^-24^ |
| rs149181677 | 9 | 136296530 | 2.11 x 10^-8^ | 0.15 | ADAMTS13; SURF1; SURF2; SURF4; MED22; RPL7A | 32404885 | COVID-19 vs. Population (transethnic - release 5) | 36,590 | 1,668,938 | 1.13 x 10^-9^ |
| rs3940961 | 10 | 21568617 | 0.27 | 0.02 | - | 32404885 | Hospitalized COVID-19 vs. Population (European - release 5) | 9,986 | 1,877,672 | 8.89 x 10^-6^ |
| rs72774103 | 10 | 5868476 | 0.27 | 0.05 | GDI2 | 32404885 | Hospitalized COVID-19 vs. Non-Hospitalized COVID-19 (transethnic - release 4) | 2,430 | 8,478 | 4.70 x 10^-6^ |
| rs11016442 | 10 | 130500820 | 2.18 x 10^-4^ | 0.06 | - | 32404885 | COVID-19 vs. Population (transethnic - release 5) | 36,590 | 1,668,938 | 7.53 x 10^-6^ |
| rs61622878 | 10 | 133144402 | 1.40 x 10^-4^ | 0.08 | - | 32404885 | Hospitalized COVID-19 vs. Population (European - release 5) | 9,986 | 1,877,672 | 6.44 x 10^-6^ |
| rs78594643 | 11 | 22828273 | 0.16 | -0.05 | GAS2 | 32404885 | Very severe respiratory confirmed COVID-19 vs. Population (transethnic - release 5) | 5,870 | 1,155,203 | 5.58 x 10^-6^ |
| rs2000819 | 11 | 88588469 | 0.01 | -0.03 | GRM5 | 32404885 | Very severe respiratory confirmed COVID-19 vs. Population (transethnic - release 5) | 5,870 | 1,155,203 | 4.52 x 10^-6^ |
| rs560798 | 11 | 88082520 | 0.01 | -0.03 | - | 32404885 | Very severe respiratory confirmed COVID-19 vs. Population (transethnic - release 5) | 5,870 | 1,155,203 | 3.74 x 10^-6^ |
| rs150298847 | 11 | 120541347 | 0.70 | -0.02 | GRIK4 | 32404885 | Hospitalized COVID-19 vs. Non-Hospitalized COVID-19 (transethnic - release 4) | 2,430 | 8,478 | 2.06 x 10^-7^ |
| rs35189111 | 11 | 102583925 | 0.72 | -0.01 | MMP8 | 32404885 | Hospitalized COVID-19 vs. Population (European - release 5) | 9,986 | 1,877,672 | 8.92 x 10^-6^ |
| rs7126055 | 11 | 28390511 | 0.40 | 0.03 | RP11-22P4.1 | 32404885 | Very severe respiratory confirmed COVID-19 vs. Population (transethnic - release 5) | 5,870 | 1,155,203 | 9.75 x 10^-6^ |
| rs34123181 | 11 | 129894920 | 9.94 x 10^-4^ | 0.11 | - | 32404885 | COVID-19 vs. Population (transethnic - release 5) | 36,590 | 1,668,938 | 3.83 x 10^-6^ |
| rs58912259 | 12 | 19816636 | 0.01 | -0.03 | AEBP2 | 32404885 | COVID-19 vs. Population (transethnic - release 5) | 36,590 | 1,668,938 | 9.60 x 10^-6^ |
| rs4505101 | 12 | 28987480 | 0.07 | -0.02 | - | 32404885 | Hospitalized COVID-19 vs. Population (European - release 5) | 9,986 | 1,877,672 | 6.48 x 10^-6^ |
| rs10746119 | 12 | 108588927 | 0.44 | -0.01 | WSCD2 | 32404885 | Very severe respiratory confirmed COVID-19 vs. Population (transethnic - release 5) | 5,870 | 1,155,203 | 8.81 x 10^-6^ |
| rs12825787 | 12 | 68095307 | 0.39 | 0.01 | - | 32404885 | Hospitalized COVID-19 vs. Population (European - release 5) | 9,986 | 1,877,672 | 3.12 x 10^-6^ |
| rs1859330 | 12 | 113376388 | 1.26 x 10^-9^ | 0.07 | OAS3; OAS1; OAS2; RP1-71H24.1 | 32404885 | COVID-19 vs. Population (transethnic - release 5) | 36,590 | 1,668,938 | 9.37 x 10^-10^ |
| rs7487474 | 12 | 56904025 | 3.72 x 10^-5^ | 0.09 | RP11-153M3.1 | 32404885 | COVID-19 vs. Population (transethnic - release 5) | 36,590 | 1,668,938 | 1.11 x 10^-6^ |
| rs74886300 | 12 | 114993428 | 2.08 x 10^-3^ | 0.18 | AC069240.1 | 32404885 | COVID-19 vs. Population (transethnic - release 5) | 36,590 | 1,668,938 | 8.81 x 10^-6^ |
| rs9524378 | 13 | 94879992 | 6.34 x 10^-6^ | -0.05 | GPC6 | 32404885 | COVID-19 vs. Population (transethnic - release 5) | 36,590 | 1,668,938 | 2.85 x 10^-6^ |
| rs67088066 | 13 | 34857789 | 0.40 | 0.01 | - | 32404885 | Hospitalized COVID-19 vs. Population (European - release 5) | 9,986 | 1,877,672 | 7.70 x 10^-6^ |
| rs12876544 | 13 | 22392842 | 0.31 | 0.01 | - | 32404885 | Very severe respiratory confirmed COVID-19 vs. Population (transethnic - release 5) | 5,870 | 1,155,203 | 3.14 x 10^-6^ |
| rs3742238 | 13 | 113540425 | 3.17 x 10^-3^ | 0.04 | ATP11A; AL356740.1 | 32404885 | Very severe respiratory confirmed COVID-19 vs. Population (transethnic - release 5) | 5,870 | 1,155,203 | 2.17 x 10^-6^ |
| rs12894130 | 14 | 28734655 | 2.99 x 10^-6^ | 0.06 | BNIP3P1 | 32404885 | COVID-19 vs. Population (transethnic - release 5) | 36,590 | 1,668,938 | 5.59 x 10^-6^ |
| rs1998464 | 14 | 27535815 | 0.04 | 0.08 | RP11-384J4.2 | 32404885 | Hospitalized COVID-19 vs. Population (European - release 5) | 9,986 | 1,877,672 | 8.34 x 10^-6^ |
| rs149126344 | 14 | 66718841 | 3.04 x 10^-3^ | 0.11 | RP11-72M17.1 | 32404885 | COVID-19 vs. Population (transethnic - release 5) | 36,590 | 1,668,938 | 1.69 x 10^-6^ |
| rs2580926 | 15 | 49533127 | 0.01 | -0.03 | GALK2 | 32404885 | Very severe respiratory confirmed COVID-19 vs. Population (transethnic - release 5) | 5,870 | 1,155,203 | 2.43 x 10^-6^ |
| rs77055952 | 15 | 45858905 | 0.04 | 0.06 | RP11-96O20.2; DPPA5P2; HMGN2P46 | 32404885 | Hospitalized COVID-19 vs. Non-Hospitalized COVID-19 (transethnic - release 4) | 2,430 | 8,478 | 5.60 x 10^-6^ |
| rs9939750 | 16 | 54550864 | 9.23 x 10^-4^ | -0.04 | - | 32404885 | Very severe respiratory confirmed COVID-19 vs. Population (transethnic - release 5) | 5,870 | 1,155,203 | 9.80 x 10^-6^ |
| rs9922503 | 16 | 78250055 | 9.00 x 10^-6^ | 0.06 | WWOX | 32404885 | COVID-19 vs. Population (transethnic - release 5) | 36,590 | 1,668,938 | 3.42 x 10^-6^ |
| rs555139435 | 16 | 52380471 | 0.07 | 0.08 | - | 32404885 | COVID-19 vs. Population (transethnic - release 5) | 36,590 | 1,668,938 | 8.91 x 10^-6^ |
| rs60771488 | 16 | 56145518 | 9.26 x 10^-4^ | 0.12 | RP11-461O7.1; RP11-355E10.1 | 32404885 | COVID-19 vs. Population (transethnic - release 5) | 36,590 | 1,668,938 | 3.87 x 10^-7^ |
| rs62058964 | 17 | 43999406 | 7.10 x 10^-5^ | -0.06 | MAPT; AC217779.2 | 32404885 | Hospitalized COVID-19 vs. Population (European - release 5) | 9,986 | 1,877,672 | 6.16 x 10^-7^ |
| rs9890316 | 17 | 80443309 | 0.40 | -0.01 | NARF; NARF-IT | 32404885 | Hospitalized COVID-19 vs. Non-Hospitalized COVID-19 (transethnic - release 4) | 2,430 | 8,478 | 7.96 x 10^-6^ |
| rs4528629 | 17 | 71768878 | 0.38 | 0.01 | LINC00469 | 32404885 | Very severe respiratory confirmed COVID-19 vs. Population (transethnic - release 5) | 5,870 | 1,155,203 | 2.67 x 10^-6^ |
| rs141909101 | 17 | 80168281 | 0.54 | 0.03 | CCDC57; RP13-516M14.2 | 32404885 | Hospitalized COVID-19 vs. Non-Hospitalized COVID-19 (transethnic - release 4) | 2,430 | 8,478 | 5.68 x 10^-7^ |
| rs112159047 | 17 | 77407992 | 0.02 | 0.04 | RBFOX3 | 32404885 | Hospitalized COVID-19 vs. Population (European - release 5) | 9,986 | 1,877,672 | 5.08 x 10^-7^ |
| rs77534576 | 17 | 47940666 | 4.41 x 10^-5^ | 0.14 | - | 32404885 | Hospitalized COVID-19 vs. Population (European - release 5) | 9,986 | 1,877,672 | 5.78 x 10^-6^ |
| rs145459064 | 17 | 4955924 | 3.51 x 10^-6^ | 0.15 | SLC52A1; USP6; SCIMP; ZNF232; CTD-2369P2.10; CTD-2369P2.12 | 32404885 | COVID-19 vs. Population (transethnic - release 5) | 36,590 | 1,668,938 | 5.22 x 10^-6^ |
| rs142257532 | 18 | 30006171 | 0.77 | 0.01 | GAREM | 32404885 | Hospitalized COVID-19 vs. Non-Hospitalized COVID-19 (transethnic - release 4) | 2,430 | 8,478 | 5.54 x 10^-6^ |
| rs8096865 | 18 | 8430248 | 0.04 | 0.07 | - | 32404885 | Hospitalized COVID-19 vs. Population (European - release 5) | 9,986 | 1,877,672 | 4.40 x 10^-6^ |
| rs17069506 | 18 | 59821843 | 4.10 x 10^-4^ | 0.10 | PIGN | 32404885 | Hospitalized COVID-19 vs. Population (European - release 5) | 9,986 | 1,877,672 | 1.74 x 10^-7^ |
| rs8110610 | 19 | 49321911 | 5.38 x 10^-5^ | -0.06 | HSD17B14 | 32404885 | COVID-19 vs. Population (transethnic - release 5) | 36,590 | 1,668,938 | 6.34 x 10^-6^ |
| rs8101195 | 19 | 10423815 | 0.02 | -0.03 | RAVER1; FDX1L; ZGLP1; | 32404885 | Very severe respiratory confirmed COVID-19 vs. Population (transethnic - release 5) | 5,870 | 1,155,203 | 3.13 x 10^-6^ |
| rs10421782 | 19 | 4685276 | 0.01 | -0.03 | DPP9; AC005594.3 | 32404885 | Very severe respiratory confirmed COVID-19 vs. Population (transethnic - release 5) | 5,870 | 1,155,203 | 4.46 x 10^-6^ |
| rs2042908 | 19 | 53626513 | 0.33 | -0.01 | ZNF415; ZNF347 | 32404885 | Very severe respiratory confirmed COVID-19 vs. Population (transethnic - release 5) | 5,870 | 1,155,203 | 3.20 x 10^-6^ |
| rs7260056 | 19 | 4582762 | 0.81 | 3.87 x 10^-3^ | - | 32404885 | Very severe respiratory confirmed COVID-19 vs. Population (transethnic - release 5) | 5,870 | 1,155,203 | 1.50 x 10^-6^ |
| rs676314 | 19 | 50865535 | 2.13 x 10^-3^ | 0.04 | NAPSA; NR1H2; CTB-191K22.6 | 32404885 | Very severe respiratory confirmed COVID-19 vs. Population (transethnic - release 5) | 5,870 | 1,155,203 | 6.06 x 10^-6^ |
| rs2109069 | 19 | 4719443 | 3.01 x 10^-8^ | 0.07 | DPP9 | 32404885 | COVID-19 vs. Population (transethnic - release 5) | 36,590 | 1,668,938 | 2.76 x 10^-8^ |
| rs118115488 | 19 | 10556897 | 4.82 x 10^-4^ | 0.08 | PDE4A | 32404885 | COVID-19 vs. Population (transethnic - release 5) | 36,590 | 1,668,938 | 6.05 x 10^-6^ |
| rs880278 | 20 | 3740079 | 1.98 x 10^-3^ | 0.08 | C20orf27 | 32404885 | COVID-19 vs. Population (transethnic - release 5) | 36,590 | 1,668,938 | 5.91 x 10^-6^ |
| rs8132172 | 21 | 34574057 | 0.12 | -0.02 | - | 32404885 | Hospitalized COVID-19 vs. Population (European - release 5) | 9,986 | 1,877,672 | 2.06 x 10^-6^ |
| rs17860169 | 21 | 34613301 | 5.40 x 10^-6^ | 0.05 | IFNAR2; IL10RB; AP000295.9; IFNAR2 | 32404885 | COVID-19 vs. Population (transethnic - release 5) | 36,590 | 1,668,938 | 1.03 x 10^-8^ |

Note: BP, base pair position; Cases (N), number of cases in original study; CHR, chromosome; Controls (N), number of controls in original study; Gene, Gene mapping according to Ensembl VEP/FUMA v1.3.6a; OR, odds ratio in data used for effect sizes in creating PRS; OS P-value of SNP association with COVID-19 phenotype in original study; OS, original study source for SNP association with COVID-19 phenotype; P, p-value in data used for effect sizes in creating PRS; SNP, single nucleotide polymorphism; PRS_e2_, White European polygenic risk score 2.

### Supplementary Table S20: Results of gene-set enrichment analysis for genes encoded by SNPs of PRS_e2_. Conducted by FUMA v1.3.6a using Reactome database.

| Gene Set | *N* | n | *P*-value | Adjusted *P*-value | Genes |
| --- | --- | --- | --- | --- | --- |
| OAS antiviral response | 9 | 3 | 3.89e-7 | 5.84e-4 | OAS1, OAS3, OAS2 |
| Interferon alpha-beta signalling | 62 | 4 | 3.80e-6 | 2.85e-3 | OAS1, OAS3, OAS2, IFNAR2, AP000295.9 |
| Interleukin-10 signalling | 45 | 3 | 6.30e-5 | 2.87e-2 | IL10RB, CCR2, CCR5 |
| Chemokine receptors bind chemokines | 48 | 3 | 7.66e-5 | 2.87e-2 | CCR3, CCR2, CCR5 |

Note: FUMA, Functional Mapping and Annotation of Genome-Wide Association Studies; Gene Set, name of gene set in Reactome; N, total number of genes in Reactome gene set; n, number of genes from gene set found in PRS_e2_; OAS, Oligoadenylate Synthetase; PRS_e2_, White European polygenic risk score 2; SNP, single nucleotide polymorphism

Supplementary Figure S2. Kaplan Meier curves of severe COVID-19 (hospitalisation, critical admission or death) in patients diagnosed with COVID-19, per demographics, immunosuppressant use status, comorbidity count and White European polygenic risk score 2 quintile (*N=*9,560)


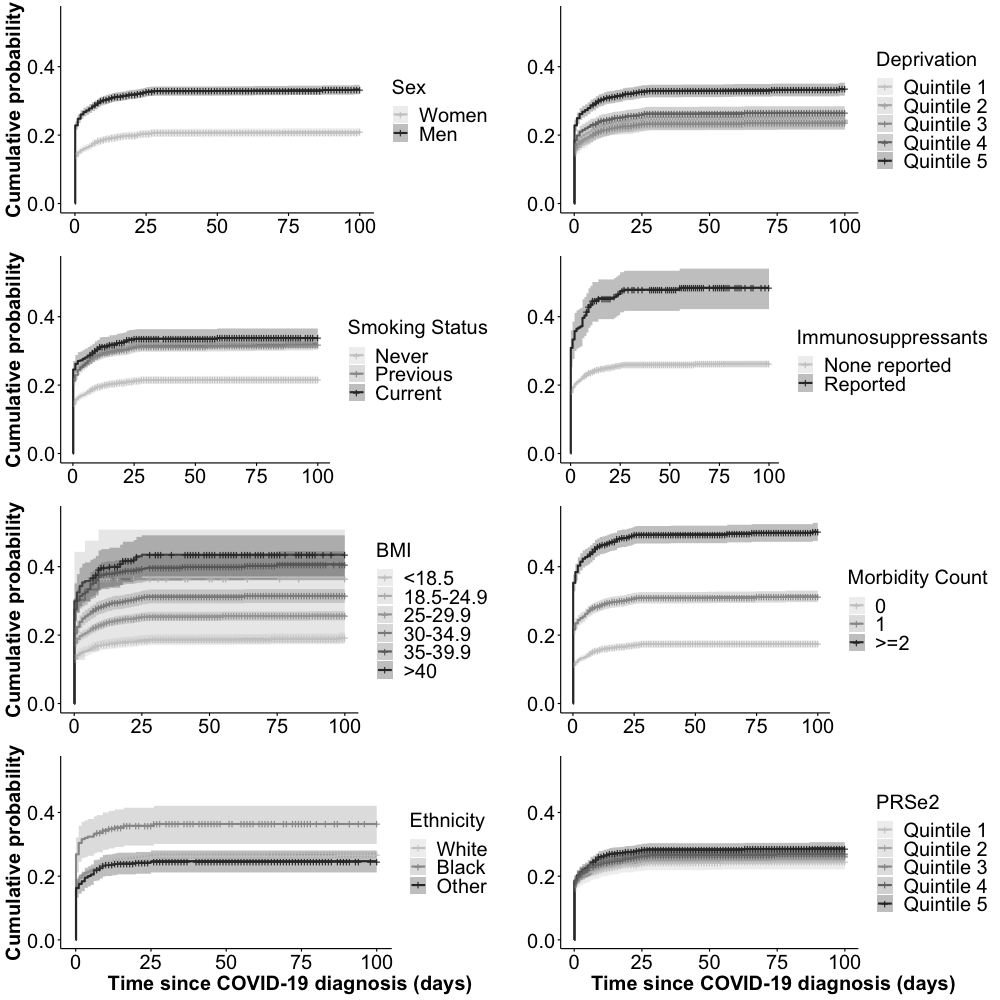
Note: for each patient, the earliest of recorded hospitalisations or critical care admissions within 28 days of COVID-19 diagnosis, or death within 100 days of COVID-19 diagnosis is considered.

BMI, body mass index (in kg/m^2^); PRS_e2_, White European polygenic risk score 2

Supplementary Figure S3. Kaplan Meier curves of death in patients diagnosed with COVID-19, per demographics, immunosuppressant use status, comorbidity count and White European polygenic risk score 2 quintile (*N=*9,560)


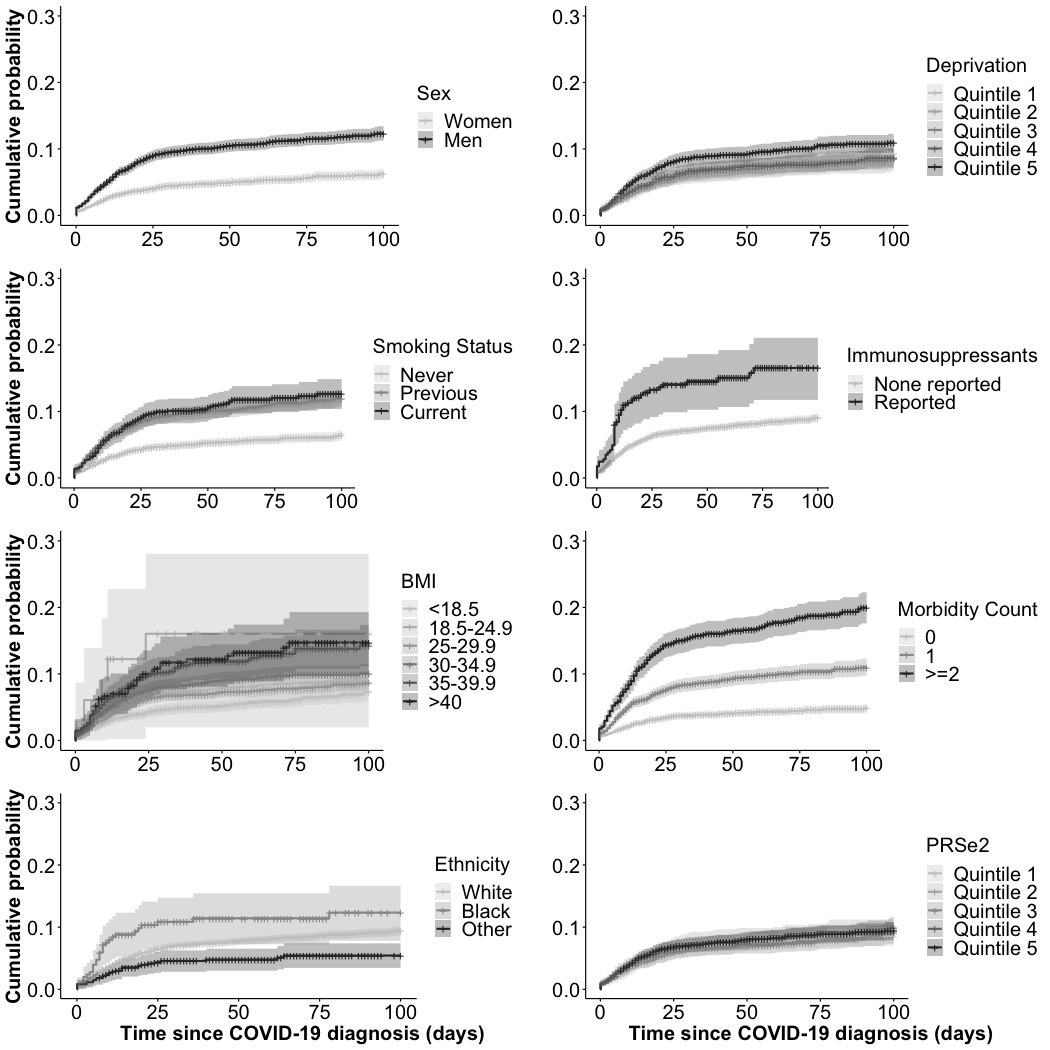


Note: BMI, body mass index (in kg/m^2^); PRS_e2_, White European polygenic risk score 2

Supplementary Figure S4. Kaplan Meier curve of hospitalisation, critical admission or death in the White European subpopulation diagnosed with COVID-19, per demographics, White European polygenic risk score 2 quintile and immunosuppressant use status (*N=*7,274)


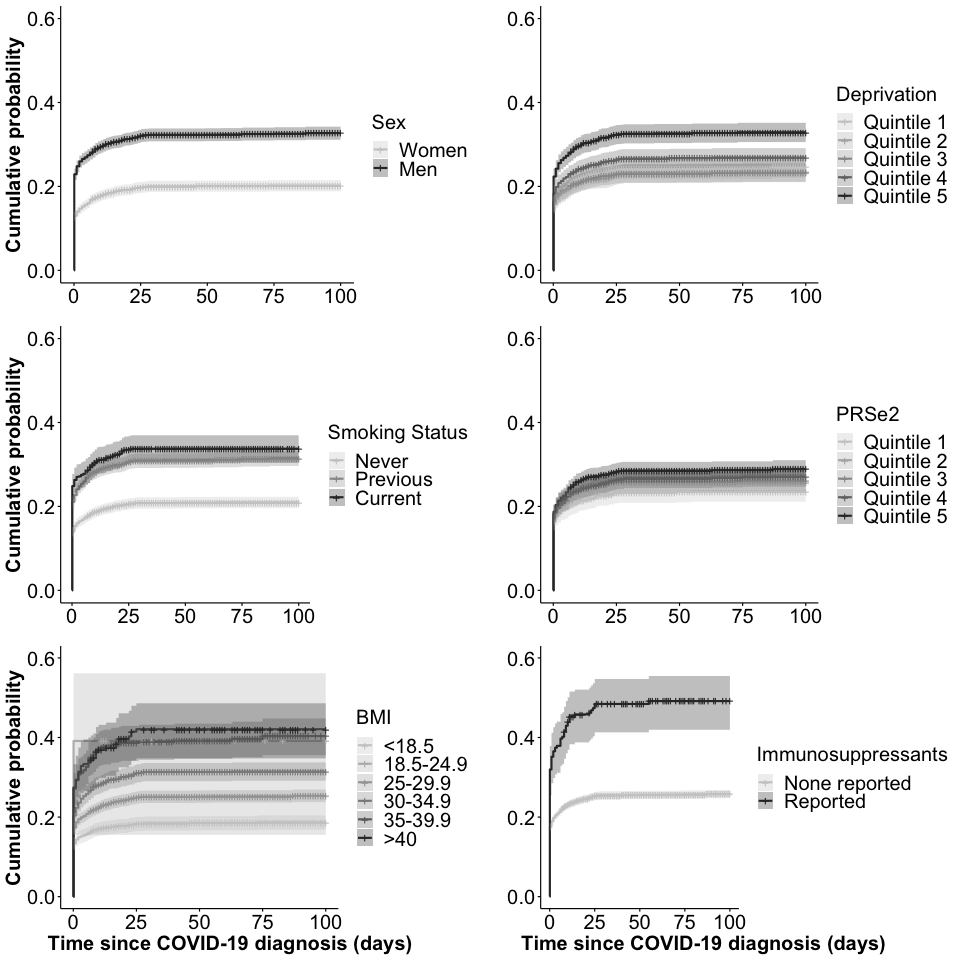


Note: for each patient, the earliest of recorded hospitalisations or critical care admissions within 28 days of COVID-19 diagnosis, or death within 100 days of COVID-19 diagnosis is considered.

BMI, body mass index (in kg/m^2^); PRS_e2_, White European polygenic risk score 2

Supplementary Figure S5. Kaplan Meier curve of death in the White European subpopulation diagnosed with COVID-19, per demographic factor, immunosuppressant use status and White European polygenic risk score 2quintile (*N=*7,274)


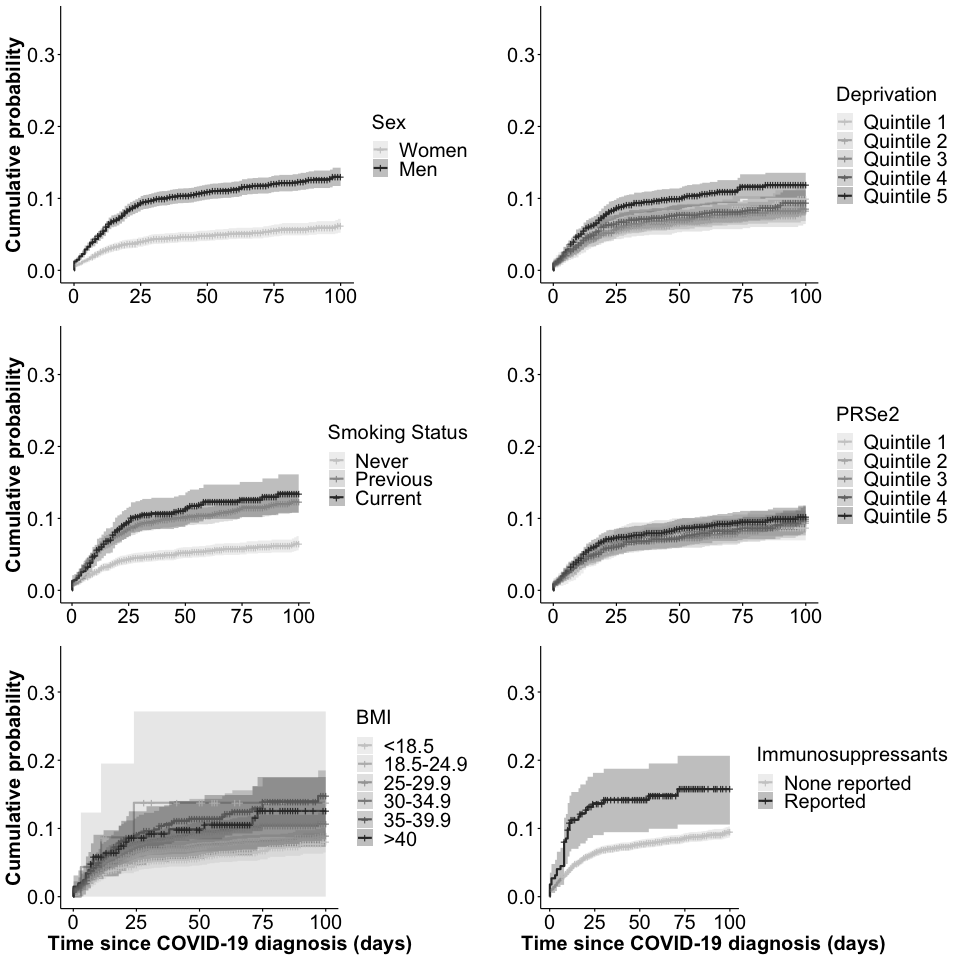


Note: BMI, body mass index (in kg/m^2^); PRS_e2_, White European polygenic risk score 2

### Supplementary Figure S6. Manhattan plot for the MAGMA (de Leeuw 2016) gene-based test. Red dotted line represents the –log_10_ genome-wide significance value for 0.05/19,254 = 2.60 x 10^-6^. Enriched genes which pass this level of significance are labelled.


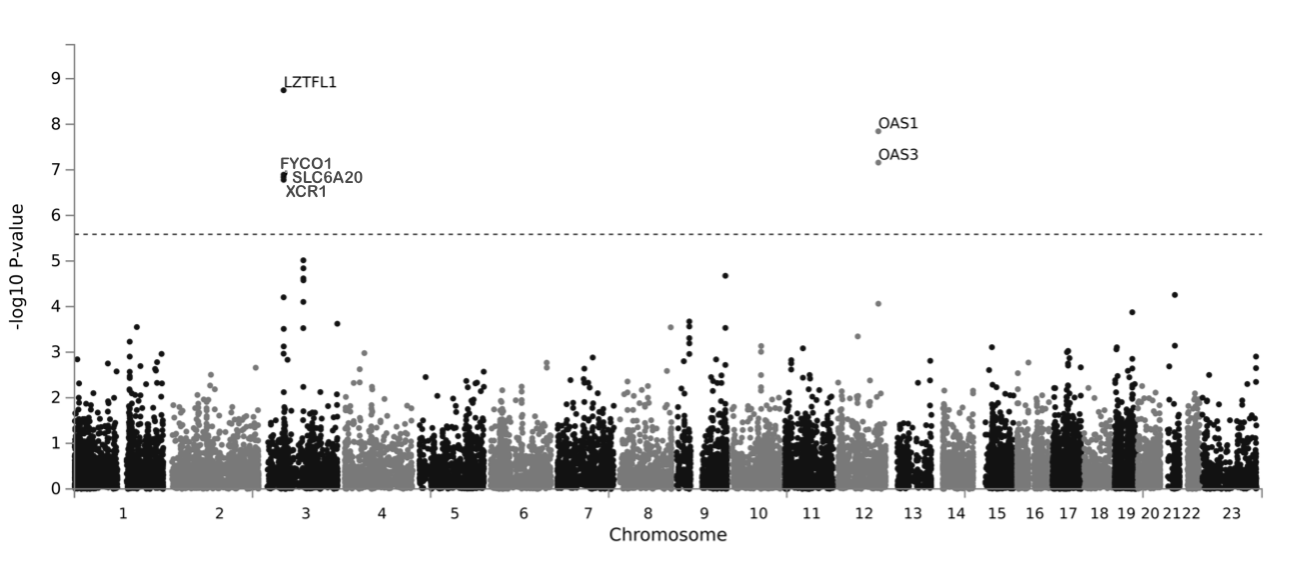


## References

1. Bycroft C, Freeman C, Petkova D, Band G, Elliott LT, Sharp K, et al. The UK Biobank resource with deep phenotyping and genomic data. Nature. 2018;562(7726):203-9.

2. McCarthy S, Das S, Kretzschmar W, Delaneau O, Wood AR, Teumer A, et al. A reference panel of 64,976 haplotypes for genotype imputation. Nature genetics. 2016;48(10):1279.

3. Consortium GP. An integrated map of genetic variation from 1,092 human genomes. Nature. 2012;491(7422):56-65.

4. Bellenguez C, Strange A, Freeman C, Consortium† WTCC, Donnelly P, Spencer CC. A robust clustering algorithm for identifying problematic samples in genome-wide association studies. Bioinformatics. 2012;28(1):134-5.

5. Initiative C-HG. Mapping the human genetic architecture of COVID-19. Nature. 2021.

6. Bulik-Sullivan BK, Loh P-R, Finucane HK, Ripke S, Yang J, Patterson N, et al. LD Score regression distinguishes confounding from polygenicity in genome-wide association studies. Nature genetics. 2015;47(3):291-5.

7. Bulik-Sullivan B, Finucane HK, Anttila V, Gusev A, Day FR, Loh P-R, et al. An atlas of genetic correlations across human diseases and traits. Nature genetics. 2015;47(11):1236-41.

8. Choi SW, Mak TS-H, O’Reilly PF. Tutorial: a guide to performing polygenic risk score analyses. Nature Protocols. 2020;15(9):2759-72.

9. Group SC-G. Genomewide association study of severe Covid-19 with respiratory failure. New England Journal of Medicine. 2020;383(16):1522-34.

10. Pairo-Castineira E, Clohisey S, Klaric L, Bretherick AD, Rawlik K, Pasko D, et al. Genetic mechanisms of critical illness in Covid-19. Nature. 2021;591(7848):92-8.

11. Hu J, Li C, Wang S, Li T, Zhang H. Genetic variants are identified to increase risk of COVID-19 related mortality from UK Biobank data. Human genomics. 2021;15(1):1-10.

12. Buniello A, MacArthur JAL, Cerezo M, Harris LW, Hayhurst J, Malangone C, et al. The NHGRI-EBI GWAS Catalog of published genome-wide association studies, targeted arrays and summary statistics 2019. Nucleic acids research. 2019;47(D1):D1005-D12.

13. Lyon MS, Andrews SJ, Elsworth B, Gaunt TR, Hemani G, Marcora E. The variant call format provides efficient and robust storage of GWAS summary statistics. Genome biology. 2021;22(1):1-10.

14. Dite GS, Murphy NM, Allman R. An integrated clinical and genetic model for predicting risk of severe COVID-19: A population-based case–control study. PloS one. 2021;16(2):e0247205.

15. McLaren W, Gil L, Hunt SE, Riat HS, Ritchie GR, Thormann A, et al. The ensembl variant effect predictor. Genome biology. 2016;17(1):1-14.

16. Watanabe K, Taskesen E, Van Bochoven A, Posthuma D. Functional mapping and annotation of genetic associations with FUMA. Nature communications. 2017;8(1):1-11.

17. Wang K, Li M, Hakonarson H. ANNOVAR: functional annotation of genetic variants from high-throughput sequencing data. Nucleic acids research. 2010;38(16):e164-e.

18. de Leeuw CA, Stringer S, Dekkers IA, Heskes T, Posthuma D. Conditional and interaction gene-set analysis reveals novel functional pathways for blood pressure. Nature communications. 2018;9(1):1-13.

19. Griss J, Viteri G, Sidiropoulos K, Nguyen V, Fabregat A, Hermjakob H. Reactomegsa-efficient multi-omics comparative pathway analysis. Molecular

Cellular Proteomics. 2020;19(12):2115-25.

20. Martens M, Ammar A, Riutta A, Waagmeester A, Slenter DN, Hanspers K, et al. WikiPathways: connecting communities. Nucleic Acids Research. 2021;49(D1):D613-D21.
